# Analysis of the* in planta* transcriptome expressed by the corn pathogen *Pantoea stewartii* subsp. *stewartii* via RNA-Seq

**DOI:** 10.7717/peerj.3237

**Published:** 2017-04-27

**Authors:** Holly Packard, Alison Kernell Burke, Roderick V. Jensen, Ann M. Stevens

**Affiliations:** Department of Biological Sciences, Virginia Polytechnic Institute and State University (Virginia Tech), Blacksburg, VA, United States

**Keywords:** Transcriptome analysis, *Pantoea stewartii*, Phytopathogen, Corn, RNA-Seq

## Abstract

*Pantoea stewartii* subsp. *stewartii* is a bacterial phytopathogen that causes Stewart’s wilt disease in corn. It uses quorum sensing to regulate expression of some genes involved in virulence in a cell density-dependent manner as the bacterial population grows from small numbers at the initial infection site in the leaf apoplast to high cell numbers in the xylem where it forms a biofilm. There are also other genes important for pathogenesis not under quorum-sensing control such as a Type III secretion system. The purpose of this study was to compare gene expression during an *in planta* infection versus either a pre-inoculum *in vitro* liquid culture or an *in vitro* agar plate culture to identify genes specifically expressed *in planta* that may also be important for colonization and/or virulence. RNA was purified from each sample type to determine the transcriptome via RNA-Seq using Illumina sequencing of cDNA. Fold gene expression changes in the *in planta* data set in comparison to the two *in vitro* grown samples were determined and a list of the most differentially expressed genes was generated to elucidate genes important for plant association. Quantitative reverse transcription PCR (qRT-PCR) was used to validate expression patterns for a select subset of genes. Analysis of the transcriptome data via gene ontology revealed that bacterial transporters and systems important for oxidation reduction processes appear to play a critical role for *P. stewartii* as it colonizes and causes wilt disease in corn plants.

## Introduction

*Pantoea stewartii* subsp. *stewartii* is a Gram-negative gamma-proteobacterium that causes Stewart’s wilt disease in corn plants. After *P. stewartii* enters the corn plant through wounds created during feeding by the corn flea beetle, *Chaetocnema pulicaria*, it first causes water-soaked lesions within the leaves of the plant. Once within the leaf apoplast, the bacteria travel to the xylem of the plant where they can then proliferate and form a biofilm containing the exopolysaccharide stewartan. Biofilm formation blocks water transport within the xylem leading to wilting and even death of seedlings (Bradshaw-Rouse et al., 1981). More specifically, the biofilm buildup leads to rupturing of the pit membrane between xylem cells, which normally prevents vascular pathogen passage, enabling the continued spread of the bacteria in a systemic manner while simultaneously inhibiting water transport (Choat, Cobb & Jansen, 2008). Although resistant corn hybrids have emerged in the last 50 years, there are still areas where partially and fully susceptible cultivars are grown (Freeman & Pataky, 2001). The disease is native to North America, but can be transmitted to the seeds from the infected parent plant, therefore thorough examination of the seeds must occur before exportation (Michener, Pataky & White, 2002).

The lifestyle of *P. stewartii* requires precise temporal control of colonization and virulence factor expression in the plant host. Quorum-sensing regulation is known to enable the transition from low bacterial density in the leaf apoplast to high bacterial density in the xylem. This cell–cell communication occurs in response to the production of population density-dependent signals. The master quorum-sensing regulator, EsaR, is active at low cell density, but rendered inactive at high cell density in the presence of the acyl-homoserine lactone signal, 3-oxo-C6-homoserine lactone (von Bodman, Majerczak & Coplin, 1998). Thus one subset of genes in *P. stewartii* is activated or repressed at low cell density, but then these same genes will be deactivated or derepressed, respectively, at high cell density (Beck von Bodman & Farrand , 1995; Schu et al., 2009). Quorum sensing has been demonstrated to directly regulate genes important for exopolysaccharide production, adhesion/motility and stress response (Ramachandran et al., 2014), including the second tier transcription factors RcsA and LrhA, whose regulons are important for virulence (Kernell Burke et al., 2015).

Other virulence factors in *P. stewartii* appear to be expressed independently of the quorum-sensing response. For example, *hrp* (hypersensitive response and pathogenicity) genes that encode for type III secretion system (T3SS) and effector proteins are also activated during infection (Frederick et al., 2001; Correa et al., 2012). The WtsE (water soaking) effector protein is regulated as part of the HrpL regulon (Merighi et al., 2003), and is responsible for disrupting the host cell membrane, leading to the buildup of fluids characteristic of the water-soaking symptom (Ham et al., 2006). In addition to water soaking, WtsE is also responsible for altering the metabolome within the plant, specifically inducing gene expression for the phenylpropanoid pathway (Asselin et al., 2015). Disrupting this pathway influences the ability of the plant to accumulate lignin during the hypersensitive response, produce salicylic acid, and maintain plant defense signaling, indicating this alteration is a key factor in *P. stewartii* success (Asselin et al., 2015).

To better understand the interactions occurring between the corn plant and *P. stewartii*, and how this influences expression of genes required for pathogen survival within the host, an analysis of *in planta* bacterial gene expression was performed. It was hypothesized that comparing *in planta* transcriptome levels to those of the bacteria in a pre-inoculum *in vitro* liquid culture (low cell density planktonic growth) or an *in vitro* agar plate culture (high cell density surface growth) would reveal genes required exclusively for host colonization and infection. Genes of interest identified through studies of *P. stewartii* may serve as targets for disease intervention strategies and have implications for understanding other xylem-dwelling and/or wilt disease-causing bacterial phytopathogens.

## Materials and Methods

### Bacterial strains and media

Strains of *P. stewartii* and *Escherichia coli* that were used in this study are listed in Table S1. Luria Bertani broth (LB; 10 g/L tryptone, 5 g/L NaCl, 5 g/L yeast extract) or 1.5% agar plates were used for all *E. coli* strains, while both LB and Rich Minimal medium (RM; 1X M9 salts, 2% casamino acids, 1 mM MgCl_2_, 0.4% glucose) were used for *P. stewartii* growth. *E. coli* strains were grown at 37 °C and *P. stewartii* strains were grown at 30 °C. Growth media were supplemented with nalidixic acid (30 µg/mL) or ampicillin (100 µg/mL) when required (Table S1).

### Growth of cells for RNA-Seq analysis

Three different conditions were used to grow duplicate samples of *P. stewartii* for RNA-Seq analysis. First, a liquid culture of *P. stewartii* DC283 was grown overnight shaking at 30 °C in LB medium supplemented with 30 µg/mL nalidixic acid. This was subcultured to an optical density (OD_600_) of 0.05 in 5 mL of the same medium and then grown to an OD_600_ of 0.2. The cells were pelleted by centrifugation (Eppendorf centrifuge 5424, rotor 5424R) for 1 min at 10,000 rpm and washed in phosphate buffered saline solution (PBS; 137 mM NaCl, 2.7 mM KCl, 10 mM Na_2_HPO_4_ and 2 mM KH_2_PO_4_, pH 7.4) followed by a second centrifugation step. RNA Protect Bacterial Reagent (Qiagen, Hilden, Germany)(5 mL) was used to resuspend the pellet. After a brief vortex and 5 min incubation at room temperature, centrifugation was again performed and the pellet was stored at −20 °C temporarily until RNA was extracted.

The second set of samples was comprised of agar-grown bacteria. A culture of *P. stewartii* DC283 was grown overnight shaking at 30 °C in RM liquid medium supplemented with 3 µg/mL nalidixic acid. This was subcultured the following day to an OD_600_ of 0.05. A 100 µL volume of this was spread onto a RM medium 1.5% agar plate with 30 µg/mL nalidixic acid and then incubated at 30 °C for 18 h. The plate culture was harvested by using 5 mL of RNA Protect Bacterial Reagent to flood the plate, and then the cells were gently scraped and pipetted off of the plate and into a microcentrifuge tube. This sample was briefly vortexed, incubated at room temperature for 5 min, pelleted via centrifugation for 1 min at 10,000 rpm as described above and the pellet was stored at −20 °C prior to RNA extraction.

Third, *in planta* bacterial samples were prepared. *Zea mays* ‘Jubilee’ corn seeds (HPS Seed) were planted and grown for five days in a Percival Scientific plant chamber at 28 °C, 80% humidity, 16 h light and 8 h dark cycles, and at least 200 mE m^−2^ s^−1^ light intensity in Sunshine Mix #1 soil. On day four, *P. stewartii* DC283 was grown overnight in LB medium supplemented with 30 µg/mL nalidixic acid in 30 °C. This was subcultured on day five to an OD_600_ of 0.05, and then grown to an OD_600_ of 0.2. One mL of the culture was harvested, via centrifugation for 1 min at 10,000 rpm, and washed in PBS. Average-sized healthy seedlings were surface washed with 70% ethanol at the point of inoculation at the base of the stem, then scratched with a sterile syringe needle (one cm in length and one cm above the soil) deep enough to reach the plant xylem, and 5 µL of culture resuspended in PBS were pipetted into the scratch area. The plants were grown for ten more days before harvesting. The plant stem and a razor blade were washed with ethanol, and then the stem was cut at the soil line and again at the top before leaf branching occurred. The stem was placed in RNA Protect Bacterial Reagent and a pipette was used to draw 1 mL up through the stalk to extract the biofilm. Samples, composed of material recovered from two stems, were then briefly vortexed, incubated at room temperature for 5 min, and bacterial cells were pelleted via centrifugation at 10,000 rpm for 1 min in an Eppendorf centrifuge 4524. Pellets were stored at −20 °C prior to RNA extraction.

### RNA purification and RNA-Seq

Frozen cell pellets were resuspended in 100 µL TE buffer (10 mM Tris-HCl, 1 mM EDTA, pH 7.0) containing 15 mg/mL lysozyme and 30 mAU/mL of Proteinase K (Qiagen), as previously described (Ramachandran et al., 2014). After resuspension of the pellet, the miRNeasy kit (Qiagen) was used to extract total RNA per the recommended manufacturer’s protocol. Quality of RNA was determined at the Virginia Tech Biocomplexity Institute (VTBI) via the Agilent Bioanalyzer 2100, and a minimal RNA integrity number (RIN) of 7.0 was required for continued analysis. Ribosomal RNA (rRNA) was removed from the sample using a RiboZero Bacteria kit (Illumina, San Diego, CA, USA) and HiSeq 2500 100 nt single-end read Illumina sequencing was performed at the University of Illinois Roy J. Carver Biotechnology Center.

### RNA-Seq data analysis

Data preparation and analysis were performed based on a previously published protocol (Kernell Burke et al., 2015). Briefly, the data was downloaded and unzipped into the Geneious software (version 8.3.1) to align to the coding sequences annotated in the WGS reference sequence AHIE00000000.1 for *Pantoea stewartii* subsp. *stewartii* DC283 from NCBI. Thus, the small amount of plant sequences in the samples were eliminated from the analysis. Normalization of individual read counts for genes to the total number of mapped sequence reads via reads per million (RPM) was performed in Microsoft Excel. Due to the high similarity of the many transposase sequences in the *Pantoea* genome, all transposases and IS66 family insertion sequences were excluded from the read normalization analysis. RPM expression values were then compared between each sample through ratios.

In addition, the Bioconductor software package “DESeq2” (Love, Huber & Anders, 2014) was used in R (version 3.2.4) to analyze the raw read counts with a more sophisticated gene expression normalization and error model to estimate the statistical significance of detected gene expression changes by calculating multiple testing adjusted *p*-values. The fold changes (DESeq Fold Regulation) determined by this second method overlapped to a large extent with our Microsoft Excel analysis for the genes with four-fold or greater change (Tables S2 and S3). The adjusted *p*-values (DESeq padj) for those gene selected for qRT-PCR validation were all less than 0.023.

Genes chosen from this dataset for qRT-PCR validation for the *in planta* culture and pre-inoculum *in vitro* liquid culture comparison were selected based upon previous standards (Kernell Burke et al., 2015). These genes each had greater than 100 reads for at least one of the samples, there was at least a four-fold change in expression between the two sample types compared, and there was no more than a two-fold change between the two replicates for each sample type. From the list of genes that met the above criteria, ten genes were chosen for qRT-PCR validation based upon their biological function. These same ten genes were used for validation for the *in planta* culture and *in vitro* plate culture comparison. Three of the genes fell below the four-fold change in expression threshold, but were still included in this second analysis. Three control genes were selected based on stable housekeeping function, with at most a two-fold difference between the replicates, and less than a two-fold change between the sample types.

### Cloning of coding regions of genes of interest for primer optimization

The coding region of the genes selected for qRT-PCR validation of the RNA-Seq data were cloned into pGEM-T (Promega). PCR was performed with 1X ThermoPol Buffer, 200 µM dNTP, 1.25 units/ 50 µL of *Taq* Polymerase, 0.2 µM of each primer (Table S4), and *P. stewartii* DC283 chromosomal DNA template. Thermocycler settings per enzyme protocol (New England Biolabs) were denaturation at 95 °C for 30 s, annealing for 60 s at the appropriate temperature (Table S4), and extension at 68 °C for 30 s, performed for 30 cycles. The final extension was 68 °C for 5 min. The PCR products were visualized on a 1% agarose gel, and extracted using a Gel Extraction Kit (Qiagen). Fragments were modified by addition of dATP via *Taq* polymerase and a PCR Purification Kit (Qiagen) was used to remove additional dATPs. This PCR product was then ligated into the pGEM-T vector (Promega, Madison, WI, USA) and the resulting plasmid was transformed into *E. coli* Top 10 (Table S1). Plasmids containing the coding regions were screened via PCR and sequenced (VTBI) to confirm the construct.

### Quantitative reverse transcription PCR (qRT-PCR)

The qRT-PCR primers for the genes of interest (Table S4) were designed by Primer Express, version 3.0 (Life Technologies, Carlsbad, CA, USA) to amplify approximately 100 bp segments from regions with uniform coverage in the RNA-Seq reads as confirmed using Geneious software. For primer optimization (90–110% efficiency) and qRT-PCR (Applied Biosystems 7300 Real-Time PCR System), the primers were all at 0.4 µM concentration, with the exception of CKS_3793 (0.6 µM), as this was optimized from a previous study (Kernell Burke et al., 2015). RNA for each sample type was harvested using the same methods as for the RNA-Seq following the miRNeasy kit protocol. Each sample had a RIN value of at least 7. Once extracted, the RNA was converted to cDNA using the ABI High Capacity cDNA Reverse Transcription kit (Thermo Fisher Scientific, Waltham, MA, USA). The Pfaffl method was used to determine the fold change differences between samples from the *in planta* culture and either the pre-inoculum *in vitro* liquid culture or *in vitro* plate culture.

### Gene ontology

Gene ontology (GO) analysis was performed using topGO software (Alexa & Rahnenfuhrer, 2016) in R version 3.3.0 (Bioconductor). The genes from the RNA-Seq data that were four-fold or more differentially expressed between the *in planta* culture and pre-inoculum *in vitro* liquid culture were separated into genes that were upregulated or downregulated *in planta*. This was repeated for the *in planta* comparison with the *in vitro* plate culture. Fisher’s exact test was used for statistical analysis, specifically using the default “weight01” algorithm for processing the datasets (Alexa, Rahnenfuhrer & Lengauer, 2006). Analysis focused on groups of genes enriched for the Biological Process (BP) gene ontologies. Gene groups with *p*-values of 0.01 or lower were considered significantly regulated within the dataset.

### Accession numbers

The read data for the pairs of duplicate samples of the *P. stewartii* DC283 cells from the *in planta* culture, the pre-inoculum *in vitro* liquid culture, and the *in vitro* plate culture have been deposited in the NCBI Sequence Read Archive (SRA) with accession numbers, GSM2333085, GSM2333086, GSM2333087, GSM2333088, GSM2333089 and GSM2333090, respectively. An Excel file summarizing the total read counts for each sample using the *P. stewartii* DC283 version 8 sequence annotations from NCBI was deposited into the NCBI Gene Expression Omnibus (Edgar, Domrachev & Lash, 2003) database (GEO Accession GSE87520).

## Results

### Comparison of RNA-Seq data reveals genes important for *in planta* colonization and growth

**Figure 1:**
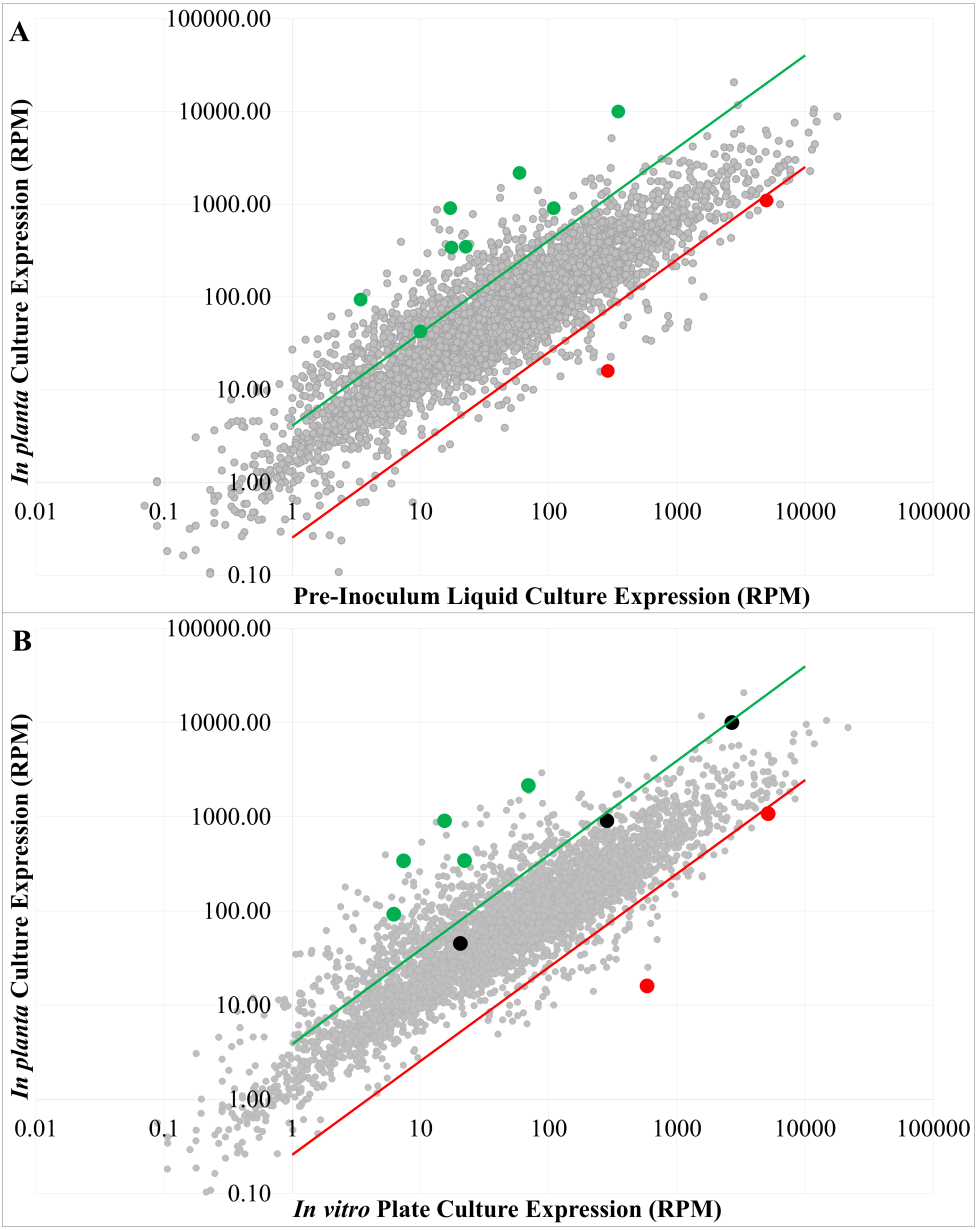
Differential mRNA expression *in planta.* Whole transcriptome data (averaged normalized RPM) of the *P. stewartii* DC283 strain grown *in planta* versus the pre-inoculum *in vitro* liquid culture (A) or an *in vitro* plate culture (B). A gray filled circle is used to represent each gene. The green and red lines represent the four-fold expression ratio cutoff, where any points that fall outside of them are considered upregulated (green) or downregulated (red) *in planta*. Genes validated through qRT-PCR are represented as filled green (upregulated), red (downregulated), or black (below the four-fold regulation parameter) circles.

RNA-Seq was performed on wild-type *P. stewartii* DC283 RNA extracted, in duplicate, from an *in planta* infection culture, a pre-inoculum *in vitro* liquid culture, and an *in vitro* agar plate culture in order to determine genes differentially expressed during an *in planta* infection versus *in vitro* culture conditions. Raw RNA-Seq reads of 100 bp length yielded an average of between 32.7 and 39.2 million reads for the different samples. The normalized RPM counts for each data set were calculated, replicates were averaged, and the fold change of differential regulation between two different growth conditions was determined for each gene. Genes with four-fold or greater increased RPM expression levels *in planta* compared to the pre-inoculum liquid or *in vitro* plate cultures were considered upregulated, while those whose expression levels were decreased four-fold or more *in planta* were considered downregulated (Fig. 1).

There were 528 genes (roughly 10% of the genome) that had a minimum of four-fold differential RPM expression between the *in planta* set and the pre-inoculum *in vitro* liquid culture set (Table S2). The highest fold RPM change for an annotated gene was about a 53-fold higher *in planta* compared to the pre-inoculum liquid culture for gene CKS_3263, annotated as an HrpA family pilus protein. Comparing the *in planta* data set with the *in vitro* plate culture set yielded 530 differentially expressed genes (Table S3) with a minimum of four-fold differential RPM expression. The highest fold RPM for an annotated gene was almost 70-fold higher *in planta* compared to the *in vitro* plate culture for gene CKS_3355, annotated as a periplasmic-binding component of an ABC superfamily ribose transporter. Interestingly, there was a great deal of overlap between the most upregulated and downregulated genes between the *in planta* and pre-inoculum liquid culture comparison and the *in planta* and *in vitro* agar plate culture comparison. There were 357 genes found to be common in both comparisons, 308 upregulated and 49 downregulated (Table S5), indicating these genes are unique to plant colonization and infection. Results of a secondary analysis of the RNA-Seq data using DESeq2 (DESeq Fold Regulation) demonstrated that the DESeq results overlapped to a large degree with our Microsoft Excel RPM analysis for the genes with four-fold or greater change (Tables S2 and S3), and also provided impressive estimates of statistical significance. Only a relatively small number of additional new genes with four-fold or greater change were identified via DESeq (Table S6), thus the RPM results was used for subsequent analysis.

### Validation of the RNA-Seq via quantitative reverse transcription PCR

Ten genes that were regulated greater than four-fold via the RPM analysis (*p*-values (DESeq padj) < 0.023) or were of particular physiological interest as described below, were chosen to use for qRT-PCR validation of the *in planta* versus the pre-inoculum liquid culture RNA-Seq comparison (Table 1) and for the *in planta* versus the *in vitro* plate RNA-Seq comparison (Table 2) using a second independent set of RNA samples. Seven of these selected genes were differentially regulated four-fold or more in both comparisons. Three of the genes fell below the four-fold change in expression threshold in the *in planta* versus *in vitro* plate culture comparison, but they were still included in the qRT-PCR studies. Gene choice was also driven based in part upon putative annotated biological function. CKS_3263 and CKS_4537 were chosen because of their relation to the T3SS regulon, which would indicate if they were required during late stage infection. Transcriptional regulators encoded by CKS_3570, associated with stress response or pathogenicity (Gallegos et al., 1997), CKS_2505, associated with cellular metabolism, pili formation, and suspected in helping with persistence (Deng, Wang & Xie, 2011), and *hupA*, associated with DNA binding and regulation (Kohno et al., 1990), were chosen for validation, as well as *rmf*, which encodes a translational regulator seen to be activated during stationary phase in *E. coli* (Wada et al., 1990). Genes *aceB* and *yeaG* were chosen to look for metabolic changes that are *in planta* specific. CKS_3793 was chosen due to its normal function in microaerobic environments, hinting at the conditions within the plant xylem (Anraku & Gennis, 1987; Cotter et al., 1997). Finally, the *bfr* gene was chosen due to its role in iron acquisition during plant-pathogen interactions (Lawson et al., 2009). Control genes *recF*, *atpD*, and *gyrB* (Tables 1 and 2) were chosen based upon their housekeeping functions and stable expression levels under all three growth conditions.

**Table 1:** Genes from the RNA-Seq data comparing the *in planta* reads to the pre-inoculum *in vitro* liquid culture reads validated by qRT-PCR^a^.

Locus tag	Gene	Annotation		RPM fold regulation	DESeq fold regulation	DESeq padj
CKS_3263		HrpA family pilus protein	A	52.64	34.17	1.09E−85
CKS_3793		Cytochrome d ubiquinol oxidase subunit I	A	36.48	23.81	2.78E−59
CKS_4032	*rmf*	Ribosome modulation factor	A	28.23	17.29	3.37E−21
CKS_1591	*bfr*	Bacterioferritin iron storage and detoxification protein	A	27.19	17.89	1.27E−50
CKS_3570		AraC family transcriptional regulator	A	19.33	12.66	2.19E−33
CKS_4657	*aceB*	Malate synthase A	A	15.20	9.76	2.40E−20
CKS_2714	*yeaG*	Serine-protein kinase	A	8.22	5.66	1.38E−29
CKS_2505		AsnC family transcriptional regulator	A	4.20	2.89	1.45E−09
CKS_0004	*hupA*	HUD DNA-binding transcriptional regulator alpha subunit	R	4.58	6.30	2.94E−30
CKS_4537		T3SS effector protein	R	18.27	23.86	8.75E−59
CKS_4346	*recF*	Gap repair protein	Control	1.29	0.90	5.99E−01
CKS_1206	*atpD*	F1 sector of membrane-bound ATP synthase beta subunit	Control	1.14	0.62	2.51E−02
CKS_4345	*gyrB*	DNA gyrase subunit B	Control	1.08	0.65	3.95E−02

**Notes.**

a A = activated or R = repressed gene *in planta*.

**Table 2:** Genes from the RNA-Seq data comparing the *in planta* reads to the *in vitro* plate culture reads validated by qRT-PCR^a^.

Locus tag	Gene	Annotation		RPM fold regulation	DESeq fold regulation	DESeq padj
CKS_3263		HrpA family pilus protein	A	58.52	41.81	3.36E−90
CKS_3570		AraC family transcriptional regulator	A	45.70	32.15	2.94E−52
CKS_3793		Cytochrome d ubiquinol oxidase subunit I	A	31.45	11.51	7.77E−05
CKS_4657	*aceB*	Malate synthase A	A	15.65	11.35	1.23E−26
CKS_1591	*bfr*	Bacterioferritin iron storage and detoxification protein	A	14.97	10.99	1.79E−24
CKS_4032	*rmf*	Ribosome modulation factor	A	3.70^b^	2.84	5.81E−07
CKS_2714	*yeaG*	Serine-protein kinase	A	3.16^b^	2.42	2.80E−08
CKS_2505		AsnC family transcriptional regulator	A	2.13^b^	1.61	2.34E−02
CKS_0004	*hupA*	HUD DNA-binding transcriptional regulator alpha subunit	R	4.71	5.93	7.63E−19
CKS_4537		T3SS effector protein	R	36.62	44.52	1.97E−89
CKS_4346	*recF*	Gap repair protein	Control	1.07	0.82	2.77E−01
CKS_1206	*atpD*	F1 sector of membrane-bound ATP synthase beta subunit	Control	1.73	1.34	1.74E−01
CKS_4345	*gyrB*	DNA gyrase subunit B	Control	1.43	1.10	5.91E−01

**Notes.**

a A = activated or R = repressed gene *in planta* culture.

b Genes selected for *in planta* versus pre-inoculum liquid culture comparison (Table 1), but also included in this study.

**Figure 2:**
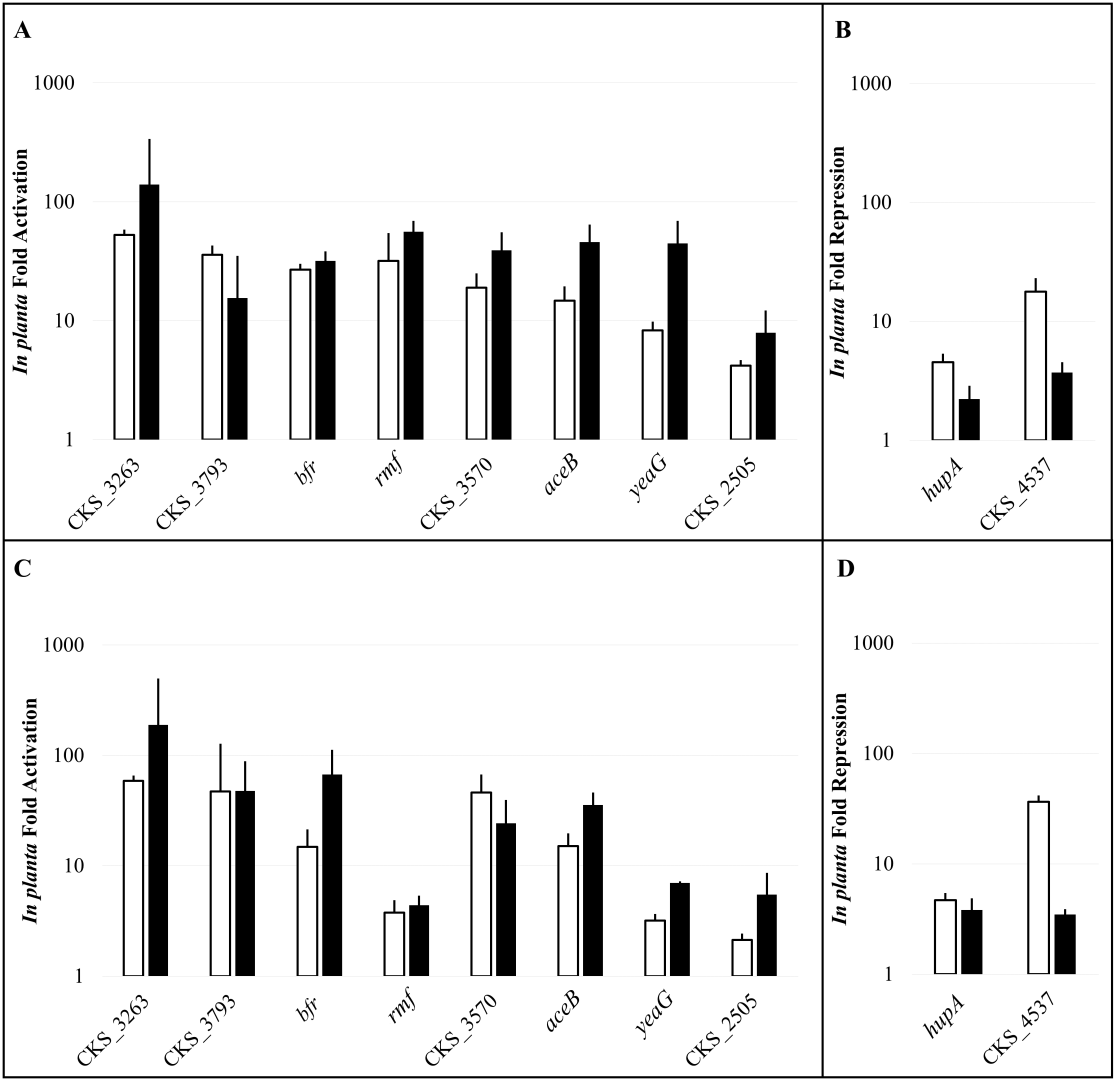
Relative gene expression from the RNA-Seq and qRT-PCR data. Changes in expression of ten select genes were compared between the RNA-Seq RPM analysis (white) and qRT-PCR analysis (black). Results are shown for the *in planta* culture data and the pre-inoculum *in vitro* liquid culture data (A and B) or plate *in vitro* culture data (C and D). The fold activation (A and C) or repression (B and D) for the *in planta* data is represented on a logarithmic scale. RNA-Seq results are averages of two experimental samples and qRT-PCR data represent two experimental samples analyzed in triplicate. For both RNA-Seq and qRT-PCR, the error bars were estimated using the sample standard error of the fold-change across the two independent biological replicates. The *recF* gene was used as the reference for normalization of the qRT-PCR results.

Although the absolute values for the RNA-Seq and qRT-PCR gene expression fold changes were not identical, the Pfaffl method for qRT-PCR analysis yielded similar trends for all genes chosen for expression validation from the RNA-Seq data (Fig. 2, Tables S7 and S8). Similar trends were found using all three of the housekeeping control genes *recF* (Fig. 2), *gyrB*, and *atpD* (Tables S7 and S8). Thus, all three of these genes have the capacity to serve as appropriate internal controls for future studies of the *P. stewartii* transcriptome. The overall RNA-Seq dataset in this study was strongly supported based upon the qRT-PCR analysis, enabling further bioinformatics analysis of the full data set, specifically with regard to regulation of biological processes *in planta*.

### Gene ontology (GO) analysis demonstrates the importance of select groups of genes *in planta*

**Figure 3:**
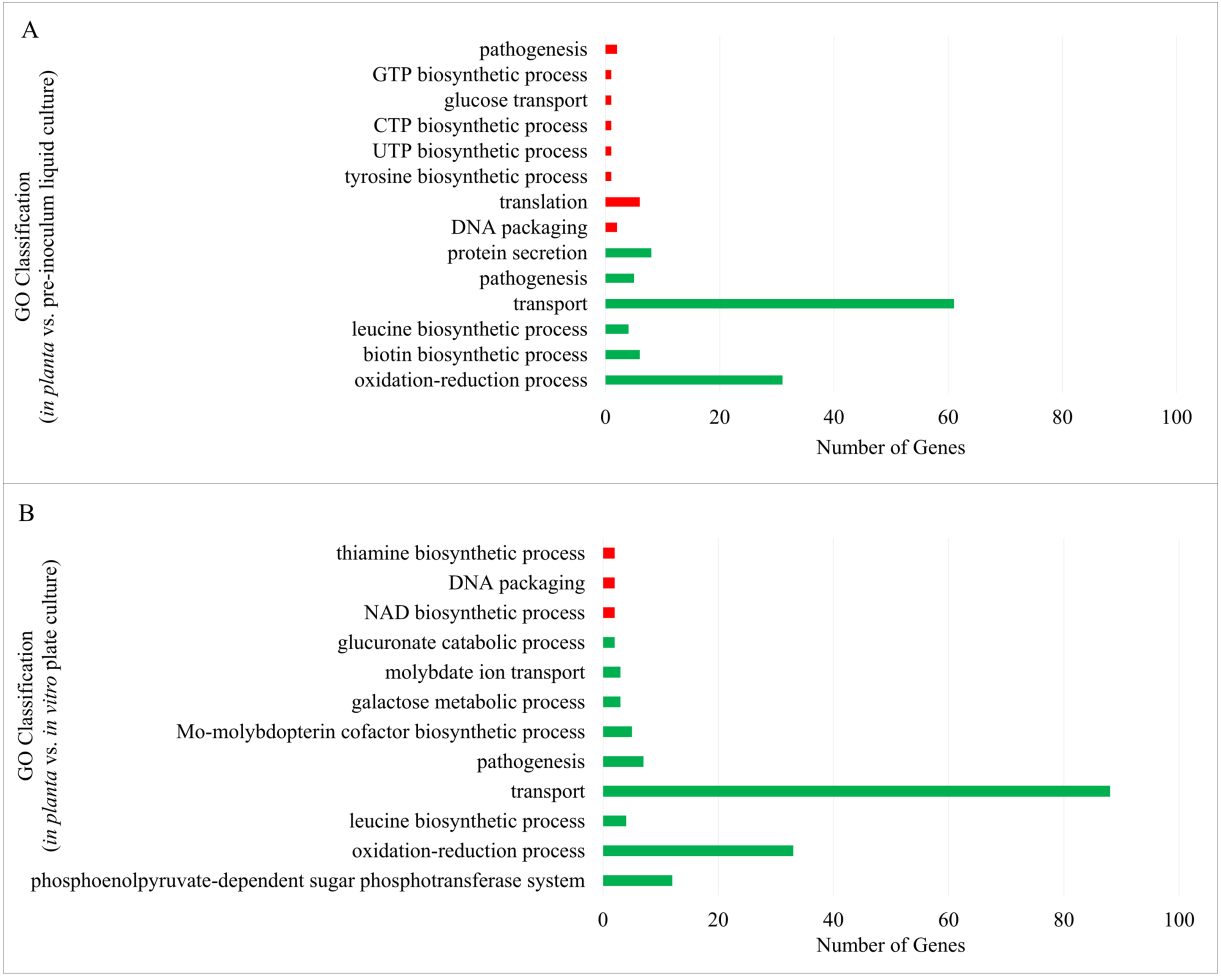
Gene Ontology analysis groupings for the list of regulated genes. Groups created from genes differentially expressed through the RNA-Seq RPM data comparisons between the *in planta* and pre-inoculum *in vitro* liquid culture (A) or *in vitro* plate culture (B). Upregulated (green) and downregulated (red) groups had a maximum *p*-value of 0.01 when using Fisher’s exact test in topGO.

GO analysis was used to identify common patterns in the functions of differentially expressed genes identified through RNA-Seq as described above. The most significant biological processes driving major physiological responses in *P. stewartii* during late stage biofilm formation in the plant are shown in Fig. 3. From the *in planta* vs. pre-inoculum liquid culture upregulated set of genes, GO analysis revealed six different groups of genes under the biological processes hierarchy with a *p*-value below 0.01 (Fig. 3A and Table S9). The groups with the highest number of genes were involved in transport (with 61 genes), followed by the oxidation–reduction process (with 31 genes), and protein secretion (with eight genes). For the downregulated set of genes, eight different groups were given from the biological processes hierarchy, and the group with the highest number of genes related to translation (with six genes) (Fig. 3A and Table S9).

From the *in planta* versus *in vitro* plate culture GO analysis nine biological process groups were identified with a *p*-value below 0.01 from the upregulated genes (Fig. 3B and Table S10). As with the *in planta* and pre-inoculum liquid culture comparison, transporters (with 88 genes) and oxidation–reduction processes (with 33 genes) were the top two GO categories represented, with phosphoenolpyruvate-dependent sugar phosphatase system genes third on the list (12 genes). The downregulated genes from this comparison resulted in three groups of biological processes. All three groups in the downregulated set had the same number of genes (with two genes) downregulated in their categories (Fig. 3B and Table S10). Thus genes associated with transporters and oxidation–reduction processes appear to play a vital role for *P. stewartii in planta.*

## Discussion

There are a number of phytopathogens that preferentially colonize the xylem of target plants. However, to date, knowledge about the full set of genes required for plant colonization and virulence is limited. Previous studies aimed at analyzing the transcriptome of bacterial vascular pathogens have been performed *in vitro* (Kimbrel et al., 2011). Microarray technology was successfully used to analyze the *in planta* transcriptome of *Ralstonia solanacearum* and *Xanthomonas oryzae* pv. *oryzae* (Soto-Suárez et al., 2010; Jacobs et al., 2012). Other work using dual-transcriptome RNA-Seq to simultaneously analyze the gene expression patterns of bacterial or fungal pathogens within their hosts has also been performed (Camilios-Neto et al., 2014). However, the challenge of performing transcriptome-level protocols on *in planta* bacterial samples remains a common deterrent due to difficulty extracting high quality bacterial mRNA in appropriate abundance. Here, RNA-Seq technology was used to analyze the full transcriptome of *P. stewartii* isolated as a monoculture grown within the xylem of a corn plant. A comparison of the *in planta* culture to either a pre-inoculum *in vitro* liquid culture or an *in vitro* agar plate culture revealed that ∼10% of the genome exhibited greater than four-fold changes in gene expression (Fig. 1). Many of these differentially expressed genes are likely required specifically during host infection. The RNA-Seq data was validated using qRT-PCR analysis of a select set of ten differentially expressed genes (Fig. 2). This enabled confidence in a bioinformatics GO analysis that revealed gene expression associated with the biological processes of transport and oxidation/reduction groups are significantly upregulated *in planta* suggesting that these genes play a critical role in plant colonization and/or virulence (Fig. 3).

In many plant-pathogen interactions, survival within the host depends upon the pathogen’s ability to adapt to its environment (Roper, 2011; Fatima & Senthil-Kumar, 2015). Bacteria are known to have the ability to influence host metabolism and take advantage of the resulting nutrient availability (Guo et al., 2012). Previous work has shown that *P. stewartii* is able to alter metabolic pathways within the host corn plant, specifically the phenylpropanoid pathway (Asselin et al., 2015). Disruption of this pathway could cause an alteration of metabolites available for the bacteria to utilize. In the *P. stewartii in planta* culture, by far, the largest group of genes upregulated four-fold or greater included genes encoding transporters for amino acids (e.g., alanine, arginine, aspartate, glutamate, histidine, isoleucine, leucine, lysine, valine), sugars (e.g., arabinose, galactose, ribose, xylose), and other compounds (e.g., ammonium, magnesium, molybdate, sulfate, taurine) in comparison to either of the *in vitro* culture conditions. Some transcriptional regulators associated with these transporters were also upregulated, including *nac,* involved in regulating genes associated with nitrogen assimilation and *araC,* important for arabinose catabolism. The observed regulatory patterns indicate the transporters are activated while in the host, suggesting availability or preference of the transporter-associated molecules within the nutrient-limited xylem. Published work by others has shown that ABC-transporters and TonB-dependent transporters are used by the bacteria to scavenge for plant derived carbohydrates in otherwise nutrient poor environments such as leaf surfaces, apoplast, and xylem niches (Blanvillain et al., 2007; Delmotte et al., 2009; Fatima & Senthil-Kumar, 2015). Whether specific nutrients are normally available in the xylem or specifically produced in response to the bacterial infection remains to be determined for *P. stewartii in planta*.

The second largest group of annotated genes upregulated *in planta* corresponded to genes associated with oxidation reduction biological processes. This list included cytochrome d ubiquinol subunits (CKS_3793 and *cytD* (CKS_3794)). In *E. coli*, these subunits are involved in electron transport only when oxygen is very limited in the environment (Miller & Gennis, 1983; Anraku & Gennis, 1987). This suggests the corn xylem and/or bacterial biofilm is an oxygen-limited environment for the bacteria, which supports previous conclusions from studies performed with other vascular pathogens (Pegg, 1985; Dalsing et al., 2015).

Annotated genes associated with fatty acid metabolism were also grouped into the oxidation reduction GO category. The *fadJ* gene (CKS_2016) encodes a protein primarily involved in anaerobic degradation of long and medium-chain fatty acids (Campbell, Morgan-Kiss & Cronan, 2003) and *fadE* (CKS_0306) is an acyl-coenzyme A dehydrogenase involved in the fatty-acid beta-oxidation (Campbell & Cronan, 2002). Most of the other genes involved in fatty acid metabolism were also upregulated *in planta* with the exception of *fadD, fadH* and *fadR*, the latter of which encodes a regulator for the pathway. Interestingly, the glyoxylate cycle associated genes *aceA* and *aceB* (CKS_4658 and CKS_4657), encoding isocitrate lyase and malate synthase respectively, and the latter of which was used for qRT-PCR validation, were also expressed *in planta*. These results indicate the potential availability of long-chain fatty acids as a carbon source for the bacteria while in the xylem, resulting in the use of beta-oxidation and the glyoxylate bypass pathways.

Annotated dehydrogenases involved in oxidation of a number of other potential carbon sources/metabolic intermediates, including aldehyde dehydrogenase (*aldB*), an altronate oxidoreductase involved in pentose-gluconate interconversion, succinate-semialdehyde dehydrogenase (*gabD*), glycerol dehydrogenase (*gldA*), myo-inositol dehydrogenase, and Zn-containing alcohol dehydrogenase, were also upregulated. The NAD(P) transhydrogenase alpha and beta subunits (encoded by *pntA* and *pntB*) involved in the transhydrogenation between NAD(H) and NADP(H) was an upregulated function as well. Collectively, these findings suggest that the overall physiology of *P. stewartii* is being altered *in planta* so that the cells have a greater capacity to utilize alternative carbon sources and/or alter internal carbon flow to their advantage, permitting successful growth in the nutrient-limited xylem during late stage infection where the impact of stationary phase also likely plays an important role.

Within the oxidation reduction processes GO category were also a few annotated genes upregulated that are associated with environmental stresses on the bacteria. This included genes important for the oxidative stress response, *sodC* (CKS_3446), which encodes a superoxide dismutase and CKS_3597, encoding catalase. Additionally, *hmp* (CKS_1509) encodes the nitric oxide dioxygenase, which converts nitric oxide to nitrate. Nitric oxide is known to be used by plants as a signaling molecule during defense (Torres, Jones & Dangl, 2006) and can also produce reactive nitrogen species when reacted with superoxide (Bellin et al., 2012). During plant defense response, many plants are known to secrete reactive oxygen species should be used instead of ROS as a way to combat pathogenic infection (O’Brien et al., 2012). The expression of these genes by *P. stewartii* could indicate a method of defense utilized by the phytopathogen against the plant immune response.

Virulence factors are also known to be important to successful plant infection by *P. stewartii.* It has long been known that one of the two *P. stewartii* T3SS is essential for the early stages of plant infection (Frederick et al., 2001; Roper, 2011) and the other is important for colonization of the corn flea beetle (Correa et al., 2012). The RNA-Seq studies have determined that genes encoding HrpA, HprB, HrpD, HrpF, HrpJ, HrpN, HrpO, and HrpT family proteins, as well as many other T3SS related genes, are highly expressed *in planta* (Tables S2 and S3). This supports previous work showing the importance of T3SS in pathogenesis (Frederick et al., 2001), but reveals their potential continued involvement in late-stage *P. stewartii* infection. Use of a T3SS throughout infection has been seen from an *in planta* analysis of *R. solanacearum* (Jacobs et al., 2012), but this has not been demonstrated experimentally in *P. stewartii* (Merighi et al., 2006; Roper, 2011).

In conclusion, very little is known about the expression of genes required during *in planta* infection within vascular pathogens. Transcriptomic work enables large-scale analysis of patterns of gene expression within the bacteria during their interaction with the host. Analysis of changes in the *P. stewartii in planta* transcriptome has revealed some of the key groups of genes, such as nutrient transporters and metabolic oxidation reduction processes including associated regulators, expressed by the bacteria during colonization and growth in the xylem. These findings may also apply to other xylem-dwelling and wilt disease-causing phytopathogens.

##  Supplemental Information

10.7717/peerj.3237/supp-1 Table S1 Strains and plasmids used in this study. ^a^ Nal = nalidixic acid resistance, Amp^*r*^ = ampicillin resistance

10.7717/peerj.3237/supp-2 Table S2 RNA-Seq data of differentially expressed genes between the *in planta* culture and the pre-inoculum *in vitro* liquid culture^a^. ^a^ A = activated in the *in planta* culture compared to the pre-inoculum liquid culture (lower in liquid culture), R = repressed in the *in planta* culture compared to the pre-inoculum liquid culture (higher in liquid culture).

10.7717/peerj.3237/supp-3 Table S3 RNA-Seq data of differentially expressed genes between the *in planta* culture and the *in vitro* plate culture^a^. ^a^ A = activated in the *in planta* culture compared to *in vitro* plate culture (lower in plate culture), R = repressed in the *in planta* culture compared to *in vitro* plate culture (higher in plate culture).

10.7717/peerj.3237/supp-4 Table S4 Primers designed for the genes of interest selected for cloning and qRT-PCR ^a^. ^a^ Primers listed as coding DNA sequence (CDS) were for the cloning of each gene into pGEM-T, and RT was for the qRT-PCR protocol.

10.7717/peerj.3237/supp-5 Table S5 RNA-Seq data of differentially expressed genes found in the *in planta* culture compared to both the pre-inoculum *in vitro* liquid culture and the *in vitro* plate culture^a^ ^a^ A = activated in the *in planta* culture compared to both the pre-inoculum *in vitro* liquid culture and the *in vitro* plate culture (lower in the liquid and plate cultures), R = repressed in the *in planta* culture compared to both the pre-inoculum *in vitro* liquid culture and the *in vitro* plate culture (higher in the liquid and plate cultures).

10.7717/peerj.3237/supp-6 Table S6 Additional genes with greater than four-fold regulation as calculated through the DESeq analysis ^a^ R = repressed in the *in planta* culture compared to either the pre-inoculum *in vitro* liquid culture or the *in vitro* plate culture (higher in the liquid or plate cultures).

10.7717/peerj.3237/supp-7 Table S7 Results for qRT-PCR validation for the *in planta* culture and the pre-inoculum *in vitro* liquid culture comparison Genes upregulated (activated) *in planta*, with the exception of those designated * which were downregulated (repressed) *in planta*.

10.7717/peerj.3237/supp-8 Table S8 Results for qRT-PCR validation for the *in planta* culture and the *in vitro* plate culture comparison Genes upregulated (activated) *in planta*, with the exception of those designated * which were downregulated (repressed) *in planta*.

10.7717/peerj.3237/supp-9 Table S9 GO gene groups from four-fold regulated genes in the *in planta* culture compared to the pre-inoculum *in vitro* liquid culture

10.7717/peerj.3237/supp-10 Table S10 GO gene groups from four-fold regulated genes in the *in planta* culture compared to the *in vitro* plate culture

## Additional Information and Declarations

### Data Availability

The following information was supplied regarding data availability: For accessing fastq read files (on the NCBI Sequence Read Archive (SRA) Database) and excel files (on NCBI Gene Expression Omnibus (GEO) Database), please use the accession numbers provided in the text.
https://www.ncbi.nlm.nih.gov/geo/query/acc.cgi?token=slefewsojdyrxwpacc=GSE87520.
