# Supplementary material for: Analysis of the in planta transcriptome expressed by the corn pathogen Pantoea stewartii subsp. stewartii via RNA-Seq
Source: PeerJ. 2017 Apr 27;5:e3237. doi: 10.7717/peerj.3237 (PMC5410145; doi:10.7717/peerj.3237)
Supplement: Table S1 — a Nal = nalidixic acid resistance, Ampr = ampicillin resistance [file peerj-05-3237-s001.docx]

**Table S1.** Strains and plasmids used in this study

| **Strain/ Plasmid** | **Description^a^** |
| --- | --- |
| *E. coli* Top10 | Competent strain |
| *P. stewartii* DC283 | Wild-type strain, Nal^r^ |
| p-GEM-T | Cloning vector, Amp^r^ |
| p-GEM-T+ CKS_3263 | CKS_3263 coding region for cloning inserted into p-GEM-T vector |
| p-GEM-T+ CKS_3793 | CKS_3793 coding region for cloning inserted into p-GEM-T vector (Kernell Burke et al., 2015) |
| p-GEM-T+ *rmf* | *rmf* coding region for cloning inserted into p-GEM-T vector |
| p-GEM-T+ *bfr* | *bfr* coding region for cloning inserted into p-GEM-T vector |
| p-GEM-T+ CKS_3570 | CKS_3570 coding region for cloning inserted into p-GEM-T vector |
| p-GEM-T+ *aceB* | *aceB* coding region for cloning inserted into p-GEM-T vector |
| p-GEM-T+ *yeaG* | *yeaG* coding region for cloning inserted into p-GEM-T vector |
| p-GEM-T+ CKS_2505 | CKS_2505 coding region for cloning inserted into p-GEM-T vector |
| p-GEM-T+ *hupA* | *hupA* coding region for cloning inserted into p-GEM-T vector |
| p-GEM-T+ CKS_4537 | CKS_4537 coding region for cloning inserted into p-GEM-T vector |
| p-GEM-T+ *recF* | *recF* coding region for cloning inserted into p-GEM-T vector |
| p-GEM-T+ *atpD* | *atpD* coding region for cloning inserted into p-GEM-T vector |
| p-GEM-T+ *gyrB* | *gyrB* coding region for cloning inserted into p-GEM-T vector |

**^a^**Nal^r^ = nalidixic acid resistance, Amp^r^ = ampicillin resistance
