# Supplementary material for: Analysis of the in planta transcriptome expressed by the corn pathogen Pantoea stewartii subsp. stewartii via RNA-Seq
Source: PeerJ. 2017 Apr 27;5:e3237. doi: 10.7717/peerj.3237 (PMC5410145; doi:10.7717/peerj.3237)
Supplement: Table S2 — a A = activated in the in planta culture compared to the pre-inoculum liquid culture (lower in liquid culture), R = repressed in the in planta culture compared to the pre-inoculum liquid culture (higher in liquid culture). [file peerj-05-3237-s002.docx]

**Table S2.** RNA-Seq data of differentially expressed genes between the *in planta* culture and the pre-inoculum *in vitro* liquid culture^a^

| **Locus Tag** | **Annotation** | | **RPM Fold Regulation** | **DESeq Fold Regulation** | **DESeq padj** |
| --- | --- | --- | --- | --- | --- |
| CKS_4725 | hypothetical protein | A | 65.18 | 32.69 | 3.68E-21 |
| CKS_3692 | hypothetical protein | A | 56.09 | 30.15 | 6.34E-24 |
| CKS_3263 | HrpA family pilus protein | A | 52.64 | 34.17 | 1.09E-85 |
| CKS_5750 | acid shock protein | A | 49.49 | 10.42 | 2.36E-04 |
| CKS_2320 | putative alkanal monooxygenase | A | 38.39 | 22.22 | 4.30E-24 |
| CKS_3793 | cytochrome d ubiquinol oxidase subunit I | A | 36.48 | 23.81 | 2.78E-59 |
| CKS_3039 | hypothetical protein | A | 35.53 | 22.76 | 4.44E-39 |
| CKS_3355 | periplasmic-binding component of an ABC superfamily ribose transporter | A | 33.06 | 20.26 | 1.17E-26 |
| CKS_2883 | predicted dethiobiotin synthetase | A | 31.33 | 19.41 | 8.43E-28 |
| CKS_3407 | phosphoenolpyruvate synthase | A | 30.76 | 19.58 | 2.67E-36 |
| CKS_2379 | nudix hydrolase | A | 30.68 | 18.88 | 4.68E-27 |
| CKS_3537 | periplasmic-binding component of an ABC superfamily L-arabinose transporter | A | 29.41 | 19.22 | 2.16E-56 |
| CKS_4032 | ribosome modulation factor | A | 28.23 | 17.29 | 3.37E-21 |
| CKS_3794 | cytochrome d terminal oxidase subunit II | A | 28.18 | 18.52 | 9.78E-51 |
| CKS_1610 | ATP-binding component of an ABC superfamily taurine transporter | A | 27.50 | 12.87 | 2.66E-08 |
| CKS_1591 | bacterioferritin iron storage and detoxification protein | A | 27.19 | 17.89 | 1.27E-50 |
| CKS_2202 | hypothetical protein | A | 27.12 | 16.70 | 5.90E-24 |
| CKS_2922 | fasciclin-like repeat-containing secreted/surface protein | A | 26.37 | 17.22 | 4.33E-40 |
| CKS_3795 | membrane-bound protein | A | 25.59 | 16.89 | 1.32E-45 |
| CKS_3356 | hypothetical protein | A | 24.51 | 14.80 | 4.89E-18 |
| CKS_2442 | aldehyde dehydrogenase B | A | 23.70 | 15.58 | 2.52E-37 |
| CKS_4281 | hypothetical protein | A | 23.51 | 13.17 | 9.43E-13 |
| CKS_0668 | predicted quinol oxidase subunit | A | 22.60 | 13.69 | 8.70E-19 |
| CKS_2380 | hypothetical protein | A | 22.17 | 13.82 | 4.61E-22 |
| CKS_3948 | predicted inner membrane protein | A | 21.90 | 14.35 | 1.16E-33 |
| CKS_0801 | ATP-binding component of an ABC superfamily predicted amino-acid transporter | A | 21.75 | 14.24 | 2.44E-30 |
| CKS_0368 | L-lactate dehydrogenase FMN-linked | A | 21.46 | 11.94 | 1.52E-10 |
| CKS_0515 | predicted dioxygenase | A | 20.57 | 12.70 | 1.38E-21 |
| CKS_1609 | membrane component of an ABC superfamily taurine transporter | A | 20.25 | 10.23 | 9.28E-08 |
| CKS_2375 | hypothetical protein | A | 20.00 | 11.57 | 3.76E-12 |
| CKS_3270 | HrpF family protein | A | 19.63 | 13.29 | 1.80E-62 |
| CKS_3570 | AraC family transcriptional regulator | A | 19.33 | 12.66 | 2.19E-33 |
| CKS_3281 | HopAM1-1 family type III effector | A | 19.28 | 12.43 | 9.41E-29 |
| CKS_3513 | oxidoreductase domain protein | A | 18.47 | 12.03 | 4.38E-32 |
| CKS_2985 | hypothetical protein | A | 18.01 | 10.46 | 2.81E-11 |
| CKS_3320 | GGDEF domain protein | A | 17.30 | 11.00 | 1.08E-16 |
| CKS_1103 | stress-induced protein | A | 16.70 | 10.85 | 4.87E-20 |
| CKS_4575 | secretion system chaperone | A | 16.57 | 10.59 | 5.67E-24 |
| CKS_3796 | YbgE family protein | A | 15.82 | 10.59 | 8.32E-34 |
| CKS_4955 | permease component of an ABC superfamily transporter | A | 15.79 | 9.96 | 1.37E-17 |
| CKS_4830 | Amidohydrolase | A | 15.67 | 10.21 | 8.43E-28 |
| CKS_1508 | hypothetical protein | A | 15.52 | 10.12 | 1.41E-22 |
| CKS_2384 | hypothetical protein | A | 15.52 | 9.17 | 6.35E-10 |
| CKS_3767 | hypothetical protein | A | 15.27 | 9.96 | 8.32E-23 |
| CKS_4657 | malate synthase A | A | 15.20 | 9.76 | 2.40E-20 |
| CKS_3265 | HrpB family protein | A | 14.99 | 9.94 | 3.73E-28 |
| CKS_2186 | membrane component of an ABC superfamily methyl-galactoside transporter | A | 14.99 | 10.08 | 1.48E-36 |
| CKS_0687 | predicted pirin-related protein | A | 14.88 | 9.14 | 1.07E-13 |
| CKS_3512 | oxidoreductase domain protein | A | 14.69 | 9.79 | 2.18E-27 |
| CKS_3503 | methyl-accepting chemotaxis protein | A | 14.60 | 9.70 | 1.03E-23 |
| CKS_4279 | hypothetical protein | A | 14.60 | 9.19 | 3.75E-15 |
| CKS_2376 | hypothetical protein | A | 14.52 | 9.23 | 6.34E-15 |
| CKS_3267 | type III secretion system lipoprotein | A | 14.32 | 9.45 | 1.90E-26 |
| CKS_0560 | hypothetical protein | A | 14.07 | 9.65 | 3.62E-55 |
| CKS_3851 | 8-amino-7-oxononanoate synthase | A | 13.99 | 9.32 | 2.23E-28 |
| CKS_5439 | hypothetical protein | A | 13.72 | 8.61 | 1.52E-15 |
| CKS_3275 | HrpN family hypersensitivity reaction elicitor | A | 13.62 | 9.05 | 3.83E-24 |
| CKS_4573 | hypothetical protein | A | 13.54 | 8.90 | 7.83E-25 |
| CKS_2772 | anthranilate phosphoribosyltransferase | A | 13.32 | 8.72 | 3.88E-24 |
| CKS_2488 | ATP-binding component of an ABC superfamily sugar transporter | A | 13.29 | 8.95 | 1.28E-30 |
| CKS_3579 | putative MFS superfamily benzoate transporter | A | 13.24 | 8.98 | 1.27E-37 |
| CKS_1611 | periplasmic-binding component of an ABC superfamily taurine transporter | A | 13.12 | 4.67 | 1.79E-02 |
| CKS_4574 | putative type III secretion system effector protein | A | 12.95 | 8.59 | 1.42E-27 |
| CKS_0908 | Pirin | A | 12.78 | 8.25 | 3.58E-18 |
| CKS_4719 | conserved inner membrane protein involved in acetate transport | A | 12.70 | 7.88 | 7.29E-11 |
| CKS_2366 | hypothetical protein | A | 12.63 | 7.83 | 6.07E-10 |
| CKS_2378 | hypothetical protein | A | 12.38 | 8.05 | 3.25E-16 |
| CKS_0370 | hypothetical protein | A | 12.16 | 8.22 | 3.08E-32 |
| CKS_0371 | hypothetical protein | A | 12.05 | 8.24 | 1.14E-41 |
| CKS_3850 | biotin synthase | A | 11.91 | 7.96 | 9.41E-29 |
| CKS_2371 | hypothetical protein | A | 11.84 | 7.61 | 6.83E-14 |
| CKS_3319 | predicted inner membrane protein | A | 11.65 | 7.58 | 8.54E-13 |
| CKS_2373 | hypothetical protein | A | 11.65 | 7.53 | 2.41E-13 |
| CKS_2372 | putative transcriptional regulator | A | 11.54 | 7.42 | 7.14E-13 |
| CKS_4954 | Aryldialkylphosphatase | A | 11.52 | 7.64 | 7.24E-20 |
| CKS_0204 | ammonium transporter | A | 11.47 | 4.16 | 3.00E-02 |
| CKS_2504 | gamma-aminobutyrate:alpha-ketoglutarate aminotransferase | A | 11.42 | 7.63 | 1.32E-19 |
| CKS_3539 | L-arabinose transport system permease protein | A | 11.35 | 7.45 | 1.33E-16 |
| CKS_0380 | hypothetical protein | A | 11.33 | 6.65 | 7.39E-07 |
| CKS_2984 | hypothetical protein | A | 11.32 | 7.17 | 2.23E-12 |
| CKS_4282 | hypothetical protein | A | 11.32 | 7.35 | 1.61E-15 |
| CKS_4280 | hypothetical protein | A | 11.29 | 7.67 | 8.75E-34 |
| CKS_3715 | hypothetical protein | A | 11.24 | 7.31 | 5.57E-12 |
| CKS_4585 | putative type III secretion system apparatus protein | A | 11.21 | 7.66 | 2.11E-40 |
| CKS_3538 | ATP-binding component of an ABC superfamily L-arabinose transporter | A | 11.16 | 7.55 | 8.94E-27 |
| CKS_1361 | PLP-binding diaminopimelate decarboxylase | A | 11.05 | 7.23 | 1.52E-13 |
| CKS_1282 | sulfate adenylyltransferase subunit 1 | A | 11.03 | 6.86 | 8.16E-11 |
| CKS_4249 | putative membrane protein | A | 10.97 | 6.97 | 3.08E-12 |
| CKS_0390 | hypothetical protein | A | 10.95 | 7.25 | 9.20E-16 |
| CKS_2383 | hypothetical protein | A | 10.95 | 6.75 | 6.83E-09 |
| CKS_5150 | 3-isopropylmalate isomerase subunit dehydratase component | A | 10.85 | 7.27 | 6.58E-21 |
| CKS_0948 | mannonate hydrolase | A | 10.84 | 7.12 | 3.80E-15 |
| CKS_0963 | membrane component of an ABC superfamily D-ala-D-ala transporter | A | 10.71 | 7.16 | 1.15E-23 |
| CKS_4658 | isocitrate lyase | A | 10.56 | 6.78 | 1.11E-11 |
| CKS_2855 | pyridine nucleotide transhydrogenase beta subunit | A | 10.52 | 6.81 | 6.90E-11 |
| CKS_2854 | pyridine nucleotide transhydrogenase alpha subunit | A | 10.46 | 6.81 | 4.87E-12 |
| CKS_4572 | hypothetical protein | A | 10.45 | 6.69 | 1.37E-11 |
| CKS_2773 | component I of anthranilate synthase | A | 10.21 | 6.76 | 1.15E-19 |
| CKS_4576 | putative type III secretion system effector protein | A | 10.12 | 6.89 | 2.44E-32 |
| CKS_1281 | adenosine 5'-phosphosulfate kinase | A | 10.10 | 6.42 | 6.74E-11 |
| CKS_4283 | hypothetical protein | A | 10.09 | 6.63 | 1.61E-13 |
| CKS_2381 | hypothetical protein | A | 10.07 | 6.74 | 1.19E-19 |
| CKS_2679 | mannose-specific enzyme IID component of PTS | A | 9.99 | 6.67 | 1.64E-15 |
| CKS_4634 | mutator family transposase | A | 9.90 | 6.43 | 8.37E-13 |
| CKS_3535 | L-arabinose isomerase | A | 9.86 | 6.53 | 5.70E-17 |
| CKS_4586 | secretion system apparatus protein | A | 9.82 | 6.73 | 2.84E-41 |
| CKS_4953 | hypothetical protein | A | 9.78 | 6.45 | 2.05E-15 |
| CKS_4019 | alkanesulfonate monooxygenase FMNH(2)-dependent | A | 9.66 | 5.89 | 3.45E-07 |
| CKS_0367 | DNA-binding transcriptional repressor | A | 9.58 | 5.65 | 4.71E-05 |
| CKS_2680 | mannose-specific enzyme IIC component of PTS | A | 9.47 | 6.26 | 1.01E-12 |
| CKS_3254 | HrpO family protein | A | 9.41 | 6.04 | 5.24E-10 |
| CKS_1762 | drug/metabolite transporter (DMT) superfamily permease | A | 9.37 | 6.12 | 1.67E-13 |
| CKS_3623 | hypothetical protein | A | 9.35 | 6.38 | 5.97E-29 |
| CKS_4829 | integral membrane protein | A | 9.31 | 5.85 | 3.52E-09 |
| CKS_2321 | putative cytoplasmic protein | A | 9.30 | 5.45 | 1.95E-05 |
| CKS_5511 | hypothetical protein | A | 9.29 | 5.42 | 9.29E-06 |
| CKS_1541 | IMP dehydrogenase | A | 9.27 | 6.30 | 3.56E-25 |
| CKS_0962 | periplasmic-binding component of an ABC superfamily D-ala-D-a la transporter | A | 9.26 | 6.34 | 1.61E-31 |
| CKS_3354 | short-chain dehydrogenase/reductase | A | 9.22 | 6.13 | 2.40E-13 |
| CKS_3597 | Catalase | A | 9.20 | 5.96 | 6.37E-13 |
| CKS_4597 | type III secretion system inner membrane protein | A | 9.20 | 5.93 | 1.12E-10 |
| CKS_3619 | hypothetical protein | A | 9.17 | 6.21 | 1.21E-18 |
| CKS_2495 | FAD dependent oxidoreductase | A | 9.13 | 5.89 | 3.29E-10 |
| CKS_4596 | hypothetical protein | A | 9.09 | 6.10 | 4.87E-20 |
| CKS_2489 | permease component of an ABC superfamily ribose/xylose/arabinose/galactoside transporter | A | 9.08 | 6.14 | 3.49E-18 |
| CKS_1283 | sulfate adenylyltransferase subunit 2 | A | 9.08 | 5.60 | 1.54E-06 |
| CKS_5454 | putative cell wall-associated hydrolase | A | 9.07 | 6.16 | 3.11E-19 |
| CKS_3982 | predicted transporter | A | 9.06 | 6.02 | 2.82E-19 |
| CKS_4701 | hypothetical protein | A | 9.04 | 5.90 | 4.93E-12 |
| CKS_2765 | hypothetical protein | A | 8.99 | 6.06 | 5.43E-20 |
| CKS_3268 | HrpD family protein | A | 8.97 | 5.93 | 2.69E-13 |
| CKS_4331 | PTS system cellobiose-specific IIB component | A | 8.90 | 5.75 | 5.40E-09 |
| CKS_2016 | enoyl-CoA hydratase/3-hydroxyacyl-CoA dehydrogenase | A | 8.89 | 5.97 | 2.11E-13 |
| CKS_2806 | putative formate dehydrogenase oxidoreductase protein | A | 8.84 | 5.29 | 2.87E-05 |
| CKS_4653 | periplasmic binding protein | A | 8.83 | 5.88 | 2.77E-19 |
| CKS_4923 | anaerobic ribonucleoside-triphosphate reductase | A | 8.78 | 5.97 | 1.16E-29 |
| CKS_4463 | sn-glycerol-3-phosphate transporter | A | 8.76 | 5.77 | 6.04E-10 |
| CKS_4583 | secretion system apparatus protein | A | 8.69 | 5.91 | 4.88E-28 |
| CKS_0940 | myo-inositol 2-dehydrogenase | A | 8.68 | 5.73 | 3.28E-12 |
| CKS_4009 | uncharacterized DUF882 family protein | A | 8.65 | 5.94 | 5.86E-27 |
| CKS_2222 | predicted peptidase | A | 8.62 | 5.31 | 2.29E-07 |
| CKS_2800 | L-ribulose-5-phosphate 4-epimerase | A | 8.51 | 5.74 | 1.58E-16 |
| CKS_2792 | aconitate hydratase 1 | A | 8.45 | 5.73 | 1.16E-22 |
| CKS_4024 | dihydro-orotate oxidase FMN-linked | A | 8.43 | 5.64 | 4.20E-15 |
| CKS_2150 | hypothetical protein | A | 8.42 | 5.63 | 1.85E-17 |
| CKS_3201 | membrane component of an ABC superfamily polar amino acid transporter | A | 8.37 | 5.60 | 2.31E-12 |
| CKS_2368 | gp55 family protein | A | 8.35 | 5.53 | 5.56E-11 |
| CKS_0451 | hypothetical protein | A | 8.27 | 5.36 | 2.79E-10 |
| CKS_2385 | hypothetical protein | A | 8.27 | 5.48 | 2.65E-11 |
| CKS_4509 | hypothetical protein | A | 8.25 | 5.54 | 3.66E-12 |
| CKS_2714 | serine-protein kinase | A | 8.22 | 5.66 | 1.38E-29 |
| CKS_3258 | HrcJ family type III secretion system component protein | A | 8.21 | 5.60 | 5.17E-23 |
| CKS_0452 | putative peptidase | A | 8.19 | 5.57 | 1.41E-21 |
| CKS_2972 | hypothetical protein | A | 8.19 | 5.44 | 2.51E-12 |
| CKS_4763 | 23-diketo-L-gulonate dehydrogenase NADH-dependent | A | 8.17 | 5.29 | 2.45E-10 |
| CKS_3703 | L-fucose operon activator | A | 8.13 | 4.99 | 3.78E-05 |
| CKS_4588 | type III secretion system apparatus protein | A | 8.13 | 5.48 | 5.17E-16 |
| CKS_3984 | pyruvate formate-lyase | A | 8.11 | 5.53 | 1.15E-18 |
| CKS_2370 | Heptosyltransferase | A | 8.09 | 5.25 | 1.25E-08 |
| CKS_4579 | hypothetical protein | A | 8.08 | 5.40 | 7.10E-16 |
| CKS_4767 | 3-keto-L-gulonate 6-phosphate decarboxylase | A | 8.02 | 5.05 | 8.75E-07 |
| CKS_0306 | medium-long-chain fatty acyl-CoA dehydrogenase | A | 8.01 | 5.39 | 9.63E-13 |
| CKS_3858 | molybdopterin biosynthesis protein A | A | 8.01 | 5.48 | 3.82E-23 |
| CKS_2367 | Chitinase | A | 8.00 | 5.41 | 7.63E-15 |
| CKS_0961 | D-ala-D-ala dipeptidase Zn-dependent | A | 7.99 | 5.44 | 3.81E-17 |
| CKS_4272 | putative DNA modification methylase | A | 7.97 | 5.38 | 6.19E-16 |
| CKS_2185 | ATP-binding component of an ABC superfamily galactose/methyl galactoside transporter | A | 7.93 | 5.36 | 2.98E-12 |
| CKS_0468 | hypothetical protein | A | 7.91 | 5.35 | 2.78E-15 |
| CKS_5152 | 2-isopropylmalate synthase | A | 7.89 | 5.43 | 1.25E-28 |
| CKS_3446 | superoxide dismutase Cu Zn | A | 7.84 | 5.37 | 9.71E-18 |
| CKS_0266 | cytosine/purine/uracil/thiamine/allantoin permease family protein | A | 7.83 | 4.16 | 4.59E-03 |
| CKS_5467 | OspG family protein | A | 7.80 | 5.10 | 9.66E-09 |
| CKS_0964 | membrane component of an ABC superfamily D-ala-D-ala transporter | A | 7.76 | 5.13 | 7.66E-13 |
| CKS_2106 | hypothetical protein | A | 7.75 | 4.92 | 1.85E-05 |
| CKS_3278 | methyl-accepting chemotaxis sensory transducer | A | 7.67 | 5.21 | 1.82E-19 |
| CKS_3038 | iron-uptake factor | A | 7.66 | 5.17 | 1.31E-12 |
| CKS_3837 | galactose-1-epimerase (mutarotase) | A | 7.65 | 5.20 | 2.68E-18 |
| CKS_3739 | membrane component of an ABC superfamily glutamate and aspartate transporter | A | 7.62 | 5.21 | 2.63E-16 |
| CKS_2472 | hypothetical protein | A | 7.62 | 5.23 | 1.85E-17 |
| CKS_3569 | RND multidrug efflux membrane fusion protein | A | 7.61 | 5.03 | 8.62E-11 |
| CKS_4584 | putative type III secretion system apparatus protein | A | 7.59 | 5.19 | 5.90E-24 |
| CKS_1750 | N-acetylmuramoyl-L-alanine amidase | A | 7.58 | 5.00 | 1.02E-08 |
| CKS_3852 | malonyl-CoA methyltransferase | A | 7.57 | 5.03 | 4.35E-11 |
| CKS_2563 | putative mannosyl-3-phosphoglycerate phosphatase | A | 7.53 | 5.07 | 1.42E-13 |
| CKS_2490 | DNA-binding transcriptional dual regulator of nitrogen assimilation | A | 7.52 | 4.36 | 9.02E-04 |
| CKS_4021 | NAD(P)H-dependent FMN reductase | A | 7.50 | 4.29 | 1.22E-03 |
| CKS_0288 | putative permease component of an ABC superfamily amino acid transporter | A | 7.50 | 4.31 | 9.38E-04 |
| CKS_1697 | divalent cation transport protein | A | 7.49 | 5.15 | 1.09E-25 |
| CKS_5453 | hypothetical protein | A | 7.48 | 5.00 | 2.57E-09 |
| CKS_0429 | hypothetical protein | A | 7.47 | 5.07 | 3.28E-18 |
| CKS_4333 | beta-glucosidase | A | 7.43 | 5.01 | 6.13E-14 |
| CKS_2635 | hypothetical protein | A | 7.37 | 4.59 | 9.39E-06 |
| CKS_2586 | putative adenine methylase | A | 7.35 | 4.25 | 4.83E-04 |
| CKS_3094 | GAF domain/GGDEF domain/EAL domain protein | A | 7.34 | 4.65 | 5.52E-07 |
| CKS_3527 | stress-induced protein | A | 7.31 | 4.94 | 1.05E-16 |
| CKS_4761 | periplasmic substrate-binding component of an ABC superfamily ribose transporter | A | 7.30 | 4.96 | 6.63E-21 |
| CKS_3738 | permease component of an ABC superfamily glutamate/aspartate transporter | A | 7.29 | 4.96 | 9.17E-20 |
| CKS_3534 | membrane-fusion protein | A | 7.27 | 4.55 | 5.69E-06 |
| CKS_3859 | molybdopterin biosynthesis protein B | A | 7.26 | 4.92 | 3.78E-14 |
| CKS_0290 | putative ATP-binding component of an ABC superfamily amino acid transporter | A | 7.26 | 4.30 | 3.38E-04 |
| CKS_4952 | acetylornithine deacetylase | A | 7.23 | 4.80 | 4.37E-11 |
| CKS_4273 | hypothetical protein | A | 7.21 | 4.77 | 4.08E-10 |
| CKS_2803 | hypothetical protein | A | 7.20 | 4.91 | 2.24E-15 |
| CKS_4654 | hypothetical protein | A | 7.19 | 4.84 | 2.37E-17 |
| CKS_3146 | dihydrodipicolinate synthase | A | 7.18 | 4.71 | 3.98E-08 |
| CKS_1233 | protein disaggregation chaperone | A | 7.17 | 4.57 | 5.59E-07 |
| CKS_1882 | hypothetical protein | A | 7.16 | 4.85 | 1.33E-15 |
| CKS_2441 | Methyltransferase | A | 7.15 | 4.59 | 3.46E-07 |
| CKS_2493 | aldehyde dehydrogenase | A | 7.14 | 4.61 | 3.24E-07 |
| CKS_3607 | tail fiber assembly protein | A | 7.03 | 4.72 | 1.13E-10 |
| CKS_2924 | metal-activated pyridoxal enzyme | A | 7.02 | 4.72 | 2.70E-11 |
| CKS_2808 | hypothetical protein | A | 6.97 | 4.68 | 6.71E-13 |
| CKS_2377 | hypothetical protein | A | 6.96 | 4.74 | 3.08E-13 |
| CKS_1834 | glycerol dehydrogenase | A | 6.95 | 4.77 | 1.01E-19 |
| CKS_2713 | uncharacterized DUF444 family protein | A | 6.93 | 4.78 | 1.33E-20 |
| CKS_2492 | succinate-semialdehyde dehydrogenase I NADP-dependent | A | 6.92 | 4.67 | 1.02E-11 |
| CKS_2771 | indole-3-glycerol-phosphate synthase/anthranilate phosphoribosyltransferase | A | 6.91 | 4.69 | 1.51E-18 |
| CKS_3460 | hypothetical protein | A | 6.90 | 4.60 | 1.05E-07 |
| CKS_2301 | gamma-Glu-putrescine synthase | A | 6.90 | 4.74 | 9.14E-16 |
| CKS_2205 | DEAD/DEAH box helicase domain protein | A | 6.84 | 4.26 | 7.01E-05 |
| CKS_2364 | hypothetical protein | A | 6.81 | 4.66 | 1.32E-12 |
| CKS_2487 | putative periplasmic binding protein | A | 6.80 | 4.57 | 5.74E-09 |
| CKS_4915 | putative collagenase-like peptidase | A | 6.78 | 4.57 | 7.98E-14 |
| CKS_2525 | permease component of an ABC superfamily ribose transporter | A | 6.77 | 4.43 | 2.29E-07 |
| CKS_4056 | WrbA family flavoprotein | A | 6.71 | 4.63 | 2.85E-21 |
| CKS_0338 | glycerol dehydrogenase NAD | A | 6.66 | 4.51 | 8.57E-10 |
| CKS_0912 | hypothetical protein | A | 6.60 | 4.44 | 1.13E-10 |
| CKS_5151 | 3-isopropylmalate dehydrogenase | A | 6.59 | 4.54 | 1.81E-19 |
| CKS_2642 | hypothetical protein | A | 6.57 | 4.40 | 3.60E-10 |
| CKS_3276 | putative avirulence protein | A | 6.56 | 4.50 | 3.73E-19 |
| CKS_4940 | aspartate carbamoyltransferase catalytic subunit | A | 6.52 | 4.48 | 3.07E-24 |
| CKS_2983 | phytochelatin synthase | A | 6.41 | 4.37 | 1.86E-16 |
| CKS_2062 | permease component of an ABC superfamily spermidine/putrescine transporter | A | 6.36 | 4.32 | 1.49E-14 |
| CKS_4020 | periplasmic-binding component of an ABC superfamily alkanesulfonate transporter | A | 6.35 | 3.86 | 8.08E-04 |
| CKS_4958 | putative binding periplasmic protein of ABC transporter | A | 6.34 | 4.34 | 1.67E-12 |
| CKS_2386 | hypothetical protein | A | 6.34 | 4.26 | 1.15E-08 |
| CKS_3621 | aldo-keto reductase | A | 6.32 | 4.26 | 7.76E-10 |
| CKS_0379 | hypothetical protein | A | 6.30 | 4.18 | 4.91E-07 |
| CKS_3519 | putative formate dehydrogenase oxidoreductase protein | A | 6.27 | 4.28 | 1.42E-15 |
| CKS_2413 | uncharacterized DUF883 family protein | A | 6.22 | 4.08 | 5.38E-06 |
| CKS_0657 | altronate hydrolase | A | 6.21 | 4.23 | 7.13E-13 |
| CKS_4593 | hypothetical protein | A | 6.20 | 4.24 | 3.24E-16 |
| CKS_1037 | predicted transporter | A | 6.19 | 4.27 | 4.13E-15 |
| CKS_1859 | hypothetical protein | A | 6.18 | 4.20 | 3.42E-11 |
| CKS_4960 | hypothetical protein | A | 6.17 | 4.25 | 2.30E-18 |
| CKS_3620 | hypothetical protein | A | 6.17 | 4.18 | 5.42E-10 |
| CKS_2012 | long-chain fatty acid outer membrane transporter | A | 6.16 | 4.24 | 1.03E-15 |
| CKS_4762 | Gluconolactonase | A | 6.13 | 4.21 | 3.42E-18 |
| CKS_3702 | periplasmic substrate binding component of an ABC superfamily sugar transporter | A | 6.12 | 3.93 | 5.66E-05 |
| CKS_1706 | phosphoribosylaminoimidazole synthetase | A | 6.12 | 4.22 | 1.56E-14 |
| CKS_3489 | inner membrane iron-sulfur protein in SoxR-reducing complex | A | 6.12 | 4.21 | 2.64E-20 |
| CKS_4340 | heat shock chaperone | A | 6.04 | 3.82 | 9.88E-05 |
| CKS_0291 | serine--pyruvate aminotransferase / L-alanine:glyoxylate aminotransferase | A | 6.03 | 3.78 | 1.31E-04 |
| CKS_5149 | 3-isopropylmalate isomerase subunit | A | 6.02 | 4.12 | 2.87E-12 |
| CKS_3271 | type III secretion system outermembrane pore forming protein | A | 5.99 | 4.09 | 3.58E-14 |
| CKS_3206 | putative amidohydrolase | A | 5.97 | 3.54 | 3.74E-03 |
| CKS_4275 | hypothetical protein | A | 5.96 | 3.87 | 1.32E-05 |
| CKS_3622 | pyruvate/alpha-keto-acid decarboxylase | A | 5.95 | 4.09 | 1.21E-19 |
| CKS_1696 | magnesium transporter | A | 5.91 | 4.07 | 9.07E-15 |
| CKS_4764 | hypothetical protein | A | 5.90 | 3.89 | 1.01E-06 |
| CKS_4760 | permease component of an ABC superfamily ribose transporter | A | 5.89 | 4.04 | 1.99E-13 |
| CKS_5026 | hypothetical protein | A | 5.89 | 3.48 | 3.32E-03 |
| CKS_2921 | putative RNA polymerase sigma factor | A | 5.88 | 4.04 | 1.12E-12 |
| CKS_3540 | DNA-binding transcriptional dual regulator | A | 5.88 | 4.00 | 1.51E-08 |
| CKS_4914 | predicted protease | A | 5.87 | 4.04 | 3.01E-16 |
| CKS_3983 | pyruvate formate lyase activating enzyme 1 | A | 5.86 | 4.03 | 2.13E-12 |
| CKS_1698 | hypothetical protein | A | 5.86 | 4.04 | 5.43E-14 |
| CKS_4484 | 3-ketoacyl-CoA thiolase (thiolase I) | A | 5.85 | 4.03 | 5.09E-19 |
| CKS_3202 | ABC superfamily transporter | A | 5.84 | 3.98 | 1.49E-10 |
| CKS_3860 | molybdopterin biosynthesis protein C | A | 5.84 | 3.99 | 3.84E-13 |
| CKS_0941 | inosose dehydratase | A | 5.83 | 3.93 | 2.14E-08 |
| CKS_4376 | putative transcriptional regulator | A | 5.81 | 3.95 | 6.14E-08 |
| CKS_3516 | oligogalacturonide transporter | A | 5.79 | 3.94 | 5.00E-10 |
| CKS_4718 | acetate permease | A | 5.78 | 3.89 | 2.23E-08 |
| CKS_0965 | ATP-binding component of an ABC superfamily D-ala-D-ala transporter | A | 5.78 | 3.97 | 6.69E-15 |
| CKS_2401 | hypothetical protein | A | 5.77 | 3.85 | 1.10E-06 |
| CKS_0922 | periplasmic-binding component of an ABC superfamily leucine transporter | A | 5.76 | 3.95 | 1.85E-13 |
| CKS_4759 | ATP-binding component of an ABC superfamily ribose transporter | A | 5.76 | 3.96 | 9.04E-14 |
| CKS_4571 | virulence protein | A | 5.73 | 3.94 | 5.87E-15 |
| CKS_2641 | phage baseplate assembly protein V | A | 5.67 | 3.82 | 4.58E-10 |
| CKS_3471 | malto-oligosyltrehalose trehalohydrolase | A | 5.64 | 3.87 | 6.02E-11 |
| CKS_3520 | putative formate dehydrogenase oxidoreductase protein | A | 5.64 | 3.89 | 1.61E-15 |
| CKS_4577 | putative type III secretion system effector protein | A | 5.63 | 3.86 | 6.00E-17 |
| CKS_3241 | amidinotransferase family protein | A | 5.60 | 3.74 | 8.90E-09 |
| CKS_2588 | hypothetical protein | A | 5.59 | 3.27 | 7.97E-03 |
| CKS_2640 | hypothetical protein | A | 5.58 | 3.77 | 8.40E-11 |
| CKS_3150 | predicted mannonate dehydrogenase | A | 5.57 | 3.77 | 6.32E-11 |
| CKS_3853 | dethiobiotin synthetase | A | 5.56 | 3.78 | 4.02E-09 |
| CKS_5373 | mobilization protein | A | 5.55 | 3.83 | 7.52E-14 |
| CKS_0791 | predicted reductase | A | 5.54 | 3.73 | 1.49E-07 |
| CKS_0403 | hypothetical protein | A | 5.50 | 3.59 | 4.26E-06 |
| CKS_4569 | secreted effector protein | A | 5.49 | 3.79 | 8.20E-16 |
| CKS_0376 | putative phage transposase | A | 5.45 | 3.74 | 9.64E-13 |
| CKS_4010 | predicted metal-binding enzyme | A | 5.43 | 3.72 | 3.18E-16 |
| CKS_2496 | extracellular solute-binding protein family 5 | A | 5.39 | 3.63 | 4.24E-08 |
| CKS_2880 | predicted transporter | A | 5.36 | 3.67 | 2.88E-09 |
| CKS_2398 | hypothetical protein | A | 5.35 | 3.46 | 4.35E-04 |
| CKS_3219 | cellulose synthase (UDP-forming) | A | 5.34 | 3.60 | 2.44E-07 |
| CKS_3441 | putative TetR family transcriptional regulator | A | 5.33 | 3.69 | 2.69E-15 |
| CKS_4144 | hypothetical protein | A | 5.32 | 3.66 | 1.26E-09 |
| CKS_4922 | anaerobic ribonucleotide reductase activating protein | A | 5.31 | 3.66 | 2.90E-15 |
| CKS_4247 | putative alpha/beta hydrolase | A | 5.30 | 3.63 | 5.46E-08 |
| CKS_2299 | DNA-binding transcriptional repressor | A | 5.29 | 3.61 | 1.24E-09 |
| CKS_2389 | hypothetical protein | A | 5.28 | 3.63 | 9.02E-13 |
| CKS_2587 | hypothetical protein | A | 5.28 | 3.20 | 3.94E-03 |
| CKS_3955 | hypothetical protein | A | 5.27 | 3.55 | 2.22E-07 |
| CKS_4389 | membrane component of an ABC superfamily dipeptide transporter | A | 5.25 | 3.53 | 5.49E-07 |
| CKS_3490 | inner membrane subunit of SoxR-reducing complex | A | 5.24 | 3.62 | 5.40E-13 |
| CKS_2387 | hypothetical protein | A | 5.24 | 3.47 | 1.05E-05 |
| CKS_5000 | hypothetical protein | A | 5.23 | 3.53 | 9.25E-08 |
| CKS_5834 | hypothetical protein | A | 5.22 | 3.28 | 1.25E-03 |
| CKS_0375 | putative DNA-binding protein | A | 5.21 | 3.47 | 2.19E-05 |
| CKS_1509 | nitric oxide dioxygenase/dihydropteridine reductase 2 | A | 5.21 | 3.54 | 4.60E-08 |
| CKS_3442 | uncharacterized DUF1289 family protein | A | 5.18 | 3.53 | 5.29E-07 |
| CKS_4594 | hypothetical protein | A | 5.17 | 3.51 | 4.46E-10 |
| CKS_4485 | fatty acid oxidation complex subunit alpha | A | 5.17 | 3.57 | 2.63E-11 |
| CKS_5438 | hypothetical protein | A | 5.17 | 3.41 | 2.75E-06 |
| CKS_1045 | acetolactate synthase large subunit | A | 5.14 | 3.55 | 1.25E-12 |
| CKS_1384 | glycine decarboxylase PLP-dependent subunit (protein P) of glycine cleavage complex | A | 5.14 | 3.53 | 5.02E-18 |
| CKS_2526 | periplasmic-binding component of an ABC superfamily ribose transporter | A | 5.08 | 3.42 | 1.25E-05 |
| CKS_0054 | ATP-binding component of an ABC superfamily sulfate/thiosulfate transporter | A | 5.08 | 3.31 | 1.36E-04 |
| CKS_3401 | hypothetical protein | A | 5.07 | 3.51 | 4.26E-11 |
| CKS_3242 | nikkomycin biosynthesis domain protein | A | 5.07 | 3.46 | 1.23E-10 |
| CKS_3849 | adenosylmethionine-8-amino-7-oxononanoate aminotransferase | A | 5.05 | 3.47 | 2.29E-13 |
| CKS_3255 | type III secretion system cytoplasmic ATP synthase | A | 5.03 | 3.45 | 1.31E-11 |
| CKS_0289 | putative permease component of an ABC superfamily amino acid transporter | A | 5.03 | 3.04 | 1.09E-02 |
| CKS_3536 | L-ribulokinase | A | 5.02 | 3.38 | 8.23E-06 |
| CKS_2308 | hydroxypyruvate isomerase | A | 5.01 | 3.40 | 9.12E-07 |
| CKS_2399 | phage tail-like protein | A | 5.00 | 3.41 | 2.19E-08 |
| CKS_4288 | uncharacterized DUF1040 family protein | A | 5.00 | 3.42 | 8.18E-09 |
| CKS_4523 | type III secretion system peptide export protein | A | 4.98 | 3.43 | 4.98E-16 |
| CKS_2523 | putative hybrid two-component system regulatory protein | A | 4.98 | 3.39 | 9.48E-08 |
| CKS_2540 | IS66 family transposase | A | 4.97 | 3.38 | 2.26E-08 |
| CKS_1538 | hypothetical protein | A | 4.97 | 3.42 | 9.91E-09 |
| CKS_2369 | hypothetical protein | A | 4.96 | 3.30 | 1.81E-04 |
| CKS_1867 | putative ABC-type transport protein | A | 4.96 | 3.16 | 2.09E-03 |
| CKS_1857 | DNA-binding transcriptional dual regulator with FlhC | A | 4.96 | 3.39 | 2.34E-10 |
| CKS_0881 | uncharacterized DUF1471 family protein | A | 4.95 | 3.37 | 3.06E-05 |
| CKS_5446 | hypothetical protein | A | 4.94 | 3.41 | 4.60E-12 |
| CKS_4800 | predicted dehydrogenase | A | 4.94 | 3.33 | 2.70E-06 |
| CKS_3243 | hypothetical protein | A | 4.94 | 3.37 | 9.25E-11 |
| CKS_4162 | hypothetical protein | A | 4.93 | 3.19 | 1.13E-03 |
| CKS_3021 | putative NADH:flavin oxidoreductase | A | 4.92 | 3.36 | 1.57E-13 |
| CKS_4712 | hypothetical protein | A | 4.92 | 3.39 | 3.85E-12 |
| CKS_2550 | DgsA-binding anti-repressor | A | 4.91 | 3.34 | 1.69E-06 |
| CKS_2920 | hypothetical protein | A | 4.90 | 3.38 | 8.33E-10 |
| CKS_4956 | ABC transporter | A | 4.89 | 3.24 | 5.83E-04 |
| CKS_2350 | hypothetical protein | A | 4.85 | 3.26 | 2.72E-06 |
| CKS_2804 | putative carbon starvation protein A | A | 4.84 | 3.27 | 4.25E-05 |
| CKS_3144 | hypothetical protein | A | 4.83 | 3.25 | 4.53E-06 |
| CKS_3826 | PhoH family ATPase | A | 4.83 | 3.23 | 2.75E-06 |
| CKS_0943 | 5-deoxy-glucuronate isomerase | A | 4.80 | 3.32 | 2.61E-10 |
| CKS_2494 | type II haloacid dehalogenase | A | 4.80 | 3.15 | 9.16E-04 |
| CKS_0921 | membrane component of an ABC superfamily leucine/isoleucine/valine transporter | A | 4.79 | 3.29 | 6.57E-09 |
| CKS_4332 | PTS system cellobiose-specific IIC component | A | 4.78 | 3.27 | 9.32E-09 |
| CKS_0377 | phage transposase | A | 4.76 | 3.17 | 1.09E-04 |
| CKS_3257 | type III secretion system inner membrane channel protein | A | 4.76 | 3.27 | 9.03E-11 |
| CKS_4016 | ATP-binding component of an ABC superfamily alkanesulfonate transporter | A | 4.76 | 3.10 | 6.53E-04 |
| CKS_0640 | zinc-containing alcohol dehydrogenase/quinone oxidoreductase | A | 4.76 | 3.29 | 7.87E-13 |
| CKS_1811 | phosphoribosylglycinamide formyltransferase 2 | A | 4.76 | 3.29 | 6.96E-12 |
| CKS_2361 | DNA adenine methylase | A | 4.74 | 3.22 | 2.74E-07 |
| CKS_4591 | secretion system apparatus protein | A | 4.73 | 3.26 | 1.83E-15 |
| CKS_2363 | phage antitermination protein Q | A | 4.70 | 3.20 | 3.69E-06 |
| CKS_1546 | hypothetical protein | A | 4.68 | 3.22 | 1.45E-06 |
| CKS_5025 | phage-related protein | A | 4.68 | 2.82 | 1.62E-02 |
| CKS_0221 | regulator of penicillin binding proteins and beta lactamase transcription (morphogene) | A | 4.68 | 3.22 | 2.13E-10 |
| CKS_5455 | putative exported protein | A | 4.68 | 3.14 | 4.48E-04 |
| CKS_3205 | predicted monooxygenase | A | 4.64 | 2.87 | 1.15E-02 |
| CKS_3273 | HrpT family protein | A | 4.64 | 3.16 | 2.03E-07 |
| CKS_4592 | putative type III secretion system ATP synthase | A | 4.60 | 3.19 | 7.52E-14 |
| CKS_2015 | acetyl-CoA acetyltransferase | A | 4.59 | 3.16 | 2.12E-06 |
| CKS_1835 | hypothetical protein | A | 4.58 | 3.14 | 1.04E-11 |
| CKS_5052 | YaiA family protein | A | 4.57 | 3.12 | 2.42E-05 |
| CKS_2644 | hypothetical protein | A | 4.57 | 3.06 | 1.22E-05 |
| CKS_1436 | putative transposase | A | 4.57 | 3.12 | 1.11E-06 |
| CKS_3386 | hypothetical protein | A | 4.55 | 3.13 | 3.04E-07 |
| CKS_3701 | ABC superfamily transporter | A | 4.55 | 3.08 | 5.20E-05 |
| CKS_2524 | ATP-binding component of an ABC superfamily ribose transporter | A | 4.55 | 3.09 | 1.85E-06 |
| CKS_4055 | putative cytoplasmic protein | A | 4.54 | 3.11 | 5.07E-06 |
| CKS_1044 | acetolactate synthase small subunit | A | 4.54 | 3.10 | 3.78E-10 |
| CKS_5349 | putative exported protein | A | 4.53 | 3.05 | 3.21E-04 |
| CKS_0960 | putative RpiR family transcriptional regulator | A | 4.52 | 3.10 | 7.58E-06 |
| CKS_5754 | hypothetical protein | A | 4.51 | 2.75 | 1.62E-02 |
| CKS_5332 | hypothetical protein | A | 4.51 | 3.03 | 7.86E-06 |
| CKS_2589 | phage-related protein | A | 4.50 | 2.73 | 1.91E-02 |
| CKS_2306 | uncharacterized DUF1537 family protein | A | 4.50 | 3.05 | 1.91E-04 |
| CKS_1516 | inositol monophosphatase | A | 4.50 | 3.03 | 7.45E-08 |
| CKS_4567 | secretion system regulatory protein | A | 4.50 | 3.08 | 6.49E-09 |
| CKS_1463 | NrdI family ribonucleotide reduction protein | A | 4.49 | 3.00 | 1.18E-03 |
| CKS_0658 | altronate oxidoreductase NAD-dependent | A | 4.49 | 3.10 | 2.21E-08 |
| CKS_3968 | cold shock protein | A | 4.48 | 3.05 | 2.64E-04 |
| CKS_5389 | putative exported protein | A | 4.47 | 3.01 | 6.53E-04 |
| CKS_3939 | putative transport system permease protein | A | 4.46 | 3.10 | 3.81E-11 |
| CKS_1713 | hypothetical protein | A | 4.46 | 3.02 | 2.53E-06 |
| CKS_0516 | 34-dihydroxy-2-butanone-4-phosphate synthase | A | 4.46 | 3.06 | 8.45E-13 |
| CKS_5413 | hypothetical protein | A | 4.45 | 3.07 | 3.69E-08 |
| CKS_1289 | endoribonuclease L-PSP | A | 4.45 | 3.08 | 2.65E-13 |
| CKS_3552 | mannose-6-phosphate isomerase | A | 4.44 | 3.03 | 1.37E-06 |
| CKS_4462 | HD superfamily hydrolase | A | 4.42 | 3.02 | 4.41E-06 |
| CKS_5393 | putative exported protein | A | 4.42 | 2.97 | 1.25E-03 |
| CKS_3315 | Acyltransferase | A | 4.42 | 3.06 | 2.00E-12 |
| CKS_3844 | membrane component of an ABC superfamily molybdate transporter | A | 4.42 | 3.05 | 1.22E-12 |
| CKS_3145 | Na+/solute symporter | A | 4.41 | 3.02 | 6.31E-07 |
| CKS_2307 | predicted class II aldolase | A | 4.38 | 2.97 | 1.19E-05 |
| CKS_2905 | putative sugar transporter | A | 4.38 | 3.02 | 1.78E-07 |
| CKS_4941 | hypothetical protein | A | 4.36 | 3.01 | 4.89E-09 |
| CKS_0970 | predicted universal stress (ethanol tolerance) protein B | A | 4.35 | 2.98 | 7.34E-10 |
| CKS_0773 | uncharacterized DUF1471 family protein | A | 4.35 | 2.75 | 7.69E-03 |
| CKS_3470 | malto-oligosyltrehalose synthase | A | 4.34 | 2.99 | 2.20E-07 |
| CKS_2947 | maltose regulon periplasmic protein | A | 4.33 | 2.98 | 1.52E-06 |
| CKS_2637 | hypothetical protein | A | 4.33 | 2.96 | 4.31E-09 |
| CKS_0887 | maltodextrin phosphorylase | A | 4.33 | 3.00 | 4.44E-08 |
| CKS_3252 | type III secretion system protein | A | 4.33 | 2.99 | 5.82E-09 |
| CKS_0598 | methyl-accepting chemotaxis sensory transducer | A | 4.32 | 2.95 | 1.25E-07 |
| CKS_5199 | LysR family transcriptional regulator | A | 4.32 | 3.00 | 1.25E-14 |
| CKS_3147 | alcohol dehydrogenase | A | 4.30 | 2.91 | 3.78E-05 |
| CKS_4192 | dihydro-orotase | A | 4.27 | 2.97 | 3.60E-10 |
| CKS_3143 | hypothetical protein | A | 4.26 | 2.85 | 1.66E-04 |
| CKS_3462 | predicted FAD-binding phosphodiesterase | A | 4.26 | 2.96 | 1.27E-13 |
| CKS_4768 | predicted L-xylulose 5-phosphate 3-epimerase | A | 4.24 | 2.80 | 2.18E-04 |
| CKS_2392 | hypothetical protein | A | 4.23 | 2.92 | 3.94E-08 |
| CKS_5356 | putative outer membrane adhesion protein | A | 4.23 | 2.93 | 2.21E-13 |
| CKS_2050 | membrane component of an ABC superfamily histidine/lysine/arginine/ornithine transporter | A | 4.23 | 2.92 | 2.51E-08 |
| CKS_2505 | AsnC family transcriptional regulator | A | 4.20 | 2.89 | 1.45E-09 |
| CKS_2017 | phosphohistidine phosphatase | A | 4.19 | 2.91 | 2.52E-14 |
| CKS_3210 | predicted oxidoreductase flavin:NADH component | A | 4.18 | 2.64 | 1.51E-02 |
| CKS_2105 | large repetitive protein | A | 4.18 | 2.87 | 1.76E-05 |
| CKS_5039 | branched chain amino acid transporter (LIV-II) | A | 4.18 | 2.88 | 3.41E-12 |
| CKS_5330 | putative outer membrane adhesion protein | A | 4.18 | 2.87 | 1.21E-07 |
| CKS_2590 | hypothetical protein | A | 4.17 | 2.52 | 4.03E-02 |
| CKS_2498 | binding-protein-dependent transport systems inner membrane component | A | 4.17 | 2.71 | 5.60E-03 |
| CKS_0285 | gamma-glutamyltranspeptidase | A | 4.17 | 2.63 | 2.33E-02 |
| CKS_1444 | GroES family alcohol dehydrogenase | A | 4.16 | 2.89 | 1.90E-08 |
| CKS_1664 | anti-sigma factor | A | 4.15 | 2.86 | 7.41E-10 |
| CKS_3277 | putative avirulence protein | A | 4.14 | 2.86 | 1.11E-07 |
| CKS_1861 | trehalose-6-phosphate phosphatase | A | 4.14 | 2.88 | 4.40E-13 |
| CKS_2228 | multidrug efflux system subunit A | A | 4.12 | 2.83 | 8.24E-10 |
| CKS_1517 | tRNA:Cm32/Um32 methyltransferase | A | 4.12 | 2.82 | 2.97E-08 |
| CKS_1464 | glutaredoxin-like protein | A | 4.12 | 2.77 | 2.56E-03 |
| CKS_4630 | Cellulose | A | 4.09 | 2.79 | 3.83E-08 |
| CKS_2706 | PLP-binding alanine racemase 2 | A | 4.08 | 2.83 | 2.91E-07 |
| CKS_4828 | L-arabinolactonase | A | 4.08 | 2.76 | 1.92E-05 |
| CKS_2629 | phage tail sheath protein FI | A | 4.08 | 2.75 | 3.16E-06 |
| CKS_2506 | thiamine pyrophosphate protein TPP binding domain protein | A | 4.07 | 2.83 | 3.85E-09 |
| CKS_3594 | hypothetical protein | A | 4.06 | 2.80 | 5.78E-05 |
| CKS_0600 | hypothetical protein | A | 4.06 | 2.55 | 1.95E-02 |
| CKS_4298 | glutamine synthetase | A | 4.05 | 2.80 | 1.61E-09 |
| CKS_1870 | hydroxyacylglutathione hydrolase | A | 4.05 | 2.80 | 2.37E-06 |
| CKS_3854 | putative amino acid transporter | A | 4.04 | 2.63 | 5.31E-03 |
| CKS_2899 | salicylate hydroxylase | A | 4.04 | 2.59 | 1.14E-02 |
| CKS_4580 | hypothetical protein | A | 4.03 | 2.78 | 1.14E-09 |
| CKS_0272 | phytanoyl-CoA dioxygenase family protein | A | 4.01 | 2.68 | 1.11E-03 |
| CKS_4983 | GGDEF domain-containing protein | A | 4.00 | 2.78 | 1.09E-08 |
| CKS_0016 | 50S ribosomal subunit protein L7/L12 | R | 4.02 | 5.63 | 1.24E-33 |
| CKS_4368 | superoxide dismutase Mn | R | 4.05 | 5.50 | 2.36E-19 |
| CKS_0117 | peptidyl-prolyl cis-trans isomerase B (rotamase B) | R | 4.08 | 5.65 | 2.64E-30 |
| CKS_4123 | putative bacteriophage protein | R | 4.09 | 5.66 | 8.22E-23 |
| CKS_1240 | chorismate mutase I/cyclohexadienyl dehydrogenase | R | 4.16 | 5.72 | 1.59E-17 |
| CKS_4772 | aerobactin siderophore receptor | R | 4.25 | 5.51 | 5.25E-11 |
| CKS_0364 | LysR family transcriptional regulator | R | 4.34 | 5.96 | 2.87E-26 |
| CKS_4239 | PTS system glucose-specific IICB component | R | 4.35 | 5.94 | 1.68E-25 |
| CKS_4125 | putative bacteriophage protein | R | 4.42 | 6.15 | 1.05E-26 |
| CKS_4121 | putative bacteriophage protein | R | 4.51 | 6.25 | 5.40E-26 |
| CKS_0004 | HU DNA-binding transcriptional regulator alpha subunit | R | 4.58 | 6.30 | 2.94E-30 |
| CKS_3013 | L-24-diaminobutyrate decarboxylase | R | 4.59 | 6.26 | 1.14E-14 |
| CKS_4122 | hypothetical protein | R | 4.69 | 6.39 | 3.07E-19 |
| CKS_0095 | hypothetical protein | R | 4.72 | 6.09 | 1.20E-08 |
| CKS_4134 | putative bacteriophage protein | R | 4.76 | 6.59 | 1.52E-25 |
| CKS_0017 | 50S ribosomal subunit protein L10 | R | 4.80 | 6.68 | 1.54E-32 |
| CKS_5198 | dihydrolipoamide dehydrogenase | R | 4.82 | 6.71 | 1.23E-30 |
| CKS_0744 | 30S ribosomal subunit protein S6 | R | 4.84 | 6.71 | 1.34E-25 |
| CKS_4717 | putative exported protein | R | 4.87 | 6.75 | 3.04E-28 |
| CKS_5580 | phage lysozyme | R | 4.89 | 5.12 | 2.09E-03 |
| CKS_5259 | protein chain elongation factor EF-Ts | R | 4.97 | 6.84 | 7.21E-21 |
| CKS_4536 | type III secretion system effector protein | R | 4.99 | 6.86 | 3.72E-30 |
| CKS_4535 | type III secretion system regulatory protein | R | 5.36 | 7.22 | 5.63E-19 |
| CKS_0742 | 30S ribosomal subunit protein S18 | R | 5.52 | 7.61 | 3.50E-27 |
| CKS_5601 | hypothetical protein | R | 5.55 | 6.00 | 2.05E-04 |
| CKS_4124 | hypothetical protein | R | 5.71 | 7.77 | 5.69E-23 |
| CKS_1527 | nucleoside diphosphate kinase | R | 5.76 | 7.90 | 6.96E-32 |
| CKS_4120 | putative bacteriophage protein | R | 5.78 | 8.02 | 3.40E-41 |
| CKS_4099 | putative phage primase | R | 5.88 | 7.78 | 1.78E-13 |
| CKS_4115 | putative bacteriophage protein | R | 6.00 | 8.32 | 2.15E-40 |
| CKS_4116 | putative bacteriophage protein | R | 6.00 | 8.20 | 3.71E-27 |
| CKS_5565 | antiterminator Q | R | 6.06 | 5.68 | 2.13E-03 |
| CKS_5581 | hypothetical protein | R | 6.07 | 6.22 | 3.73E-04 |
| CKS_0743 | primosomal replication protein N | R | 6.09 | 8.40 | 4.73E-30 |
| CKS_4117 | putative bacteriophage protein | R | 6.11 | 8.39 | 1.49E-34 |
| CKS_4137 | hypothetical protein | R | 6.20 | 8.34 | 4.09E-19 |
| CKS_4135 | putative bacteriophage protein | R | 6.25 | 8.45 | 9.85E-21 |
| CKS_4109 | putative bacteriophage protein | R | 6.28 | 8.66 | 1.24E-34 |
| CKS_0741 | 50S ribosomal subunit protein L9 | R | 6.33 | 8.73 | 1.54E-32 |
| CKS_0094 | hypothetical protein | R | 6.37 | 8.16 | 6.37E-12 |
| CKS_5585 | hypothetical protein | R | 6.56 | 6.92 | 8.70E-05 |
| CKS_1116 | ADP-heptose--lipooligosaccharide heptosyltransferase II | R | 6.57 | 9.01 | 3.81E-46 |
| CKS_4113 | hypothetical protein | R | 6.72 | 9.24 | 3.55E-34 |
| CKS_5130 | putative outer membrane protein | R | 6.83 | 8.87 | 1.16E-13 |
| CKS_5588 | hypothetical protein | R | 7.25 | 4.86 | 1.75E-02 |
| CKS_4114 | putative bacteriophage protein | R | 7.32 | 10.06 | 1.57E-44 |
| CKS_0132 | hypothetical protein | R | 7.44 | 9.91 | 9.62E-25 |
| CKS_4119 | putative bacteriophage protein | R | 7.55 | 10.37 | 7.44E-42 |
| CKS_4531 | type III secretion system apparatus protein | R | 7.65 | 10.55 | 3.77E-55 |
| CKS_4091 | hypothetical protein | R | 7.82 | 10.47 | 3.37E-23 |
| CKS_4118 | putative bacteriophage protein | R | 7.99 | 10.75 | 2.87E-31 |
| CKS_4092 | hypothetical protein | R | 8.20 | 11.12 | 8.61E-31 |
| CKS_4136 | putative bacteriophage tail protein | R | 8.32 | 11.27 | 6.23E-31 |
| CKS_1241 | 3-deoxy-D-arabino-heptulosonate-7-phosphate synthase tyrosine-repressible | R | 8.70 | 10.58 | 8.06E-12 |
| CKS_4538 | type III secretion system apparatus protein | R | 8.80 | 11.97 | 2.47E-53 |
| CKS_1225 | hypothetical protein | R | 8.91 | 12.00 | 2.40E-39 |
| CKS_4095 | hypothetical protein | R | 9.89 | 13.00 | 1.33E-24 |
| CKS_5564 | Cro repressor | R | 10.16 | 6.62 | 2.75E-03 |
| CKS_5584 | membrane glycoprotein | R | 11.66 | 5.97 | 7.91E-03 |
| CKS_4089 | hypothetical protein | R | 13.42 | 17.32 | 1.10E-28 |
| CKS_4090 | hypothetical protein | R | 13.63 | 17.96 | 6.46E-36 |
| CKS_5586 | hypothetical protein | R | 14.19 | 11.73 | 3.52E-06 |
| CKS_4088 | hypothetical protein | R | 16.02 | 20.13 | 1.25E-28 |
| CKS_4082 | hypothetical protein | R | 16.02 | 19.51 | 1.72E-22 |
| CKS_4093 | CP4-57 family phage integrase | R | 16.35 | 20.69 | 9.41E-29 |
| CKS_4081 | hypothetical protein | R | 16.79 | 20.73 | 1.75E-25 |
| CKS_4084 | phage lysozyme | R | 17.69 | 21.56 | 2.40E-24 |
| CKS_4537 | type III secretion system effector protein | R | 18.27 | 23.86 | 8.75E-59 |
| CKS_4085 | hypothetical protein | R | 18.62 | 22.54 | 3.47E-24 |
| CKS_5587 | hypothetical protein | R | 21.31 | 10.26 | 1.96E-04 |
| CKS_4083 | hypothetical protein | R | 22.82 | 28.20 | 3.45E-33 |
| CKS_4087 | hypothetical protein | R | 25.57 | 31.54 | 3.41E-36 |

^a^A = activated in the *in planta* culture compared to the pre-inoculum liquid culture (lower in liquid culture), R = repressed in the *in planta* culture compared to the pre-inoculum liquid culture (higher in liquid culture)
