# Supplementary material for: Analysis of the in planta transcriptome expressed by the corn pathogen Pantoea stewartii subsp. stewartii via RNA-Seq
Source: PeerJ. 2017 Apr 27;5:e3237. doi: 10.7717/peerj.3237 (PMC5410145; doi:10.7717/peerj.3237)
Supplement: Table S3 — a A = activated in the in planta culture compared to in vitro plate culture (lower in plate culture), R = repressed in the in planta culture compared to in vitro plate culture (higher in plate culture). [file peerj-05-3237-s003.docx]

**Table S3**. RNA-Seq data of differentially expressed genes between the *in planta* culture and the *in vitro* plate culture ^a^

| **Locus Tag** | **Annotation** | | **RPM Fold Regulation** | **DESeq Fold Regulation** | **DESeq padj** |
| --- | --- | --- | --- | --- | --- |
| CKS_3692 | hypothetical protein | A | 73.74 | 45.35 | 2.03E-34 |
| CKS_3355 | periplasmic-binding component of an ABC superfamily ribose transporter | A | 69.73 | 45.91 | 3.79E-45 |
| CKS_4725 | hypothetical protein | A | 65.98 | 38.91 | 3.97E-27 |
| CKS_3263 | HrpA family pilus protein | A | 58.52 | 41.81 | 3.36E-90 |
| CKS_2320 | putative alkanal monooxygenase | A | 52.46 | 34.16 | 8.32E-36 |
| CKS_0368 | L-lactate dehydrogenase FMN-linked | A | 48.38 | 29.49 | 2.46E-21 |
| CKS_3537 | periplasmic-binding component of an ABC superfamily L-arabinose transporter | A | 46.70 | 33.82 | 1.08E-82 |
| CKS_3570 | AraC family transcriptional regulator | A | 45.70 | 32.15 | 2.94E-52 |
| CKS_0306 | medium-long-chain fatty acyl-CoA dehydrogenase | A | 42.17 | 30.62 | 4.68E-89 |
| CKS_5750 | acid shock protein | A | 40.09 | 10.72 | 3.29E-04 |
| CKS_4485 | fatty acid oxidation complex subunit alpha | A | 37.14 | 27.45 | 1.63E-105 |
| CKS_2015 | acetyl-CoA acetyltransferase | A | 35.62 | 25.32 | 5.44E-41 |
| CKS_2379 | nudix hydrolase | A | 33.59 | 23.10 | 4.93E-33 |
| CKS_2184 | periplasmic-binding component of an ABC superfamily methyl-galactoside transporter | A | 33.27 | 24.34 | 1.08E-69 |
| CKS_0367 | DNA-binding transcriptional repressor | A | 33.05 | 13.32 | 8.25E-06 |
| CKS_1610 | ATP-binding component of an ABC superfamily taurine transporter | A | 32.62 | 10.90 | 1.40E-04 |
| CKS_3319 | predicted inner membrane protein | A | 31.76 | 23.54 | 2.79E-82 |
| CKS_3793 | cytochrome d ubiquinol oxidase subunit I | A | 31.45 | 11.51 | 7.77E-05 |
| CKS_2380 | hypothetical protein | A | 30.37 | 21.03 | 1.87E-30 |
| CKS_5511 | hypothetical protein | A | 29.97 | 18.60 | 5.55E-18 |
| CKS_3621 | aldo-keto reductase | A | 29.63 | 21.18 | 1.64E-38 |
| CKS_2185 | ATP-binding component of an ABC superfamily galactose/methyl galactoside transporter | A | 28.73 | 20.86 | 2.60E-54 |
| CKS_3320 | GGDEF domain protein | A | 28.09 | 20.92 | 1.14E-75 |
| CKS_2806 | putative formate dehydrogenase oxidoreductase protein | A | 27.14 | 10.41 | 1.42E-04 |
| CKS_3354 | short-chain dehydrogenase/reductase | A | 26.87 | 19.19 | 4.03E-36 |
| CKS_1609 | membrane component of an ABC superfamily taurine transporter | A | 26.81 | 10.15 | 1.33E-04 |
| CKS_0948 | mannonate hydrolase | A | 26.64 | 18.93 | 8.50E-32 |
| CKS_0338 | glycerol dehydrogenase NAD | A | 25.84 | 18.09 | 7.39E-25 |
| CKS_3039 | hypothetical protein | A | 25.68 | 18.73 | 1.64E-41 |
| CKS_0373 | PTS system cellobiose-specific IIB component | A | 25.25 | 17.55 | 8.23E-24 |
| CKS_3794 | cytochrome d terminal oxidase subunit II | A | 24.97 | 16.45 | 2.29E-15 |
| CKS_0379 | hypothetical protein | A | 24.08 | 16.13 | 6.57E-21 |
| CKS_3795 | membrane-bound protein | A | 23.60 | 15.97 | 2.84E-17 |
| CKS_2014 | 3-ketoacyl-CoA thiolase | A | 23.54 | 17.04 | 2.03E-34 |
| CKS_0375 | putative DNA-binding protein | A | 23.08 | 15.35 | 2.53E-18 |
| CKS_2163 | IIA and HPr components of fructose-specific PTS enzyme | A | 23.05 | 16.32 | 6.50E-26 |
| CKS_2442 | aldehyde dehydrogenase B | A | 22.99 | 16.75 | 2.90E-44 |
| CKS_3356 | hypothetical protein | A | 22.66 | 15.67 | 1.50E-22 |
| CKS_3275 | HrpN family hypersensitivity reaction elicitor | A | 21.21 | 15.36 | 5.62E-33 |
| CKS_1611 | periplasmic-binding component of an ABC superfamily taurine transporter | A | 21.18 | 7.82 | 1.27E-03 |
| CKS_3270 | HrpF family protein | A | 20.78 | 15.48 | 1.21E-60 |
| CKS_0515 | predicted dioxygenase | A | 19.90 | 13.94 | 1.48E-27 |
| CKS_5455 | putative exported protein | A | 18.99 | 13.41 | 2.61E-21 |
| CKS_2164 | fructose-1-phosphate kinase | A | 18.24 | 13.25 | 1.57E-33 |
| CKS_5349 | putative exported protein | A | 17.83 | 12.65 | 2.79E-22 |
| CKS_5393 | putative exported protein | A | 17.45 | 12.35 | 4.29E-20 |
| CKS_0380 | hypothetical protein | A | 17.17 | 11.01 | 1.05E-10 |
| CKS_0366 | L-lactate permease | A | 16.85 | 11.16 | 2.03E-11 |
| CKS_0801 | ATP-binding component of an ABC superfamily predicted amino-acid transporter | A | 16.83 | 12.04 | 1.23E-31 |
| CKS_5389 | putative exported protein | A | 16.57 | 11.79 | 5.24E-21 |
| CKS_3968 | cold shock protein | A | 16.43 | 11.99 | 1.34E-32 |
| CKS_0376 | putative phage transposase | A | 16.09 | 11.84 | 4.39E-35 |
| CKS_0649 | 24-dienoyl-CoA reductase NADH and FMN-linked | A | 15.71 | 11.75 | 2.08E-56 |
| CKS_4657 | malate synthase A | A | 15.65 | 11.35 | 1.23E-26 |
| CKS_3021 | putative NADH:flavin oxidoreductase | A | 15.50 | 11.47 | 1.32E-30 |
| CKS_0570 | thymidine phosphorylase | A | 15.40 | 11.43 | 8.99E-35 |
| CKS_4830 | amidohydrolase | A | 15.14 | 11.06 | 2.80E-33 |
| CKS_1591 | bacterioferritin iron storage and detoxification protein | A | 14.97 | 10.99 | 1.79E-24 |
| CKS_4767 | 3-keto-L-gulonate 6-phosphate decarboxylase | A | 14.79 | 9.95 | 1.39E-12 |
| CKS_4956 | ABC transporter | A | 14.79 | 10.72 | 1.78E-31 |
| CKS_4575 | secretion system chaperone | A | 14.78 | 10.67 | 1.38E-29 |
| CKS_4463 | sn-glycerol-3-phosphate transporter | A | 14.69 | 10.66 | 1.53E-22 |
| CKS_5579 | lysis protein S | A | 14.59 | 9.37 | 6.02E-10 |
| CKS_3407 | phosphoenolpyruvate synthase | A | 14.54 | 10.61 | 1.67E-24 |
| CKS_0960 | putative RpiR family transcriptional regulator | A | 14.48 | 10.78 | 2.88E-37 |
| CKS_0687 | predicted pirin-related protein | A | 14.37 | 9.96 | 6.48E-17 |
| CKS_2804 | putative carbon starvation protein A | A | 14.30 | 10.32 | 1.37E-21 |
| CKS_2016 | enoyl-CoA hydratase/3-hydroxyacyl-CoA dehydrogenase | A | 14.07 | 10.54 | 2.75E-44 |
| CKS_2488 | ATP-binding component of an ABC superfamily sugar transporter | A | 13.75 | 10.25 | 3.98E-41 |
| CKS_4955 | permease component of an ABC superfamily transporter | A | 13.66 | 9.71 | 2.11E-20 |
| CKS_3540 | DNA-binding transcriptional dual regulator | A | 13.66 | 10.10 | 4.78E-32 |
| CKS_4279 | hypothetical protein | A | 13.27 | 9.47 | 3.08E-18 |
| CKS_0668 | predicted quinol oxidase subunit | A | 13.06 | 9.01 | 2.40E-15 |
| CKS_5753 | hypothetical protein | A | 13.03 | 9.80 | 9.61E-49 |
| CKS_4281 | hypothetical protein | A | 12.99 | 9.05 | 3.47E-14 |
| CKS_4019 | alkanesulfonate monooxygenase FMNH(2)-dependent | A | 12.90 | 8.74 | 4.06E-12 |
| CKS_2186 | membrane component of an ABC superfamily methyl-galactoside transporter | A | 12.73 | 9.37 | 7.14E-30 |
| CKS_2504 | gamma-aminobutyrate:alpha-ketoglutarate aminotransferase | A | 12.38 | 9.16 | 9.59E-27 |
| CKS_4653 | periplasmic binding protein | A | 12.34 | 9.30 | 2.74E-45 |
| CKS_4759 | ATP-binding component of an ABC superfamily ribose transporter | A | 12.00 | 8.88 | 5.25E-30 |
| CKS_4658 | isocitrate lyase | A | 11.88 | 8.53 | 7.22E-16 |
| CKS_2680 | mannose-specific enzyme IIC component of PTS | A | 11.86 | 8.65 | 4.28E-19 |
| CKS_2375 | hypothetical protein | A | 11.85 | 8.29 | 4.81E-11 |
| CKS_2765 | hypothetical protein | A | 11.83 | 8.78 | 1.46E-22 |
| CKS_4576 | putative type III secretion system effector protein | A | 11.82 | 8.83 | 1.19E-38 |
| CKS_2372 | putative transcriptional regulator | A | 11.80 | 8.39 | 1.24E-15 |
| CKS_0377 | phage transposase | A | 11.68 | 8.26 | 7.13E-13 |
| CKS_2383 | hypothetical protein | A | 11.68 | 8.21 | 1.64E-12 |
| CKS_4331 | PTS system cellobiose-specific IIB component | A | 11.55 | 8.31 | 2.84E-15 |
| CKS_3796 | YbgE family protein | A | 11.51 | 8.43 | 6.66E-16 |
| CKS_4719 | conserved inner membrane protein involved in acetate transport | A | 11.47 | 8.04 | 1.97E-12 |
| CKS_4953 | hypothetical protein | A | 11.42 | 8.30 | 3.49E-22 |
| CKS_4579 | hypothetical protein | A | 11.19 | 8.13 | 1.90E-22 |
| CKS_3715 | hypothetical protein | A | 11.14 | 8.35 | 3.60E-36 |
| CKS_3265 | HrpB family protein | A | 11.13 | 8.23 | 1.45E-24 |
| CKS_0940 | myo-inositol 2-dehydrogenase | A | 11.09 | 8.10 | 6.26E-16 |
| CKS_2366 | hypothetical protein | A | 11.00 | 7.94 | 4.82E-16 |
| CKS_1750 | N-acetylmuramoyl-L-alanine amidase | A | 10.94 | 7.95 | 1.83E-15 |
| CKS_5150 | 3-isopropylmalate isomerase subunit dehydratase component | A | 10.93 | 8.12 | 1.21E-23 |
| CKS_2985 | hypothetical protein | A | 10.92 | 7.65 | 2.31E-11 |
| CKS_3569 | RND multidrug efflux membrane fusion protein | A | 10.87 | 7.88 | 9.80E-19 |
| CKS_3539 | L-arabinose transport system permease protein | A | 10.82 | 7.93 | 2.05E-20 |
| CKS_4282 | hypothetical protein | A | 10.81 | 7.83 | 3.98E-19 |
| CKS_2384 | hypothetical protein | A | 10.62 | 7.52 | 3.22E-11 |
| CKS_3201 | membrane component of an ABC superfamily polar amino acid transporter | A | 10.61 | 7.79 | 2.49E-20 |
| CKS_2487 | putative periplasmic binding protein | A | 10.60 | 7.77 | 2.14E-26 |
| CKS_0962 | periplasmic-binding component of an ABC superfamily D-ala-D-a la transporter | A | 10.58 | 7.96 | 1.18E-34 |
| CKS_0390 | hypothetical protein | A | 10.51 | 7.61 | 5.83E-12 |
| CKS_3767 | hypothetical protein | A | 10.45 | 7.66 | 4.96E-20 |
| CKS_3267 | type III secretion system lipoprotein | A | 10.36 | 7.68 | 2.34E-23 |
| CKS_1289 | endoribonuclease L-PSP | A | 9.98 | 7.47 | 2.31E-33 |
| CKS_5413 | hypothetical protein | A | 9.95 | 7.43 | 4.60E-20 |
| CKS_3512 | oxidoreductase domain protein | A | 9.87 | 7.33 | 1.84E-25 |
| CKS_2586 | putative adenine methylase | A | 9.87 | 6.60 | 2.75E-07 |
| CKS_0891 | aerobic FAD/NAD(P)-binding sn-glycerol-3-phosphate dehydrogenase | A | 9.84 | 7.44 | 6.94E-42 |
| CKS_3022 | anion transporter | A | 9.81 | 7.24 | 5.71E-15 |
| CKS_1749 | hypothetical protein | A | 9.78 | 7.26 | 9.65E-18 |
| CKS_5454 | putative cell wall-associated hydrolase | A | 9.73 | 7.30 | 5.25E-26 |
| CKS_1089 | glycerol facilitator | A | 9.60 | 7.18 | 3.98E-29 |
| CKS_4574 | putative type III secretion system effector protein | A | 9.57 | 7.04 | 7.08E-24 |
| CKS_1044 | acetolactate synthase small subunit | A | 9.52 | 7.11 | 4.13E-23 |
| CKS_3579 | putative MFS superfamily benzoate transporter | A | 9.44 | 7.09 | 9.11E-30 |
| CKS_0964 | membrane component of an ABC superfamily D-ala-D-ala transporter | A | 9.44 | 7.01 | 2.89E-23 |
| CKS_4954 | aryldialkylphosphatase | A | 9.41 | 6.97 | 5.42E-23 |
| CKS_0560 | hypothetical protein | A | 9.35 | 7.08 | 7.33E-36 |
| CKS_2855 | pyridine nucleotide transhydrogenase beta subunit | A | 9.35 | 6.84 | 9.54E-13 |
| CKS_3254 | HrpO family protein | A | 9.32 | 6.71 | 8.43E-13 |
| CKS_2381 | hypothetical protein | A | 9.28 | 6.92 | 2.24E-19 |
| CKS_2373 | hypothetical protein | A | 9.27 | 6.68 | 6.90E-14 |
| CKS_1233 | protein disaggregation chaperone | A | 9.25 | 6.54 | 1.60E-11 |
| CKS_4654 | hypothetical protein | A | 9.24 | 6.96 | 4.88E-33 |
| CKS_4586 | secretion system apparatus protein | A | 9.23 | 6.97 | 2.28E-30 |
| CKS_3513 | oxidoreductase domain protein | A | 9.22 | 6.91 | 3.47E-26 |
| CKS_4829 | integral membrane protein | A | 9.19 | 6.51 | 4.31E-12 |
| CKS_1037 | predicted transporter | A | 9.17 | 6.92 | 2.16E-26 |
| CKS_4792 | Maltoporin | A | 9.09 | 6.80 | 1.19E-24 |
| CKS_0961 | D-ala-D-ala dipeptidase Zn-dependent | A | 9.07 | 6.82 | 5.29E-27 |
| CKS_4585 | putative type III secretion system apparatus protein | A | 9.04 | 6.80 | 2.83E-22 |
| CKS_2370 | heptosyltransferase | A | 8.94 | 6.44 | 3.97E-12 |
| CKS_4247 | putative alpha/beta hydrolase | A | 8.92 | 6.62 | 1.16E-13 |
| CKS_4588 | type III secretion system apparatus protein | A | 8.87 | 6.60 | 2.10E-18 |
| CKS_4333 | beta-glucosidase | A | 8.87 | 6.61 | 8.21E-19 |
| CKS_2182 | DNA-binding transcriptional repressor | A | 8.82 | 6.62 | 1.00E-26 |
| CKS_4320 | Epimerase | A | 8.79 | 6.39 | 3.37E-14 |
| CKS_3281 | HopAM1-1 family type III effector | A | 8.75 | 6.45 | 1.79E-19 |
| CKS_0374 | PTS system cellobiose-specific IIC component | A | 8.74 | 6.50 | 2.38E-19 |
| CKS_3948 | predicted inner membrane protein | A | 8.73 | 6.53 | 2.38E-25 |
| CKS_2791 | hypothetical protein | A | 8.70 | 6.55 | 2.31E-33 |
| CKS_4573 | hypothetical protein | A | 8.67 | 6.40 | 1.30E-20 |
| CKS_4761 | periplasmic substrate-binding component of an ABC superfamily ribose transporter | A | 8.66 | 6.50 | 4.52E-29 |
| CKS_4577 | putative type III secretion system effector protein | A | 8.66 | 6.52 | 1.60E-26 |
| CKS_3258 | HrcJ family type III secretion system component protein | A | 8.65 | 6.42 | 8.16E-23 |
| CKS_2495 | FAD dependent oxidoreductase | A | 8.64 | 6.24 | 2.43E-13 |
| CKS_1361 | PLP-binding diaminopimelate decarboxylase | A | 8.58 | 6.38 | 2.53E-24 |
| CKS_4720 | acetyl-CoA synthetase | A | 8.57 | 6.40 | 6.14E-21 |
| CKS_3038 | iron-uptake factor | A | 8.57 | 6.39 | 1.37E-15 |
| CKS_2371 | hypothetical protein | A | 8.47 | 6.23 | 1.03E-13 |
| CKS_4249 | putative membrane protein | A | 8.46 | 6.08 | 5.04E-12 |
| CKS_2490 | DNA-binding transcriptional dual regulator of nitrogen assimilation | A | 8.41 | 5.53 | 1.13E-05 |
| CKS_4791 | PTS system sucrose-specific IIBC components | A | 8.37 | 6.30 | 4.11E-22 |
| CKS_3860 | molybdopterin biosynthesis protein C | A | 8.33 | 6.26 | 6.60E-20 |
| CKS_0288 | putative permease component of an ABC superfamily amino acid transporter | A | 8.29 | 5.44 | 1.69E-05 |
| CKS_0944 | methylmalonate-semialdehyde dehydrogenase | A | 8.26 | 6.19 | 1.84E-22 |
| CKS_3619 | hypothetical protein | A | 8.23 | 6.19 | 3.19E-20 |
| CKS_3844 | membrane component of an ABC superfamily molybdate transporter | A | 8.21 | 6.19 | 5.71E-26 |
| CKS_3206 | putative amidohydrolase | A | 8.15 | 5.40 | 2.16E-05 |
| CKS_2376 | hypothetical protein | A | 8.13 | 5.95 | 4.92E-14 |
| CKS_4373 | PTS system mannitol-specific EIIABC component | A | 8.13 | 6.03 | 9.32E-16 |
| CKS_2623 | hypothetical protein | A | 8.11 | 6.10 | 5.32E-23 |
| CKS_4763 | 23-diketo-L-gulonate dehydrogenase NADH-dependent | A | 8.09 | 5.91 | 1.05E-14 |
| CKS_4283 | hypothetical protein | A | 8.08 | 5.94 | 1.09E-16 |
| CKS_2922 | fasciclin-like repeat-containing secreted/surface protein | A | 8.08 | 6.05 | 1.51E-22 |
| CKS_0963 | membrane component of an ABC superfamily D-ala-D-ala transporter | A | 7.91 | 5.95 | 1.54E-19 |
| CKS_1227 | ATP-binding component of an ABC superfamily ribose transporter | A | 7.82 | 5.89 | 2.26E-27 |
| CKS_3538 | ATP-binding component of an ABC superfamily L-arabinose transporter | A | 7.80 | 5.85 | 2.28E-24 |
| CKS_3623 | hypothetical protein | A | 7.74 | 5.78 | 3.95E-14 |
| CKS_4800 | predicted dehydrogenase | A | 7.68 | 5.69 | 1.18E-12 |
| CKS_2609 | holliday junction resolvase/crossover junction endodeoxyribonuclease | A | 7.63 | 5.55 | 5.05E-12 |
| CKS_2854 | pyridine nucleotide transhydrogenase alpha subunit | A | 7.61 | 5.61 | 8.60E-10 |
| CKS_5452 | N-acetylmuramoyl-L-alanine amidase | A | 7.61 | 5.63 | 3.75E-15 |
| CKS_2489 | permease component of an ABC superfamily ribose/xylose/arabinose/galactoside transporter | A | 7.54 | 5.69 | 1.35E-22 |
| CKS_5877 | mobilization protein | A | 7.52 | 5.54 | 7.73E-16 |
| CKS_3826 | PhoH family ATPase | A | 7.51 | 5.50 | 6.13E-13 |
| CKS_2493 | aldehyde dehydrogenase | A | 7.50 | 5.42 | 1.58E-09 |
| CKS_4923 | anaerobic ribonucleoside-triphosphate reductase | A | 7.42 | 5.46 | 5.53E-09 |
| CKS_3440 | N-ethylmaleimide reductase FMN-linked | A | 7.39 | 5.60 | 2.50E-23 |
| CKS_2984 | hypothetical protein | A | 7.38 | 5.39 | 7.62E-12 |
| CKS_2625 | hypothetical protein | A | 7.38 | 5.44 | 1.80E-17 |
| CKS_4952 | acetylornithine deacetylase | A | 7.31 | 5.38 | 3.37E-14 |
| CKS_2306 | uncharacterized DUF1537 family protein | A | 7.26 | 5.34 | 3.04E-13 |
| CKS_3838 | Galactokinase | A | 7.23 | 5.39 | 3.55E-17 |
| CKS_4596 | hypothetical protein | A | 7.22 | 5.39 | 6.71E-16 |
| CKS_0908 | Pirin | A | 7.18 | 5.19 | 7.62E-12 |
| CKS_0771 | NAD(P)-binding malate dehydrogenase | A | 7.18 | 5.41 | 2.00E-14 |
| CKS_2803 | hypothetical protein | A | 7.16 | 5.34 | 6.70E-17 |
| CKS_0291 | serine--pyruvate aminotransferase / L-alanine:glyoxylate aminotransferase | A | 7.16 | 4.98 | 3.45E-07 |
| CKS_2641 | phage baseplate assembly protein V | A | 7.15 | 5.32 | 9.23E-14 |
| CKS_1042 | dihydroxyacid dehydratase | A | 7.13 | 5.41 | 1.02E-22 |
| CKS_4597 | type III secretion system inner membrane protein | A | 7.11 | 5.16 | 1.36E-08 |
| CKS_3837 | galactose-1-epimerase (mutarotase) | A | 7.05 | 5.31 | 2.00E-13 |
| CKS_3549 | Oxidoreductase | A | 7.03 | 5.17 | 8.52E-12 |
| CKS_2165 | PTS system fructose-specific IIB'BC component | A | 7.02 | 5.31 | 6.15E-23 |
| CKS_3622 | pyruvate/alpha-keto-acid decarboxylase | A | 6.99 | 5.28 | 9.03E-21 |
| CKS_4760 | permease component of an ABC superfamily ribose transporter | A | 6.99 | 5.25 | 1.86E-19 |
| CKS_1045 | acetolactate synthase large subunit | A | 6.98 | 5.28 | 1.08E-20 |
| CKS_2792 | aconitate hydratase 1 | A | 6.92 | 5.23 | 2.69E-20 |
| CKS_0370 | hypothetical protein | A | 6.90 | 5.22 | 3.64E-19 |
| CKS_2525 | permease component of an ABC superfamily ribose transporter | A | 6.88 | 5.05 | 7.32E-10 |
| CKS_0371 | hypothetical protein | A | 6.81 | 5.17 | 7.03E-23 |
| CKS_0657 | altronate hydrolase | A | 6.80 | 5.09 | 4.82E-16 |
| CKS_5309 | DNA polymerase V subunit D | A | 6.79 | 4.95 | 5.25E-08 |
| CKS_2492 | succinate-semialdehyde dehydrogenase I NADP-dependent | A | 6.77 | 4.92 | 3.75E-11 |
| CKS_2222 | predicted peptidase | A | 6.75 | 5.05 | 4.84E-10 |
| CKS_0965 | ATP-binding component of an ABC superfamily D-ala-D-ala transporter | A | 6.75 | 5.09 | 2.36E-18 |
| CKS_3739 | membrane component of an ABC superfamily glutamate and aspartate transporter | A | 6.69 | 5.07 | 1.30E-20 |
| CKS_0922 | periplasmic-binding component of an ABC superfamily leucine transporter | A | 6.69 | 5.05 | 6.10E-15 |
| CKS_3984 | pyruvate formate-lyase | A | 6.68 | 4.83 | 9.76E-06 |
| CKS_3859 | molybdopterin biosynthesis protein B | A | 6.65 | 5.00 | 9.19E-15 |
| CKS_2626 | hypothetical protein | A | 6.64 | 5.01 | 4.90E-19 |
| CKS_2530 | putative response regulator protein | A | 6.61 | 4.91 | 4.64E-17 |
| CKS_2629 | phage tail sheath protein FI | A | 6.56 | 4.92 | 1.29E-14 |
| CKS_4762 | gluconolactonase | A | 6.54 | 4.94 | 9.74E-21 |
| CKS_2642 | hypothetical protein | A | 6.54 | 4.83 | 2.27E-13 |
| CKS_5411 | Endonuclease | A | 6.50 | 4.87 | 7.16E-17 |
| CKS_4584 | putative type III secretion system apparatus protein | A | 6.50 | 4.91 | 6.17E-21 |
| CKS_0939 | epi-inositol hydrolase | A | 6.47 | 4.86 | 1.51E-13 |
| CKS_3703 | L-fucose operon activator | A | 6.46 | 4.61 | 1.93E-06 |
| CKS_2425 | Transketolase | A | 6.46 | 4.72 | 6.34E-08 |
| CKS_0658 | altronate oxidoreductase NAD-dependent | A | 6.43 | 4.83 | 1.50E-16 |
| CKS_0204 | ammonium transporter | A | 6.42 | 3.10 | 1.02E-01 |
| CKS_2681 | PTS system mannose-specific IIAB component | A | 6.41 | 4.79 | 1.91E-11 |
| CKS_2378 | hypothetical protein | A | 6.37 | 4.73 | 2.58E-12 |
| CKS_4332 | PTS system cellobiose-specific IIC component | A | 6.35 | 4.77 | 1.77E-14 |
| CKS_4046 | DNA-binding protein hemimethylated | A | 6.34 | 4.53 | 4.04E-07 |
| CKS_2744 | alcohol/acetaldehyde-CoA dehydrogenase | A | 6.34 | 4.76 | 1.14E-09 |
| CKS_4280 | hypothetical protein | A | 6.32 | 4.78 | 1.03E-20 |
| CKS_2622 | hypothetical protein | A | 6.31 | 4.76 | 1.60E-16 |
| CKS_4020 | periplasmic-binding component of an ABC superfamily alkanesulfonate transporter | A | 6.26 | 4.31 | 4.88E-05 |
| CKS_5453 | hypothetical protein | A | 6.25 | 4.61 | 1.04E-10 |
| CKS_0592 | hypothetical protein | A | 6.24 | 4.71 | 9.97E-18 |
| CKS_2587 | hypothetical protein | A | 6.21 | 4.32 | 3.61E-05 |
| CKS_1762 | drug/metabolite transporter (DMT) superfamily permease | A | 6.19 | 4.60 | 8.99E-12 |
| CKS_4390 | membrane component of an ABC superfamily dipeptide transporter | A | 6.18 | 4.62 | 2.09E-10 |
| CKS_2386 | hypothetical protein | A | 6.16 | 4.59 | 1.05E-10 |
| CKS_4389 | membrane component of an ABC superfamily dipeptide transporter | A | 6.16 | 4.60 | 3.98E-10 |
| CKS_5394 | putative inner membrane protein | A | 6.13 | 4.61 | 3.03E-13 |
| CKS_3243 | hypothetical protein | A | 6.13 | 4.64 | 4.51E-16 |
| CKS_4593 | hypothetical protein | A | 6.07 | 4.55 | 3.43E-17 |
| CKS_5456 | putative inner membrane protein | A | 6.04 | 4.55 | 6.40E-13 |
| CKS_1836 | copper homeostasis protein | A | 6.03 | 4.49 | 3.90E-15 |
| CKS_5350 | putative inner membrane protein | A | 6.02 | 4.54 | 4.47E-13 |
| CKS_0468 | hypothetical protein | A | 5.98 | 4.48 | 1.78E-09 |
| CKS_0452 | putative peptidase | A | 5.95 | 4.51 | 3.56E-15 |
| CKS_0791 | predicted reductase | A | 5.95 | 4.41 | 3.77E-11 |
| CKS_2679 | mannose-specific enzyme IID component of PTS | A | 5.94 | 4.47 | 1.77E-11 |
| CKS_4594 | hypothetical protein | A | 5.93 | 4.47 | 1.00E-14 |
| CKS_3146 | dihydrodipicolinate synthase | A | 5.93 | 4.38 | 1.39E-08 |
| CKS_4754 | outer membrane glucose/carbohydrate porin | A | 5.92 | 4.50 | 8.72E-23 |
| CKS_2590 | hypothetical protein | A | 5.92 | 4.07 | 1.66E-04 |
| CKS_5026 | hypothetical protein | A | 5.91 | 4.09 | 1.11E-04 |
| CKS_3535 | L-arabinose isomerase | A | 5.89 | 4.37 | 1.06E-12 |
| CKS_4583 | secretion system apparatus protein | A | 5.88 | 4.45 | 2.71E-16 |
| CKS_2921 | putative RNA polymerase sigma factor | A | 5.87 | 4.43 | 5.39E-20 |
| CKS_0920 | membrane component of an ABC superfamily leucine/isoleucine/valine transporter | A | 5.86 | 4.41 | 4.10E-13 |
| CKS_5412 | putative DSBA oxidoreductase | A | 5.86 | 4.44 | 3.17E-12 |
| CKS_2588 | hypothetical protein | A | 5.85 | 4.01 | 3.03E-04 |
| CKS_2920 | hypothetical protein | A | 5.85 | 4.44 | 1.99E-16 |
| CKS_5152 | 2-isopropylmalate synthase | A | 5.83 | 4.43 | 3.41E-21 |
| CKS_2628 | hypothetical protein | A | 5.83 | 4.35 | 1.57E-13 |
| CKS_0290 | putative ATP-binding component of an ABC superfamily amino acid transporter | A | 5.83 | 4.01 | 1.52E-04 |
| CKS_2800 | L-ribulose-5-phosphate 4-epimerase | A | 5.80 | 4.37 | 3.67E-16 |
| CKS_3982 | predicted transporter | A | 5.77 | 4.37 | 3.84E-21 |
| CKS_4764 | hypothetical protein | A | 5.77 | 4.26 | 1.78E-08 |
| CKS_2924 | metal-activated pyridoxal enzyme | A | 5.75 | 4.32 | 7.89E-13 |
| CKS_1226 | predicted cytoplasmic sugar-binding protein | A | 5.71 | 4.26 | 1.62E-14 |
| CKS_0551 | carbonic anhydrase | A | 5.70 | 4.10 | 6.47E-08 |
| CKS_2635 | hypothetical protein | A | 5.69 | 4.00 | 1.25E-05 |
| CKS_2305 | NAD-binding dehydrogenase | A | 5.67 | 4.25 | 2.38E-13 |
| CKS_4578 | secretion system effector protein | A | 5.67 | 4.27 | 2.79E-16 |
| CKS_4272 | putative DNA modification methylase | A | 5.65 | 4.25 | 5.36E-14 |
| CKS_2387 | hypothetical protein | A | 5.64 | 4.20 | 4.58E-08 |
| CKS_2496 | extracellular solute-binding protein family 5 | A | 5.57 | 4.13 | 1.30E-10 |
| CKS_2705 | D-amino acid dehydrogenase small subunit | A | 5.56 | 4.23 | 1.69E-17 |
| CKS_4592 | putative type III secretion system ATP synthase | A | 5.53 | 4.21 | 1.47E-15 |
| CKS_2883 | predicted dethiobiotin synthetase | A | 5.53 | 3.25 | 5.97E-02 |
| CKS_4391 | ATP-binding component of an ABC superfamily dipeptide transporter | A | 5.53 | 4.17 | 9.56E-10 |
| CKS_3738 | permease component of an ABC superfamily glutamate/aspartate transporter | A | 5.51 | 4.16 | 1.43E-15 |
| CKS_2550 | DgsA-binding anti-repressor | A | 5.49 | 4.07 | 4.51E-09 |
| CKS_4922 | anaerobic ribonucleotide reductase activating protein | A | 5.48 | 4.10 | 3.27E-07 |
| CKS_0340 | dihydroxyacetone kinase subunit | A | 5.47 | 4.10 | 4.17E-09 |
| CKS_2062 | permease component of an ABC superfamily spermidine/putrescine transporter | A | 5.47 | 4.13 | 1.24E-12 |
| CKS_0429 | hypothetical protein | A | 5.47 | 4.15 | 6.31E-14 |
| CKS_3209 | predicted oxidoreductase | A | 5.46 | 3.95 | 8.57E-06 |
| CKS_5620 | partitioning protein SpyA | A | 5.46 | 3.99 | 2.96E-07 |
| CKS_2202 | hypothetical protein | A | 5.45 | 3.92 | 5.09E-04 |
| CKS_3534 | membrane-fusion protein | A | 5.42 | 3.90 | 5.28E-06 |
| CKS_3843 | periplasmic substrate-binding component of an ABC superfamily molybdate transporter | A | 5.41 | 4.09 | 1.70E-17 |
| CKS_2637 | hypothetical protein | A | 5.41 | 4.07 | 3.38E-15 |
| CKS_3137 | hypothetical protein | A | 5.38 | 3.99 | 1.37E-08 |
| CKS_2012 | long-chain fatty acid outer membrane transporter | A | 5.36 | 4.02 | 5.71E-13 |
| CKS_0921 | membrane component of an ABC superfamily leucine/isoleucine/valine transporter | A | 5.36 | 4.05 | 3.28E-12 |
| CKS_4703 | DNA-binding transcriptional dual regulator Fe-S center for redox-sensing | A | 5.34 | 3.95 | 4.24E-09 |
| CKS_3147 | alcohol dehydrogenase | A | 5.33 | 3.96 | 2.16E-08 |
| CKS_2640 | hypothetical protein | A | 5.32 | 3.99 | 3.74E-12 |
| CKS_5481 | mobilization protein | A | 5.32 | 3.97 | 3.65E-11 |
| CKS_3861 | molybdopterin synthase small subunit | A | 5.32 | 4.02 | 2.30E-12 |
| CKS_4766 | L-xylulose kinase | A | 5.30 | 3.93 | 7.58E-10 |
| CKS_3271 | type III secretion system outermembrane pore forming protein | A | 5.29 | 4.02 | 1.81E-13 |
| CKS_2423 | membrane-bound lytic murein transglycosylase E | A | 5.29 | 4.00 | 2.73E-16 |
| CKS_5477 | mobilization protein | A | 5.28 | 3.94 | 1.07E-10 |
| CKS_4591 | secretion system apparatus protein | A | 5.26 | 4.00 | 4.67E-17 |
| CKS_5064 | hexuronate transporter | A | 5.25 | 3.90 | 1.50E-09 |
| CKS_1090 | glycerol kinase | A | 5.23 | 3.90 | 1.05E-11 |
| CKS_3552 | mannose-6-phosphate isomerase | A | 5.22 | 3.94 | 2.38E-09 |
| CKS_3503 | methyl-accepting chemotaxis protein | A | 5.21 | 3.97 | 5.82E-14 |
| CKS_2644 | hypothetical protein | A | 5.21 | 3.85 | 4.58E-08 |
| CKS_3255 | type III secretion system cytoplasmic ATP synthase | A | 5.20 | 3.95 | 1.24E-12 |
| CKS_4340 | heat shock chaperone | A | 5.17 | 3.65 | 5.95E-05 |
| CKS_0659 | uronate isomerase | A | 5.15 | 3.89 | 1.03E-15 |
| CKS_2753 | ATP-binding component of an ABC superfamily oligopeptide transporter | A | 5.13 | 3.88 | 5.47E-09 |
| CKS_0266 | cytosine/purine/uracil/thiamine/allantoin permease family protein | A | 5.12 | 2.61 | 1.82E-01 |
| CKS_2368 | gp55 family protein | A | 5.10 | 3.79 | 1.37E-08 |
| CKS_0874 | phosphoenolpyruvate carboxykinase | A | 5.07 | 3.85 | 2.35E-17 |
| CKS_5356 | putative outer membrane adhesion protein | A | 5.06 | 3.86 | 2.49E-17 |
| CKS_5151 | 3-isopropylmalate dehydrogenase | A | 5.06 | 3.85 | 1.36E-14 |
| CKS_5834 | hypothetical protein | A | 5.03 | 3.58 | 1.36E-04 |
| CKS_4718 | acetate permease | A | 5.03 | 3.79 | 2.04E-08 |
| CKS_2563 | putative mannosyl-3-phosphoglycerate phosphatase | A | 5.03 | 3.78 | 3.04E-09 |
| CKS_5404 | putative outer membrane adhesion protein | A | 5.03 | 3.83 | 2.35E-13 |
| CKS_2592 | hypothetical protein | A | 4.99 | 3.51 | 6.12E-04 |
| CKS_3268 | HrpD family protein | A | 4.97 | 3.71 | 6.23E-09 |
| CKS_5754 | hypothetical protein | A | 4.96 | 3.50 | 2.24E-04 |
| CKS_3620 | hypothetical protein | A | 4.95 | 3.72 | 6.02E-10 |
| CKS_1696 | magnesium transporter | A | 4.93 | 3.75 | 1.44E-09 |
| CKS_3858 | molybdopterin biosynthesis protein A | A | 4.92 | 3.75 | 7.23E-12 |
| CKS_0571 | Phosphopentomutase | A | 4.92 | 3.76 | 8.75E-16 |
| CKS_4484 | 3-ketoacyl-CoA thiolase (thiolase I) | A | 4.91 | 3.69 | 5.80E-15 |
| CKS_2880 | predicted transporter | A | 4.91 | 3.72 | 4.10E-13 |
| CKS_2624 | hypothetical protein | A | 4.86 | 3.68 | 1.99E-13 |
| CKS_3983 | pyruvate formate lyase activating enzyme 1 | A | 4.85 | 3.65 | 9.35E-06 |
| CKS_4056 | WrbA family flavoprotein | A | 4.85 | 3.67 | 9.41E-08 |
| CKS_5446 | hypothetical protein | A | 4.85 | 3.65 | 3.04E-14 |
| CKS_3094 | GAF domain/GGDEF domain/EAL domain protein | A | 4.84 | 3.55 | 9.00E-06 |
| CKS_4794 | beta-D-galactosidase | A | 4.83 | 3.68 | 1.91E-12 |
| CKS_4942 | ornithine carbamoyltransferase 1 | A | 4.83 | 3.67 | 3.11E-17 |
| CKS_2589 | phage-related protein | A | 4.82 | 3.39 | 8.85E-04 |
| CKS_4712 | hypothetical protein | A | 4.82 | 3.63 | 2.78E-14 |
| CKS_3714 | gluconate transporter high-affinity GNT I system | A | 4.81 | 3.63 | 4.35E-08 |
| CKS_2494 | type II haloacid dehalogenase | A | 4.81 | 3.56 | 1.51E-06 |
| CKS_5310 | DNA polymerase V subunit C | A | 4.79 | 3.61 | 5.84E-10 |
| CKS_1538 | hypothetical protein | A | 4.78 | 3.64 | 1.65E-09 |
| CKS_2385 | hypothetical protein | A | 4.77 | 3.52 | 6.93E-07 |
| CKS_3278 | methyl-accepting chemotaxis sensory transducer | A | 4.77 | 3.59 | 1.43E-11 |
| CKS_5439 | hypothetical protein | A | 4.76 | 3.51 | 1.83E-07 |
| CKS_0191 | L36 family ribosomal protein | A | 4.76 | 3.51 | 4.85E-08 |
| CKS_3385 | predicted phosphotransferase/kinase | A | 4.75 | 3.62 | 1.42E-12 |
| CKS_5025 | phage-related protein | A | 4.74 | 3.34 | 8.63E-04 |
| CKS_4021 | NAD(P)H-dependent FMN reductase | A | 4.73 | 3.20 | 4.53E-03 |
| CKS_0561 | methyl-accepting chemotaxis protein | A | 4.73 | 3.53 | 3.26E-10 |
| CKS_4590 | secretion system apparatus protein | A | 4.73 | 3.57 | 2.84E-12 |
| CKS_4571 | virulence protein | A | 4.72 | 3.58 | 5.52E-14 |
| CKS_2947 | maltose regulon periplasmic protein | A | 4.71 | 3.54 | 4.05E-09 |
| CKS_0943 | 5-deoxy-glucuronate isomerase | A | 4.71 | 3.59 | 1.12E-12 |
| CKS_4742 | ferrous iron uptake protein | A | 4.70 | 3.57 | 1.68E-07 |
| CKS_2693 | acyl-CoA synthetase long-chain-fatty-acid--CoA ligase | A | 4.70 | 3.56 | 4.26E-16 |
| CKS_0912 | hypothetical protein | A | 4.69 | 3.49 | 1.94E-09 |
| CKS_0640 | zinc-containing alcohol dehydrogenase/quinone oxidoreductase | A | 4.68 | 3.56 | 1.96E-10 |
| CKS_1810 | uncharacterized DUF533 family protein | A | 4.67 | 3.48 | 9.72E-08 |
| CKS_4162 | hypothetical protein | A | 4.64 | 3.32 | 3.31E-04 |
| CKS_3536 | L-ribulokinase | A | 4.63 | 3.47 | 1.96E-08 |
| CKS_1698 | hypothetical protein | A | 4.62 | 3.53 | 2.19E-10 |
| CKS_2594 | hypothetical protein | A | 4.60 | 3.24 | 1.55E-03 |
| CKS_4512 | putative inner membrane protein | A | 4.59 | 3.51 | 4.28E-12 |
| CKS_1697 | divalent cation transport protein | A | 4.57 | 3.49 | 3.94E-11 |
| CKS_0941 | inosose dehydratase | A | 4.56 | 3.40 | 2.89E-07 |
| CKS_5024 | hypothetical protein | A | 4.55 | 3.20 | 1.54E-03 |
| CKS_0569 | 2-deoxyribose-5-phosphate aldolase NAD(P)-linked | A | 4.54 | 3.46 | 1.26E-12 |
| CKS_0292 | N-carbamoyl-L-amino acid hydrolase | A | 4.54 | 3.35 | 5.50E-06 |
| CKS_4462 | HD superfamily hydrolase | A | 4.53 | 3.43 | 3.83E-07 |
| CKS_5352 | outer membrane adhesion/aggregation protein | A | 4.51 | 3.44 | 4.11E-14 |
| CKS_0412 | hypothetical protein | A | 4.51 | 3.34 | 5.94E-07 |
| CKS_2983 | phytochelatin synthase | A | 4.51 | 3.40 | 2.70E-11 |
| CKS_1228 | membrane component of an ABC superfamily D-ribose transporter | A | 4.50 | 3.42 | 4.71E-11 |
| CKS_3273 | HrpT family protein | A | 4.48 | 3.39 | 1.28E-08 |
| CKS_2377 | hypothetical protein | A | 4.46 | 3.36 | 3.44E-10 |
| CKS_2593 | single-stranded DNA-binding protein | A | 4.45 | 3.16 | 1.59E-03 |
| CKS_5480 | MbeA family protein | A | 4.45 | 3.38 | 1.02E-13 |
| CKS_0476 | 3-oxoadipate CoA-transferase subunit B | A | 4.45 | 3.26 | 6.93E-05 |
| CKS_0287 | putative periplasmic substrate-binding component of an ABC superfamily amino acid transporter | A | 4.45 | 3.07 | 3.43E-03 |
| CKS_2706 | PLP-binding alanine racemase 2 | A | 4.43 | 3.37 | 5.22E-10 |
| CKS_3409 | predicted inner membrane protein | A | 4.41 | 3.37 | 5.62E-10 |
| CKS_3241 | amidinotransferase family protein | A | 4.40 | 3.30 | 2.28E-09 |
| CKS_2524 | ATP-binding component of an ABC superfamily ribose transporter | A | 4.40 | 3.31 | 6.78E-08 |
| CKS_3276 | putative avirulence protein | A | 4.39 | 3.31 | 2.66E-10 |
| CKS_3825 | uncharacterized UPF0054 family protein | A | 4.38 | 3.28 | 3.95E-09 |
| CKS_2627 | hypothetical protein | A | 4.34 | 3.27 | 1.30E-10 |
| CKS_3256 | type III secretion system protein | A | 4.33 | 3.29 | 1.70E-09 |
| CKS_3702 | periplasmic substrate binding component of an ABC superfamily sugar transporter | A | 4.33 | 3.19 | 9.16E-05 |
| CKS_2752 | membrane component of an ABC superfamily oligopeptide transporter | A | 4.33 | 3.27 | 1.78E-06 |
| CKS_3150 | predicted mannonate dehydrogenase | A | 4.32 | 3.28 | 2.11E-05 |
| CKS_3127 | hypothetical protein | A | 4.32 | 3.28 | 2.27E-07 |
| CKS_5250 | serine endoprotease (protease Do) membrane-associated | A | 4.30 | 3.26 | 6.31E-09 |
| CKS_4572 | hypothetical protein | A | 4.29 | 3.20 | 5.44E-07 |
| CKS_0540 | site-specific recombinase phage integrase family | A | 4.29 | 3.19 | 3.41E-05 |
| CKS_4208 | FlgF family flagellar basal-body rod protein | A | 4.28 | 3.15 | 2.10E-04 |
| CKS_4958 | putative binding periplasmic protein of ABC transporter | A | 4.28 | 3.26 | 4.96E-11 |
| CKS_2106 | hypothetical protein | A | 4.27 | 3.23 | 8.26E-07 |
| CKS_5716 | hypothetical protein | A | 4.27 | 2.99 | 3.27E-03 |
| CKS_1191 | D-xylose isomerase | A | 4.26 | 3.21 | 2.18E-09 |
| CKS_3257 | type III secretion system inner membrane channel protein | A | 4.25 | 3.23 | 2.76E-10 |
| CKS_2307 | predicted class II aldolase | A | 4.22 | 3.19 | 8.40E-07 |
| CKS_2441 | methyltransferase | A | 4.22 | 3.11 | 2.77E-05 |
| CKS_3596 | putative acetyltransferase | A | 4.22 | 3.15 | 1.55E-07 |
| CKS_3202 | ABC superfamily transporter | A | 4.21 | 3.19 | 4.31E-08 |
| CKS_5183 | GMP reductase | A | 4.21 | 3.22 | 5.36E-09 |
| CKS_0469 | 4-hydroxybenzoate transporter | A | 4.20 | 3.15 | 4.15E-08 |
| CKS_4273 | hypothetical protein | A | 4.20 | 3.16 | 1.71E-07 |
| CKS_3862 | molybdopterin synthase large subunit | A | 4.20 | 3.19 | 2.88E-09 |
| CKS_5017 | hypothetical protein | A | 4.19 | 2.35 | 2.30E-01 |
| CKS_4163 | hypothetical protein | A | 4.15 | 3.12 | 1.19E-07 |
| CKS_5419 | hypothetical protein | A | 4.15 | 3.12 | 4.91E-09 |
| CKS_2361 | DNA adenine methylase | A | 4.14 | 3.13 | 1.99E-07 |
| CKS_4523 | type III secretion system peptide export protein | A | 4.14 | 3.15 | 5.80E-13 |
| CKS_2639 | hypothetical protein | A | 4.13 | 3.11 | 8.86E-09 |
| CKS_3593 | hypothetical protein | A | 4.13 | 3.14 | 2.33E-08 |
| CKS_1043 | branched-chain amino-acid aminotransferase | A | 4.12 | 3.15 | 7.35E-09 |
| CKS_0328 | transcriptional activator-regulatory protein | A | 4.12 | 3.15 | 8.99E-12 |
| CKS_1541 | IMP dehydrogenase | A | 4.12 | 3.14 | 1.04E-09 |
| CKS_3024 | fumarase A | A | 4.10 | 3.14 | 2.18E-09 |
| CKS_3839 | galactose-1-phosphate uridylyltransferase | A | 4.09 | 3.11 | 4.80E-09 |
| CKS_0440 | hypothetical protein | A | 4.09 | 3.09 | 4.16E-11 |
| CKS_0312 | hypothetical protein | A | 4.09 | 3.10 | 1.06E-09 |
| CKS_0477 | 3-oxoadipate CoA-transferase subunit A | A | 4.07 | 2.99 | 1.30E-04 |
| CKS_1444 | GroES family alcohol dehydrogenase | A | 4.07 | 3.11 | 7.16E-08 |
| CKS_3845 | ATP-binding component of an ABC superfamily molybdate transporter | A | 4.07 | 3.11 | 1.47E-08 |
| CKS_5149 | 3-isopropylmalate isomerase subunit | A | 4.07 | 3.11 | 4.82E-07 |
| CKS_4801 | putative polygalacturonase protein | A | 4.05 | 3.07 | 4.12E-07 |
| CKS_1988 | phage-related protein | A | 4.05 | 3.03 | 4.14E-05 |
| CKS_3469 | glycogen debranching enzyme | A | 4.04 | 3.07 | 1.38E-06 |
| CKS_3872 | UDP-galactose-lipid carrier transferase | A | 4.03 | 3.07 | 1.62E-09 |
| CKS_3627 | GCN5 family N-acetyltransferase | A | 4.03 | 3.08 | 1.98E-08 |
| CKS_3210 | predicted oxidoreductase flavin:NADH component | A | 4.03 | 2.90 | 2.99E-03 |
| CKS_2603 | hypothetical protein | A | 4.02 | 2.98 | 2.55E-04 |
| CKS_2369 | hypothetical protein | A | 4.02 | 2.95 | 1.75E-04 |
| CKS_2754 | ATP-binding subunit of oligopeptide ABC transporter | A | 4.02 | 3.07 | 9.85E-07 |
| CKS_3143 | hypothetical protein | A | 4.01 | 3.03 | 1.50E-05 |
| CKS_0272 | phytanoyl-CoA dioxygenase family protein | A | 4.01 | 2.99 | 3.85E-04 |
| CKS_2591 | hypothetical protein | A | 4.01 | 2.84 | 6.09E-03 |
| CKS_4095 | hypothetical protein | R | 4.03 | 5.08 | 2.08E-15 |
| CKS_1527 | nucleoside diphosphate kinase | R | 4.06 | 5.10 | 1.83E-14 |
| CKS_4091 | hypothetical protein | R | 4.06 | 5.09 | 2.85E-17 |
| CKS_4133 | putative bacteriophage protein | R | 4.06 | 5.08 | 2.65E-18 |
| CKS_4368 | superoxide dismutase Mn | R | 4.10 | 5.21 | 2.20E-18 |
| CKS_4082 | hypothetical protein | R | 4.10 | 5.02 | 4.62E-09 |
| CKS_5095 | Trp operon repressor | R | 4.16 | 5.28 | 5.53E-27 |
| CKS_5071 | uncharacterized DUF1328 family protein | R | 4.31 | 5.44 | 3.22E-28 |
| CKS_4088 | hypothetical protein | R | 4.33 | 5.31 | 8.01E-11 |
| CKS_4776 | IucA family aerobactin siderophore biosynthesis protein | R | 4.36 | 5.53 | 2.65E-17 |
| CKS_5576 | hypothetical protein | R | 4.48 | 5.60 | 6.54E-13 |
| CKS_0178 | adenylate kinase | R | 4.51 | 5.66 | 4.67E-17 |
| CKS_0117 | peptidyl-prolyl cis-trans isomerase B (rotamase B) | R | 4.56 | 5.75 | 6.60E-20 |
| CKS_0744 | 30S ribosomal subunit protein S6 | R | 4.60 | 5.83 | 3.93E-19 |
| CKS_4081 | hypothetical protein | R | 4.61 | 5.69 | 8.96E-12 |
| CKS_0004 | HU DNA-binding transcriptional regulator alpha subunit | R | 4.71 | 5.93 | 7.63E-19 |
| CKS_3122 | DNA-binding protein H-NS | R | 4.75 | 6.01 | 1.33E-19 |
| CKS_0733 | FKBP-type peptidyl-prolyl cis-trans isomerase (rotamase) | R | 4.83 | 6.13 | 6.64E-25 |
| CKS_4137 | hypothetical protein | R | 4.87 | 6.13 | 2.87E-20 |
| CKS_1116 | ADP-heptose--lipooligosaccharide heptosyltransferase II | R | 4.88 | 6.16 | 4.11E-20 |
| CKS_0742 | 30S ribosomal subunit protein S18 | R | 4.91 | 6.20 | 5.73E-19 |
| CKS_4089 | hypothetical protein | R | 4.95 | 6.18 | 6.42E-17 |
| CKS_0010 | thiamin (pyrimidine moiety) biosynthesis protein | R | 4.96 | 6.21 | 2.96E-27 |
| CKS_4127 | putative bacteriophage protein | R | 4.99 | 6.28 | 4.66E-25 |
| CKS_4084 | phage lysozyme | R | 5.00 | 6.10 | 1.66E-12 |
| CKS_5198 | dihydrolipoamide dehydrogenase | R | 5.03 | 6.39 | 3.80E-34 |
| CKS_4530 | type III secretion system cytoplasmic ATP synthase | R | 5.03 | 6.26 | 4.39E-26 |
| CKS_4132 | phage-related protein | R | 5.12 | 6.47 | 4.94E-31 |
| CKS_0644 | diaminobutyrate-pyruvate transaminase/L-24-diaminobutyrate decarboxylase | R | 5.23 | 6.52 | 4.64E-17 |
| CKS_4085 | hypothetical protein | R | 5.25 | 6.44 | 7.57E-15 |
| CKS_4125 | putative bacteriophage protein | R | 5.26 | 6.64 | 2.65E-35 |
| CKS_4123 | putative bacteriophage protein | R | 5.31 | 6.65 | 4.80E-29 |
| CKS_0741 | 50S ribosomal subunit protein L9 | R | 5.37 | 6.77 | 1.59E-20 |
| CKS_5070 | periplasmic protein | R | 5.41 | 6.86 | 1.04E-36 |
| CKS_4093 | CP4-57 family phage integrase | R | 5.42 | 6.79 | 8.33E-21 |
| CKS_0743 | primosomal replication protein N | R | 5.48 | 6.87 | 4.09E-18 |
| CKS_4126 | putative bacteriophage protein | R | 5.56 | 7.00 | 4.37E-31 |
| CKS_4109 | putative bacteriophage protein | R | 5.60 | 7.00 | 8.76E-30 |
| CKS_4092 | hypothetical protein | R | 5.84 | 7.37 | 8.32E-36 |
| CKS_1031 | malate:quinone oxidoreductase | R | 5.95 | 7.20 | 1.24E-13 |
| CKS_3057 | ThiS family thiamine biosynthesis protein | R | 6.04 | 7.48 | 2.71E-26 |
| CKS_4122 | hypothetical protein | R | 6.27 | 7.78 | 1.05E-28 |
| CKS_5211 | putative alpha/beta superfamily hydrolase/acyltransferase | R | 6.28 | 7.92 | 5.87E-31 |
| CKS_4090 | hypothetical protein | R | 6.30 | 7.91 | 1.55E-28 |
| CKS_4083 | hypothetical protein | R | 6.36 | 7.83 | 7.74E-20 |
| CKS_5188 | quinolinate phosphoribosyltransferase | R | 6.38 | 7.98 | 2.21E-36 |
| CKS_1241 | 3-deoxy-D-arabino-heptulosonate-7-phosphate synthase tyrosine-repressible | R | 6.41 | 7.62 | 4.90E-11 |
| CKS_4136 | putative bacteriophage tail protein | R | 6.45 | 8.14 | 8.79E-38 |
| CKS_4717 | putative exported protein | R | 6.62 | 8.31 | 3.46E-25 |
| CKS_4115 | putative bacteriophage protein | R | 6.74 | 8.42 | 3.22E-38 |
| CKS_2902 | short chain dehydrogenase,putative oxidoreductase | R | 6.88 | 8.55 | 9.89E-35 |
| CKS_4134 | putative bacteriophage protein | R | 6.95 | 8.74 | 2.69E-44 |
| CKS_4087 | hypothetical protein | R | 6.99 | 8.62 | 8.50E-21 |
| CKS_3613 | quinolinate phosphoribosyltransferase | R | 7.07 | 8.84 | 5.44E-41 |
| CKS_0011 | thiamin phosphate synthase (thiamin phosphate pyrophosphorylase) | R | 7.10 | 8.91 | 4.23E-40 |
| CKS_4121 | putative bacteriophage protein | R | 7.29 | 9.14 | 6.91E-43 |
| CKS_2885 | putative binding-protein-dependent transport system component | R | 7.31 | 9.14 | 1.35E-41 |
| CKS_4117 | putative bacteriophage protein | R | 7.45 | 9.15 | 1.12E-31 |
| CKS_4114 | putative bacteriophage protein | R | 7.69 | 9.59 | 5.69E-41 |
| CKS_4116 | putative bacteriophage protein | R | 7.72 | 9.62 | 1.96E-39 |
| CKS_4135 | putative bacteriophage protein | R | 7.93 | 9.91 | 9.12E-35 |
| CKS_1399 | component of the MscS mechanosensitive channel | R | 8.06 | 10.13 | 1.01E-54 |
| CKS_4124 | hypothetical protein | R | 8.12 | 10.11 | 1.49E-35 |
| CKS_4535 | type III secretion system regulatory protein | R | 8.39 | 10.35 | 3.11E-25 |
| CKS_4721 | glutamate/aspartate:proton symporter | R | 8.48 | 10.56 | 1.73E-29 |
| CKS_4120 | putative bacteriophage protein | R | 8.63 | 10.85 | 1.14E-50 |
| CKS_0950 | hypothetical protein | R | 8.64 | 10.82 | 2.13E-41 |
| CKS_4113 | hypothetical protein | R | 8.85 | 11.12 | 1.12E-46 |
| CKS_3058 | glycine oxidase | R | 9.22 | 11.55 | 1.49E-46 |
| CKS_2887 | putative membrane component of an ABC superfamily transporter | R | 9.33 | 11.50 | 2.15E-34 |
| CKS_4118 | putative bacteriophage protein | R | 10.71 | 13.17 | 9.23E-44 |
| CKS_4119 | putative bacteriophage protein | R | 10.89 | 13.60 | 2.68E-58 |
| CKS_2886 | periplasmic substrate-binding component of an ABC superfamily glycine/betaine transporter | R | 10.96 | 13.74 | 9.75E-61 |
| CKS_4536 | type III secretion system effector protein | R | 11.16 | 13.91 | 3.06E-45 |
| CKS_4531 | type III secretion system apparatus protein | R | 14.13 | 17.42 | 1.55E-68 |
| CKS_4538 | type III secretion system apparatus protein | R | 15.91 | 19.65 | 1.17E-59 |
| CKS_1225 | hypothetical protein | R | 23.57 | 28.75 | 5.57E-63 |
| CKS_4537 | type III secretion system effector protein | R | 36.62 | 44.52 | 1.97E-89 |

^a^A = activated in the *in planta* culture compared to *in vitro* plate culture (lower in plate culture), R = repressed in the *in planta* culture compared to *in vitro* plate culture (higher in plate culture)
