# Supplementary material for: Analysis of the in planta transcriptome expressed by the corn pathogen Pantoea stewartii subsp. stewartii via RNA-Seq
Source: PeerJ. 2017 Apr 27;5:e3237. doi: 10.7717/peerj.3237 (PMC5410145; doi:10.7717/peerj.3237)
Supplement: Table S4 — a Primers listed as coding DNA sequence (CDS) were for the cloning of each gene into pGEM-T, and RT was for the qRT-PCR protocol. [file peerj-05-3237-s004.docx]

**Table S4.** Primers designed for the genes of interest selected for cloning and qRT-PCR^a^

| **Gene** | **5’-3’ Primer Sequence** | **Function (Annealing Temperature °C)** |
| --- | --- | --- |
| CKS_3263 CDS | F: CAGAACTGAATGGCTTTTGC  R: GGACTTGGTGGTCCACT | Cloning CKS_3263 coding region |
| CKS_3263 RT | F: TATGACCGGAGTTGATTTCTTTGG  R: TGCCCAGGCAGCTAAAATGA | qRT-PCR (60°C) |
| CKS_3793 CDS | F: ATGCTAGATATCGTCGAACTGTC  R: TCTGTTCATGGTGATAGCGC | Cloning CKS_3793 coding region (Kernell Burke et al., 2015) |
| CKS_3793 RT | F: CCTTTGTGGGCCTGTTCTTTTT  R: ACCGCCAGATGCTGCACTT | qRT-PCR (64°C) (Kernell Burke et al., 2015) |
| *rmf* CDS | F: ATGAAGAGACAGAAACGAGACC R: TCCCAACCAGTGAGACTTAGC | Cloning *rmf* coding region |
| *rmf* RT | F: CAGAAACGAGACCGCCTTGA R: GCGTCCTGTAATGCCAGCTT | qRT-PCR (64°C) |
| *bfr* CDS | F: ATGAAGGGCGATGCGAAAATCATAAG R: TTACTCTTCTTTGATTTGCGCCTG | Cloning *bfr* coding region |
| *bfr* RT | F: TGATTACGTAAGCCGCGATATG R: CAGTCGATATGATGCTCCTCATCTT | qRT-PCR (60°C) |
| CKS_3570 CDS | F: TTTATTCACGATCTGATTAACTGGATTGAC  R: AACACCATACTGACGCTTGAAGC | Cloning CKS_3570 coding region |
| CKS_3570 RT | F: CTCGATCTCGATACGGTTTCTGA R: ATCCGTTGCAGGTGCCATT | qRT-PCR (64°C) |
| *aceB* CDS | F: ATGACAGACTCAGTTATTACCCACGAATTACAC R: TTACGTGCGCTGTCTTTACTTGG | Cloning *aceB* coding region |
| *aceB* RT | F: TGGCTGGCACATTGTCTCATA R: GGATCGGCGCGAAGCT | qRT-PCR (60°C) |
| *yeaG* CDS | F: TACCTTGGCACCATTATGTCG R: ATCGTCGTGTTTCTTCTGCTCATCC | Cloning *yeaG* coding region |
| *yeaG* RT | F: ACCGACCCGAAAGCGAAAT R: CGTCCACGCCCGCATA | qRT-PCR (64°C) |
| CKS_2505 CDS | F: CTACTGGCTTCCGTATTCCATCG  R: ATGAACGGCTTAATGAAACTCGATCG | Cloning CKS_2505 coding region |
| CKS_2505 RT | F: TTTGGGCATCGAGCATCTTC  R: CGCTTTATCACCCGCAGTATTG | qRT-PCR (64°C) |
| *hupA* CDS | F: AAAGCTGACCTGTCTAAAACCCAG R: ACGGCGTCTTTCAGAGCTTTACC | Cloning *hupA* coding region |
| *hupA* RT | F: GCTGAGCGTACCGGTCGTAA R: TGCCGCAGCGATTTTGAT | qRT-PCR (64°C) |
| CKS_4537 CDS | F: CGGAAGTTCTGAATAATGGCTGCG  R: CTGATACAAACCCAAGCCCCACG | Cloning CKS_4537 coding region |
| CKS_4537 RT | F: CAAGAGCCTTTTGGGCATCCT  R: CTCGGTCGCATTCGAAACC | qRT-PCR (60°C) |
| *recF* CDS | F: TTAATCTTTAGGTTGAACCGCTATTTTACCCTG  R: GCTTTAACCCGCCTGCTAATAAAAGATTTTCG | Cloning *recF* coding region |
| *recF* RT | F: AGAGGCAAAGTCATCAATGAGGTAAA  R: GGGCGAGTTCCTGACTAACCA | qRT-PCR (60°C) |
| *atpD* CDS | F: TTAAGAATGGTGATGCTCGTCTGG R: CTTCTTCGATGGCACCAACC | Cloning *atpD* coding region |
| *atpD* RT | F: GGTGCGGGTGTGGGTAAAA R: GCTCAGCCGCAATGTTACG | qRT-PCR (64°C) |
| *gyrB* CDS | F: TTAGATATCGATGTTAGCGGCTTTCAGC  R: ATGTCGAATTCTTATGACTCTTCAAGTATCAAAGTTCTG | Cloning *gyrB* coding region |
| *gyrB* RT | F: GACGTGACCACGCTCAATAATTTC  R: CGGCTCGCACATTCGTACA | qRT-PCR (60°C) |

^a^Primers listed as coding DNA sequence (CDS) were for the cloning of each gene into pGEM-T, and RT was for the qRT-PCR protocol.
