# Supplementary material for: Analysis of the in planta transcriptome expressed by the corn pathogen Pantoea stewartii subsp. stewartii via RNA-Seq
Source: PeerJ. 2017 Apr 27;5:e3237. doi: 10.7717/peerj.3237 (PMC5410145; doi:10.7717/peerj.3237)
Supplement: Table S5 — a A = activated in the in planta culture compared to both the pre-inoculum in vitro liquid culture and the in vitro plate culture (lower in the liquid and plate cultures), R = repressed in the in planta culture compared to both the pre-inoculum in vitro liquid culture and the in vitro plate culture (higher in the liquid and plate cultures). [file peerj-05-3237-s005.docx]

**Table S5**. RNA-Seq data of differentially expressed genes found in the *in planta* culture compared to both the pre-inoculum *in vitro* liquid culture and the *in vitro* plate culture^a^

| **Locus Tag** | **Annotation** | | ***In planta* RPM Fold Regulation vs. Liquid** | ***In planta* RPM Fold Regulation vs. Plate** |
| --- | --- | --- | --- | --- |
| CKS_4725 | hypothetical protein | A | 65.18 | 65.98 |
| CKS_3692 | hypothetical protein | A | 56.09 | 73.74 |
| CKS_3263 | HrpA family pilus protein | A | 52.64 | 58.52 |
| CKS_5750 | acid shock protein | A | 49.49 | 40.09 |
| CKS_2320 | putative alkanal monooxygenase | A | 38.39 | 52.46 |
| CKS_3793 | cytochrome d ubiquinol oxidase subunit I | A | 36.48 | 31.45 |
| CKS_3039 | hypothetical protein | A | 35.53 | 25.68 |
| CKS_3355 | periplasmic-binding component of an ABC superfamily ribose transporter | A | 33.06 | 69.73 |
| CKS_2883 | predicted dethiobiotin synthetase | A | 31.33 | 5.53 |
| CKS_3407 | phosphoenolpyruvate synthase | A | 30.76 | 14.54 |
| CKS_2379 | nudix hydrolase | A | 30.68 | 33.59 |
| CKS_3537 | periplasmic-binding component of an ABC superfamily L-arabinose transporter | A | 29.41 | 46.70 |
| CKS_3794 | cytochrome d terminal oxidase subunit II | A | 28.18 | 24.97 |
| CKS_1610 | ATP-binding component of an ABC superfamily taurine transporter | A | 27.50 | 32.62 |
| CKS_1591 | bacterioferritin iron storage and detoxification protein | A | 27.19 | 14.97 |
| CKS_2202 | hypothetical protein | A | 27.12 | 5.45 |
| CKS_2922 | fasciclin-like repeat-containing secreted/surface protein | A | 26.37 | 8.08 |
| CKS_3795 | membrane-bound protein | A | 25.59 | 23.60 |
| CKS_3356 | hypothetical protein | A | 24.51 | 22.66 |
| CKS_2442 | aldehyde dehydrogenase B | A | 23.70 | 22.99 |
| CKS_4281 | hypothetical protein | A | 23.51 | 12.99 |
| CKS_0668 | predicted quinol oxidase subunit | A | 22.60 | 13.06 |
| CKS_2380 | hypothetical protein | A | 22.17 | 30.37 |
| CKS_3948 | predicted inner membrane protein | A | 21.90 | 8.73 |
| CKS_0801 | ATP-binding component of an ABC superfamily predicted amino-acid transporter | A | 21.75 | 16.83 |
| CKS_0368 | L-lactate dehydrogenase FMN-linked | A | 21.46 | 48.38 |
| CKS_0515 | predicted dioxygenase | A | 20.57 | 19.90 |
| CKS_1609 | membrane component of an ABC superfamily taurine transporter | A | 20.25 | 26.81 |
| CKS_2375 | hypothetical protein | A | 20.00 | 11.85 |
| CKS_3270 | HrpF family protein | A | 19.63 | 20.78 |
| CKS_3570 | AraC family transcriptional regulator | A | 19.33 | 45.70 |
| CKS_3281 | HopAM1-1 family type III effector | A | 19.28 | 8.75 |
| CKS_3513 | oxidoreductase domain protein | A | 18.47 | 9.22 |
| CKS_2985 | hypothetical protein | A | 18.01 | 10.92 |
| CKS_3320 | GGDEF domain protein | A | 17.30 | 28.09 |
| CKS_4575 | secretion system chaperone | A | 16.57 | 14.78 |
| CKS_3796 | YbgE family protein | A | 15.82 | 11.51 |
| CKS_4955 | permease component of an ABC superfamily transporter | A | 15.79 | 13.66 |
| CKS_4830 | amidohydrolase | A | 15.67 | 15.14 |
| CKS_2384 | hypothetical protein | A | 15.52 | 10.62 |
| CKS_3767 | hypothetical protein | A | 15.27 | 10.45 |
| CKS_4657 | malate synthase A | A | 15.20 | 15.65 |
| CKS_3265 | HrpB family protein | A | 14.99 | 11.13 |
| CKS_2186 | membrane component of an ABC superfamily methyl-galactoside transporter | A | 14.99 | 12.73 |
| CKS_0687 | predicted pirin-related protein | A | 14.88 | 14.37 |
| CKS_3512 | oxidoreductase domain protein | A | 14.69 | 9.87 |
| CKS_3503 | methyl-accepting chemotaxis protein | A | 14.60 | 5.21 |
| CKS_4279 | hypothetical protein | A | 14.60 | 13.27 |
| CKS_2376 | hypothetical protein | A | 14.52 | 8.13 |
| CKS_3267 | type III secretion system lipoprotein | A | 14.32 | 10.36 |
| CKS_0560 | hypothetical protein | A | 14.07 | 9.35 |
| CKS_5439 | hypothetical protein | A | 13.72 | 4.76 |
| CKS_3275 | HrpN family hypersensitivity reaction elicitor | A | 13.62 | 21.21 |
| CKS_4573 | hypothetical protein | A | 13.54 | 8.67 |
| CKS_2488 | ATP-binding component of an ABC superfamily sugar transporter | A | 13.29 | 13.75 |
| CKS_3579 | putative MFS superfamily benzoate transporter | A | 13.24 | 9.44 |
| CKS_1611 | periplasmic-binding component of an ABC superfamily taurine transporter | A | 13.12 | 21.18 |
| CKS_4574 | putative type III secretion system effector protein | A | 12.95 | 9.57 |
| CKS_0908 | pirin | A | 12.78 | 7.18 |
| CKS_4719 | conserved inner membrane protein involved in acetate transport | A | 12.70 | 11.47 |
| CKS_2366 | hypothetical protein | A | 12.63 | 11.00 |
| CKS_2378 | hypothetical protein | A | 12.38 | 6.37 |
| CKS_0370 | hypothetical protein | A | 12.16 | 6.90 |
| CKS_0371 | hypothetical protein | A | 12.05 | 6.81 |
| CKS_2371 | hypothetical protein | A | 11.84 | 8.47 |
| CKS_3319 | predicted inner membrane protein | A | 11.65 | 31.76 |
| CKS_2373 | hypothetical protein | A | 11.65 | 9.27 |
| CKS_2372 | putative transcriptional regulator | A | 11.54 | 11.80 |
| CKS_4954 | aryldialkylphosphatase | A | 11.52 | 9.41 |
| CKS_0204 | ammonium transporter | A | 11.47 | 6.42 |
| CKS_2504 | gamma-aminobutyrate:alpha-ketoglutarate aminotransferase | A | 11.42 | 12.38 |
| CKS_3539 | L-arabinose transport system permease protein | A | 11.35 | 10.82 |
| CKS_0380 | hypothetical protein | A | 11.33 | 17.17 |
| CKS_2984 | hypothetical protein | A | 11.32 | 7.38 |
| CKS_4282 | hypothetical protein | A | 11.32 | 10.81 |
| CKS_4280 | hypothetical protein | A | 11.29 | 6.32 |
| CKS_3715 | hypothetical protein | A | 11.24 | 11.14 |
| CKS_4585 | putative type III secretion system apparatus protein | A | 11.21 | 9.04 |
| CKS_3538 | ATP-binding component of an ABC superfamily L-arabinose transporter | A | 11.16 | 7.80 |
| CKS_1361 | PLP-binding diaminopimelate decarboxylase | A | 11.05 | 8.58 |
| CKS_4249 | putative membrane protein | A | 10.97 | 8.46 |
| CKS_0390 | hypothetical protein | A | 10.95 | 10.51 |
| CKS_2383 | hypothetical protein | A | 10.95 | 11.68 |
| CKS_5150 | 3-isopropylmalate isomerase subunit dehydratase component | A | 10.85 | 10.93 |
| CKS_0948 | mannonate hydrolase | A | 10.84 | 26.64 |
| CKS_0963 | membrane component of an ABC superfamily D-ala-D-ala transporter | A | 10.71 | 7.91 |
| CKS_4658 | isocitrate lyase | A | 10.56 | 11.88 |
| CKS_2855 | pyridine nucleotide transhydrogenase beta subunit | A | 10.52 | 9.35 |
| CKS_2854 | pyridine nucleotide transhydrogenase alpha subunit | A | 10.46 | 7.61 |
| CKS_4572 | hypothetical protein | A | 10.45 | 4.29 |
| CKS_4576 | putative type III secretion system effector protein | A | 10.12 | 11.82 |
| CKS_4283 | hypothetical protein | A | 10.09 | 8.08 |
| CKS_2381 | hypothetical protein | A | 10.07 | 9.28 |
| CKS_2679 | mannose-specific enzyme IID component of PTS | A | 9.99 | 5.94 |
| CKS_3535 | L-arabinose isomerase | A | 9.86 | 5.89 |
| CKS_4586 | secretion system apparatus protein | A | 9.82 | 9.23 |
| CKS_4953 | hypothetical protein | A | 9.78 | 11.42 |
| CKS_4019 | alkanesulfonate monooxygenase FMNH(2)-dependent | A | 9.66 | 12.90 |
| CKS_0367 | DNA-binding transcriptional repressor | A | 9.58 | 33.05 |
| CKS_2680 | mannose-specific enzyme IIC component of PTS | A | 9.47 | 11.86 |
| CKS_3254 | HrpO family protein | A | 9.41 | 9.32 |
| CKS_1762 | drug/metabolite transporter (DMT) superfamily permease | A | 9.37 | 6.19 |
| CKS_3623 | hypothetical protein | A | 9.35 | 7.74 |
| CKS_4829 | integral membrane protein | A | 9.31 | 9.19 |
| CKS_5511 | hypothetical protein | A | 9.29 | 29.97 |
| CKS_1541 | IMP dehydrogenase | A | 9.27 | 4.12 |
| CKS_0962 | periplasmic-binding component of an ABC superfamily D-ala-D-a la transporter | A | 9.26 | 10.58 |
| CKS_3354 | short-chain dehydrogenase/reductase | A | 9.22 | 26.87 |
| CKS_4597 | type III secretion system inner membrane protein | A | 9.20 | 7.11 |
| CKS_3619 | hypothetical protein | A | 9.17 | 8.23 |
| CKS_2495 | FAD dependent oxidoreductase | A | 9.13 | 8.64 |
| CKS_4596 | hypothetical protein | A | 9.09 | 7.22 |
| CKS_2489 | permease component of an ABC superfamily ribose/xylose/arabinose/galactoside transporter | A | 9.08 | 7.54 |
| CKS_5454 | putative cell wall-associated hydrolase | A | 9.07 | 9.73 |
| CKS_3982 | predicted transporter | A | 9.06 | 5.77 |
| CKS_2765 | hypothetical protein | A | 8.99 | 11.83 |
| CKS_3268 | HrpD family protein | A | 8.97 | 4.97 |
| CKS_4331 | PTS system cellobiose-specific IIB component | A | 8.90 | 11.55 |
| CKS_2016 | enoyl-CoA hydratase/3-hydroxyacyl-CoA dehydrogenase | A | 8.89 | 14.07 |
| CKS_2806 | putative formate dehydrogenase oxidoreductase protein | A | 8.84 | 27.14 |
| CKS_4653 | periplasmic binding protein | A | 8.83 | 12.34 |
| CKS_4923 | anaerobic ribonucleoside-triphosphate reductase | A | 8.78 | 7.42 |
| CKS_4463 | sn-glycerol-3-phosphate transporter | A | 8.76 | 14.69 |
| CKS_4583 | secretion system apparatus protein | A | 8.69 | 5.88 |
| CKS_0940 | myo-inositol 2-dehydrogenase | A | 8.68 | 11.09 |
| CKS_2222 | predicted peptidase | A | 8.62 | 6.75 |
| CKS_2800 | L-ribulose-5-phosphate 4-epimerase | A | 8.51 | 5.80 |
| CKS_2792 | aconitate hydratase 1 | A | 8.45 | 6.92 |
| CKS_3201 | membrane component of an ABC superfamily polar amino acid transporter | A | 8.37 | 10.61 |
| CKS_2368 | gp55 family protein | A | 8.35 | 5.10 |
| CKS_2385 | hypothetical protein | A | 8.27 | 4.77 |
| CKS_3258 | HrcJ family type III secretion system component protein | A | 8.21 | 8.65 |
| CKS_0452 | putative peptidase | A | 8.19 | 5.95 |
| CKS_4763 | 23-diketo-L-gulonate dehydrogenase NADH-dependent | A | 8.17 | 8.09 |
| CKS_3703 | L-fucose operon activator | A | 8.13 | 6.46 |
| CKS_4588 | type III secretion system apparatus protein | A | 8.13 | 8.87 |
| CKS_3984 | pyruvate formate-lyase | A | 8.11 | 6.68 |
| CKS_2370 | heptosyltransferase | A | 8.09 | 8.94 |
| CKS_4579 | hypothetical protein | A | 8.08 | 11.19 |
| CKS_4767 | 3-keto-L-gulonate 6-phosphate decarboxylase | A | 8.02 | 14.79 |
| CKS_0306 | medium-long-chain fatty acyl-CoA dehydrogenase | A | 8.01 | 42.17 |
| CKS_3858 | molybdopterin biosynthesis protein A | A | 8.01 | 4.92 |
| CKS_0961 | D-ala-D-ala dipeptidase Zn-dependent | A | 7.99 | 9.07 |
| CKS_4272 | putative DNA modification methylase | A | 7.97 | 5.65 |
| CKS_2185 | ATP-binding component of an ABC superfamily galactose/methyl galactoside transporter | A | 7.93 | 28.73 |
| CKS_0468 | hypothetical protein | A | 7.91 | 5.98 |
| CKS_5152 | 2-isopropylmalate synthase | A | 7.89 | 5.83 |
| CKS_0266 | cytosine/purine/uracil/thiamine/allantoin permease family protein | A | 7.83 | 5.12 |
| CKS_0964 | membrane component of an ABC superfamily D-ala-D-ala transporter | A | 7.76 | 9.44 |
| CKS_2106 | hypothetical protein | A | 7.75 | 4.27 |
| CKS_3278 | methyl-accepting chemotaxis sensory transducer | A | 7.67 | 4.77 |
| CKS_3038 | iron-uptake factor | A | 7.66 | 8.57 |
| CKS_3837 | galactose-1-epimerase (mutarotase) | A | 7.65 | 7.05 |
| CKS_3739 | membrane component of an ABC superfamily glutamate and aspartate transporter | A | 7.62 | 6.69 |
| CKS_3569 | RND multidrug efflux membrane fusion protein | A | 7.61 | 10.87 |
| CKS_4584 | putative type III secretion system apparatus protein | A | 7.59 | 6.50 |
| CKS_1750 | N-acetylmuramoyl-L-alanine amidase | A | 7.58 | 10.94 |
| CKS_2563 | putative mannosyl-3-phosphoglycerate phosphatase | A | 7.53 | 5.03 |
| CKS_2490 | DNA-binding transcriptional dual regulator of nitrogen assimilation | A | 7.52 | 8.41 |
| CKS_4021 | NAD(P)H-dependent FMN reductase | A | 7.50 | 4.73 |
| CKS_0288 | putative permease component of an ABC superfamily amino acid transporter | A | 7.50 | 8.29 |
| CKS_1697 | divalent cation transport protein | A | 7.49 | 4.57 |
| CKS_5453 | hypothetical protein | A | 7.48 | 6.25 |
| CKS_0429 | hypothetical protein | A | 7.47 | 5.47 |
| CKS_4333 | beta-glucosidase | A | 7.43 | 8.87 |
| CKS_2635 | hypothetical protein | A | 7.37 | 5.69 |
| CKS_2586 | putative adenine methylase | A | 7.35 | 9.87 |
| CKS_3094 | GAF domain/GGDEF domain/EAL domain protein | A | 7.34 | 4.84 |
| CKS_4761 | periplasmic substrate-binding component of an ABC superfamily ribose transporter | A | 7.30 | 8.66 |
| CKS_3738 | permease component of an ABC superfamily glutamate/aspartate transporter | A | 7.29 | 5.51 |
| CKS_3534 | membrane-fusion protein | A | 7.27 | 5.42 |
| CKS_3859 | molybdopterin biosynthesis protein B | A | 7.26 | 6.65 |
| CKS_0290 | putative ATP-binding component of an ABC superfamily amino acid transporter | A | 7.26 | 5.83 |
| CKS_4952 | acetylornithine deacetylase | A | 7.23 | 7.31 |
| CKS_4273 | hypothetical protein | A | 7.21 | 4.20 |
| CKS_2803 | hypothetical protein | A | 7.20 | 7.16 |
| CKS_4654 | hypothetical protein | A | 7.19 | 9.24 |
| CKS_3146 | dihydrodipicolinate synthase | A | 7.18 | 5.93 |
| CKS_1233 | protein disaggregation chaperone | A | 7.17 | 9.25 |
| CKS_2441 | methyltransferase | A | 7.15 | 4.22 |
| CKS_2493 | aldehyde dehydrogenase | A | 7.14 | 7.50 |
| CKS_2924 | metal-activated pyridoxal enzyme | A | 7.02 | 5.75 |
| CKS_2377 | hypothetical protein | A | 6.96 | 4.46 |
| CKS_2492 | succinate-semialdehyde dehydrogenase I NADP-dependent | A | 6.92 | 6.77 |
| CKS_2487 | putative periplasmic binding protein | A | 6.80 | 10.60 |
| CKS_2525 | permease component of an ABC superfamily ribose transporter | A | 6.77 | 6.88 |
| CKS_4056 | WrbA family flavoprotein | A | 6.71 | 4.85 |
| CKS_0338 | glycerol dehydrogenase NAD | A | 6.66 | 25.84 |
| CKS_0912 | hypothetical protein | A | 6.60 | 4.69 |
| CKS_5151 | 3-isopropylmalate dehydrogenase | A | 6.59 | 5.06 |
| CKS_2642 | hypothetical protein | A | 6.57 | 6.54 |
| CKS_3276 | putative avirulence protein | A | 6.56 | 4.39 |
| CKS_2983 | phytochelatin synthase | A | 6.41 | 4.51 |
| CKS_2062 | permease component of an ABC superfamily spermidine/putrescine transporter | A | 6.36 | 5.47 |
| CKS_4020 | periplasmic-binding component of an ABC superfamily alkanesulfonate transporter | A | 6.35 | 6.26 |
| CKS_4958 | putative binding periplasmic protein of ABC transporter | A | 6.34 | 4.28 |
| CKS_2386 | hypothetical protein | A | 6.34 | 6.16 |
| CKS_3621 | aldo-keto reductase | A | 6.32 | 29.63 |
| CKS_0379 | hypothetical protein | A | 6.30 | 24.08 |
| CKS_0657 | altronate hydrolase | A | 6.21 | 6.80 |
| CKS_4593 | hypothetical protein | A | 6.20 | 6.07 |
| CKS_1037 | predicted transporter | A | 6.19 | 9.17 |
| CKS_3620 | hypothetical protein | A | 6.17 | 4.95 |
| CKS_2012 | long-chain fatty acid outer membrane transporter | A | 6.16 | 5.36 |
| CKS_4762 | gluconolactonase | A | 6.13 | 6.54 |
| CKS_3702 | periplasmic substrate binding component of an ABC superfamily sugar transporter | A | 6.12 | 4.33 |
| CKS_4340 | heat shock chaperone | A | 6.04 | 5.17 |
| CKS_0291 | serine--pyruvate aminotransferase / L-alanine:glyoxylate aminotransferase | A | 6.03 | 7.16 |
| CKS_5149 | 3-isopropylmalate isomerase subunit | A | 6.02 | 4.07 |
| CKS_3271 | type III secretion system outermembrane pore forming protein | A | 5.99 | 5.29 |
| CKS_3206 | putative amidohydrolase | A | 5.97 | 8.15 |
| CKS_3622 | pyruvate/alpha-keto-acid decarboxylase | A | 5.95 | 6.99 |
| CKS_1696 | magnesium transporter | A | 5.91 | 4.93 |
| CKS_4764 | hypothetical protein | A | 5.90 | 5.77 |
| CKS_4760 | permease component of an ABC superfamily ribose transporter | A | 5.89 | 6.99 |
| CKS_5026 | hypothetical protein | A | 5.89 | 5.91 |
| CKS_2921 | putative RNA polymerase sigma factor | A | 5.88 | 5.87 |
| CKS_3540 | DNA-binding transcriptional dual regulator | A | 5.88 | 13.66 |
| CKS_3983 | pyruvate formate lyase activating enzyme 1 | A | 5.86 | 4.85 |
| CKS_1698 | hypothetical protein | A | 5.86 | 4.62 |
| CKS_4484 | 3-ketoacyl-CoA thiolase (thiolase I) | A | 5.85 | 4.91 |
| CKS_3202 | ABC superfamily transporter | A | 5.84 | 4.21 |
| CKS_3860 | molybdopterin biosynthesis protein C | A | 5.84 | 8.33 |
| CKS_0941 | inosose dehydratase | A | 5.83 | 4.56 |
| CKS_4718 | acetate permease | A | 5.78 | 5.03 |
| CKS_0965 | ATP-binding component of an ABC superfamily D-ala-D-ala transporter | A | 5.78 | 6.75 |
| CKS_0922 | periplasmic-binding component of an ABC superfamily leucine transporter | A | 5.76 | 6.69 |
| CKS_4759 | ATP-binding component of an ABC superfamily ribose transporter | A | 5.76 | 12.00 |
| CKS_4571 | virulence protein | A | 5.73 | 4.72 |
| CKS_2641 | phage baseplate assembly protein V | A | 5.67 | 7.15 |
| CKS_4577 | putative type III secretion system effector protein | A | 5.63 | 8.66 |
| CKS_3241 | amidinotransferase family protein | A | 5.60 | 4.40 |
| CKS_2588 | hypothetical protein | A | 5.59 | 5.85 |
| CKS_2640 | hypothetical protein | A | 5.58 | 5.32 |
| CKS_3150 | predicted mannonate dehydrogenase | A | 5.57 | 4.32 |
| CKS_0791 | predicted reductase | A | 5.54 | 5.95 |
| CKS_0376 | putative phage transposase | A | 5.45 | 16.09 |
| CKS_2496 | extracellular solute-binding protein family 5 | A | 5.39 | 5.57 |
| CKS_2880 | predicted transporter | A | 5.36 | 4.91 |
| CKS_4922 | anaerobic ribonucleotide reductase activating protein | A | 5.31 | 5.48 |
| CKS_4247 | putative alpha/beta hydrolase | A | 5.30 | 8.92 |
| CKS_2587 | hypothetical protein | A | 5.28 | 6.21 |
| CKS_4389 | membrane component of an ABC superfamily dipeptide transporter | A | 5.25 | 6.16 |
| CKS_2387 | hypothetical protein | A | 5.24 | 5.64 |
| CKS_5834 | hypothetical protein | A | 5.22 | 5.03 |
| CKS_0375 | putative DNA-binding protein | A | 5.21 | 23.08 |
| CKS_4594 | hypothetical protein | A | 5.17 | 5.93 |
| CKS_4485 | fatty acid oxidation complex subunit alpha | A | 5.17 | 37.14 |
| CKS_1045 | acetolactate synthase large subunit | A | 5.14 | 6.98 |
| CKS_3255 | type III secretion system cytoplasmic ATP synthase | A | 5.03 | 5.20 |
| CKS_3536 | L-ribulokinase | A | 5.02 | 4.63 |
| CKS_4523 | type III secretion system peptide export protein | A | 4.98 | 4.14 |
| CKS_1538 | hypothetical protein | A | 4.97 | 4.78 |
| CKS_2369 | hypothetical protein | A | 4.96 | 4.02 |
| CKS_5446 | hypothetical protein | A | 4.94 | 4.85 |
| CKS_4800 | predicted dehydrogenase | A | 4.94 | 7.68 |
| CKS_3243 | hypothetical protein | A | 4.94 | 6.13 |
| CKS_4162 | hypothetical protein | A | 4.93 | 4.64 |
| CKS_3021 | putative NADH:flavin oxidoreductase | A | 4.92 | 15.50 |
| CKS_4712 | hypothetical protein | A | 4.92 | 4.82 |
| CKS_2550 | DgsA-binding anti-repressor | A | 4.91 | 5.49 |
| CKS_2920 | hypothetical protein | A | 4.90 | 5.85 |
| CKS_4956 | ABC transporter | A | 4.89 | 14.79 |
| CKS_2804 | putative carbon starvation protein A | A | 4.84 | 14.30 |
| CKS_3826 | PhoH family ATPase | A | 4.83 | 7.51 |
| CKS_0943 | 5-deoxy-glucuronate isomerase | A | 4.80 | 4.71 |
| CKS_2494 | type II haloacid dehalogenase | A | 4.80 | 4.81 |
| CKS_0921 | membrane component of an ABC superfamily leucine/isoleucine/valine transporter | A | 4.79 | 5.36 |
| CKS_4332 | PTS system cellobiose-specific IIC component | A | 4.78 | 6.35 |
| CKS_0377 | phage transposase | A | 4.76 | 11.68 |
| CKS_3257 | type III secretion system inner membrane channel protein | A | 4.76 | 4.25 |
| CKS_0640 | zinc-containing alcohol dehydrogenase/quinone oxidoreductase | A | 4.76 | 4.68 |
| CKS_2361 | DNA adenine methylase | A | 4.74 | 4.14 |
| CKS_4591 | secretion system apparatus protein | A | 4.73 | 5.26 |
| CKS_5025 | phage-related protein | A | 4.68 | 4.74 |
| CKS_5455 | putative exported protein | A | 4.68 | 18.99 |
| CKS_3273 | HrpT family protein | A | 4.64 | 4.48 |
| CKS_4592 | putative type III secretion system ATP synthase | A | 4.60 | 5.53 |
| CKS_2015 | acetyl-CoA acetyltransferase | A | 4.59 | 35.62 |
| CKS_2644 | hypothetical protein | A | 4.57 | 5.21 |
| CKS_2524 | ATP-binding component of an ABC superfamily ribose transporter | A | 4.55 | 4.40 |
| CKS_1044 | acetolactate synthase small subunit | A | 4.54 | 9.52 |
| CKS_5349 | putative exported protein | A | 4.53 | 17.83 |
| CKS_0960 | putative RpiR family transcriptional regulator | A | 4.52 | 14.48 |
| CKS_5754 | hypothetical protein | A | 4.51 | 4.96 |
| CKS_2589 | phage-related protein | A | 4.50 | 4.82 |
| CKS_2306 | uncharacterized DUF1537 family protein | A | 4.50 | 7.26 |
| CKS_0658 | altronate oxidoreductase NAD-dependent | A | 4.49 | 6.43 |
| CKS_3968 | cold shock protein | A | 4.48 | 16.43 |
| CKS_5389 | putative exported protein | A | 4.47 | 16.57 |
| CKS_5413 | hypothetical protein | A | 4.45 | 9.95 |
| CKS_1289 | endoribonuclease L-PSP | A | 4.45 | 9.98 |
| CKS_3552 | mannose-6-phosphate isomerase | A | 4.44 | 5.22 |
| CKS_4462 | HD superfamily hydrolase | A | 4.42 | 4.53 |
| CKS_5393 | putative exported protein | A | 4.42 | 17.45 |
| CKS_3844 | membrane component of an ABC superfamily molybdate transporter | A | 4.42 | 8.21 |
| CKS_2307 | predicted class II aldolase | A | 4.38 | 4.22 |
| CKS_2947 | maltose regulon periplasmic protein | A | 4.33 | 4.71 |
| CKS_2637 | hypothetical protein | A | 4.33 | 5.41 |
| CKS_3147 | alcohol dehydrogenase | A | 4.30 | 5.33 |
| CKS_3143 | hypothetical protein | A | 4.26 | 4.01 |
| CKS_5356 | putative outer membrane adhesion protein | A | 4.23 | 5.06 |
| CKS_3210 | predicted oxidoreductase flavin:NADH component | A | 4.18 | 4.03 |
| CKS_2590 | hypothetical protein | A | 4.17 | 5.92 |
| CKS_1444 | GroES family alcohol dehydrogenase | A | 4.16 | 4.07 |
| CKS_2706 | PLP-binding alanine racemase 2 | A | 4.08 | 4.43 |
| CKS_2629 | phage tail sheath protein FI | A | 4.08 | 6.56 |
| CKS_0272 | phytanoyl-CoA dioxygenase family protein | A | 4.01 | 4.01 |
| CKS_4368 | superoxide dismutase Mn | R | 4.05 | 4.10 |
| CKS_0117 | peptidyl-prolyl cis-trans isomerase B (rotamase B) | R | 4.08 | 4.56 |
| CKS_4123 | putative bacteriophage protein | R | 4.09 | 5.31 |
| CKS_4125 | putative bacteriophage protein | R | 4.42 | 5.26 |
| CKS_4121 | putative bacteriophage protein | R | 4.51 | 7.29 |
| CKS_0004 | HU DNA-binding transcriptional regulator alpha subunit | R | 4.58 | 4.71 |
| CKS_4122 | hypothetical protein | R | 4.69 | 6.27 |
| CKS_4134 | putative bacteriophage protein | R | 4.76 | 6.95 |
| CKS_5198 | dihydrolipoamide dehydrogenase | R | 4.82 | 5.03 |
| CKS_0744 | 30S ribosomal subunit protein S6 | R | 4.84 | 4.60 |
| CKS_4717 | putative exported protein | R | 4.87 | 6.62 |
| CKS_4536 | type III secretion system effector protein | R | 4.99 | 11.16 |
| CKS_4535 | type III secretion system regulatory protein | R | 5.36 | 8.39 |
| CKS_0742 | 30S ribosomal subunit protein S18 | R | 5.52 | 4.91 |
| CKS_4124 | hypothetical protein | R | 5.71 | 8.12 |
| CKS_1527 | nucleoside diphosphate kinase | R | 5.76 | 4.06 |
| CKS_4120 | putative bacteriophage protein | R | 5.78 | 8.63 |
| CKS_4115 | putative bacteriophage protein | R | 6.00 | 6.74 |
| CKS_4116 | putative bacteriophage protein | R | 6.00 | 7.72 |
| CKS_0743 | primosomal replication protein N | R | 6.09 | 5.48 |
| CKS_4117 | putative bacteriophage protein | R | 6.11 | 7.45 |
| CKS_4137 | hypothetical protein | R | 6.20 | 4.87 |
| CKS_4135 | putative bacteriophage protein | R | 6.25 | 7.93 |
| CKS_4109 | putative bacteriophage protein | R | 6.28 | 5.60 |
| CKS_0741 | 50S ribosomal subunit protein L9 | R | 6.33 | 5.37 |
| CKS_1116 | ADP-heptose--lipooligosaccharide heptosyltransferase II | R | 6.57 | 4.88 |
| CKS_4113 | hypothetical protein | R | 6.72 | 8.85 |
| CKS_4114 | putative bacteriophage protein | R | 7.32 | 7.69 |
| CKS_4119 | putative bacteriophage protein | R | 7.55 | 10.89 |
| CKS_4531 | type III secretion system apparatus protein | R | 7.65 | 14.13 |
| CKS_4091 | hypothetical protein | R | 7.82 | 4.06 |
| CKS_4118 | putative bacteriophage protein | R | 7.99 | 10.71 |
| CKS_4092 | hypothetical protein | R | 8.20 | 5.84 |
| CKS_4136 | putative bacteriophage tail protein | R | 8.32 | 6.45 |
| CKS_1241 | 3-deoxy-D-arabino-heptulosonate-7-phosphate synthase tyrosine-repressible | R | 8.70 | 6.41 |
| CKS_4538 | type III secretion system apparatus protein | R | 8.80 | 15.91 |
| CKS_1225 | hypothetical protein | R | 8.91 | 23.57 |
| CKS_4095 | hypothetical protein | R | 9.89 | 4.03 |
| CKS_4089 | hypothetical protein | R | 13.42 | 4.95 |
| CKS_4090 | hypothetical protein | R | 13.63 | 6.30 |
| CKS_4088 | hypothetical protein | R | 16.02 | 4.33 |
| CKS_4082 | hypothetical protein | R | 16.02 | 4.10 |
| CKS_4093 | CP4-57 family phage integrase | R | 16.35 | 5.42 |
| CKS_4081 | hypothetical protein | R | 16.79 | 4.61 |
| CKS_4084 | phage lysozyme | R | 17.69 | 5.00 |
| CKS_4537 | type III secretion system effector protein | R | 18.27 | 36.62 |
| CKS_4085 | hypothetical protein | R | 18.62 | 5.25 |
| CKS_4083 | hypothetical protein | R | 22.82 | 6.36 |
| CKS_4087 | hypothetical protein | R | 25.57 | 6.99 |

^a^A = activated in the *in planta* culture compared to both the pre-inoculum *in vitro* liquid culture and the *in vitro* plate culture (lower in the liquid and plate cultures), R = repressed in the *in planta* culture compared to both the pre-inoculum *in vitro* liquid culture and the *in vitro* plate culture (higher in the liquid and plate cultures)
