# Supplementary material for: Analysis of the in planta transcriptome expressed by the corn pathogen Pantoea stewartii subsp. stewartii via RNA-Seq
Source: PeerJ. 2017 Apr 27;5:e3237. doi: 10.7717/peerj.3237 (PMC5410145; doi:10.7717/peerj.3237)
Supplement: Table S6 — a R = repressed in the in planta culture compared to either the pre-inoculum in vitro liquid culture or the in vitro plate culture (higher in the liquid or plate cultures). [file peerj-05-3237-s006.docx]

**Table S6.** Additional genes with greater than four-fold regulation as calculated through the DESeq analysis^a^

| **Locus Tag** | **Annotation** | | **RPM Fold Regulation** | **DESeq Fold Regulation** | **DESeq padj** |
| --- | --- | --- | --- | --- | --- |
| ***In planta* vs. Pre-inoculum *in vitro* Liquid Culture** | | | | | |
| CKS_1022 | predicted DNA-binding transcriptional regulator | R | 3.91 | 5.44 | 5.70E-31 |
| CKS_1399 | component of the MscS mechanosensitive channel | R | 3.92 | 5.43 | 4.16E-30 |
| CKS_1838 | predicted metal-binding enzyme | R | 3.83 | 5.35 | 3.30E-28 |
| CKS_2276 | gluconate-6-phosphate dehydrogenase decarboxylating | R | 3.83 | 5.33 | 3.76E-28 |
| CKS_0841 | hypothetical protein | R | 3.80 | 5.31 | 3.09E-28 |
| CKS_5399 | hypothetical protein | R | 3.75 | 5.24 | 1.09E-26 |
| CKS_1952 | hypothetical protein | R | 3.63 | 5.08 | 1.56E-26 |
| CKS_4126 | putative bacteriophage protein | R | 3.64 | 5.06 | 1.32E-17 |
| CKS_0644 | diaminobutyrate-pyruvate transaminase/L-24-diaminobutyrate decarboxylase | R | 3.85 | 5.04 | 2.46E-09 |
| CKS_1580 | 50S ribosomal subunit protein L29 | R | 3.62 | 5.04 | 6.05E-16 |
| CKS_3122 | DNA-binding protein H-NS | R | 3.59 | 5.01 | 2.73E-22 |
| CKS_4472 | uridine phosphorylase | R | 3.53 | 4.92 | 4.60E-30 |
| CKS_4775 | IucB family aerobactin siderophore biosynthesis protein | R | 3.66 | 4.89 | 6.42E-12 |
| CKS_0018 | 50S ribosomal subunit protein L1 | R | 3.52 | 4.89 | 1.02E-15 |
| CKS_1166 | hypothetical protein | R | 3.48 | 4.84 | 1.32E-23 |
| CKS_2826 | lipid hydroperoxide peroxidase | R | 3.46 | 4.82 | 2.28E-29 |
| CKS_0093 | hypothetical protein | R | 3.62 | 4.79 | 8.12E-07 |
| CKS_4927 | DNA-binding transcriptional repressor | R | 3.41 | 4.78 | 2.93E-28 |
| CKS_5361 | hypothetical protein | R | 3.42 | 4.74 | 9.07E-15 |
| CKS_1145 | 50S ribosomal subunit protein L33 | R | 3.39 | 4.73 | 3.45E-19 |
| CKS_5267 | outer membrane protein | R | 3.39 | 4.72 | 1.43E-26 |
| CKS_4782 | LysR family transcriptional regulator | R | 3.33 | 4.65 | 9.41E-29 |
| CKS_4076 | hypothetical protein | R | 3.53 | 4.58 | 1.54E-05 |
| CKS_5596 | putative phage portal protein | R | 3.75 | 4.58 | 5.40E-04 |
| CKS_5594 | putative phage terminase large subunit | R | 3.87 | 4.58 | 1.18E-03 |
| CKS_3802 | peptidoglycan-associated outer membrane lipoprotein | R | 3.28 | 4.55 | 1.10E-19 |
| CKS_3391 | 50S ribosomal subunit protein L20 | R | 3.22 | 4.52 | 3.89E-28 |
| CKS_1267 | membrane-bound lytic murein transglycosylase B | R | 3.20 | 4.50 | 6.73E-26 |
| CKS_4530 | type III secretion system cytoplasmic ATP synthase | R | 3.18 | 4.46 | 4.68E-28 |
| CKS_4835 | putative LysR-type transcriptional regulator | R | 3.18 | 4.45 | 1.07E-20 |
| CKS_0022 | translation elongation factor Tu | R | 3.21 | 4.44 | 3.32E-11 |
| CKS_1581 | 50S ribosomal subunit protein L16 | R | 3.18 | 4.43 | 2.06E-13 |
| CKS_4127 | putative bacteriophage protein | R | 3.20 | 4.43 | 6.73E-12 |
| CKS_1087 | putative cytoplasmic protein | R | 3.13 | 4.38 | 6.76E-29 |
| CKS_5600 | putative phage capsid protein | R | 3.60 | 4.35 | 1.09E-03 |
| CKS_0852 | predicted transporter | R | 3.09 | 4.32 | 9.17E-26 |
| CKS_5582 | hypothetical protein | R | 3.74 | 4.31 | 2.85E-03 |
| CKS_4862 | 30S ribosomal subunit protein S9 | R | 3.02 | 4.24 | 3.67E-21 |
| CKS_0181 | DNA-binding protein | R | 3.02 | 4.23 | 2.30E-18 |
| CKS_5792 | predicted transcriptional regulator | R | 3.02 | 4.22 | 7.48E-19 |
| CKS_0220 | peptidyl-prolyl cis/trans isomerase (trigger factor) | R | 3.03 | 4.22 | 1.41E-12 |
| CKS_4238 | predicted metallodependent hydrolase | R | 3.00 | 4.21 | 1.08E-22 |
| CKS_4269 | NADP-specific isocitrate dehydrogenase | R | 3.00 | 4.18 | 5.60E-15 |
| CKS_5589 | hypothetical protein | R | 3.63 | 4.18 | 4.66E-03 |
| CKS_4108 | hypothetical protein | R | 3.05 | 4.17 | 1.60E-07 |
| CKS_1582 | 30S ribosomal subunit protein S3 | R | 3.00 | 4.17 | 4.45E-11 |
| CKS_0936 | DcrB family protein | R | 2.98 | 4.17 | 7.15E-17 |
| CKS_4226 | 50S ribosomal subunit protein L32 | R | 2.96 | 4.17 | 4.75E-27 |
| CKS_1096 | hypothetical protein | R | 3.00 | 4.16 | 3.34E-18 |
| CKS_4132 | phage-related protein | R | 2.98 | 4.16 | 8.55E-16 |
| CKS_1579 | 30S ribosomal subunit protein S17 | R | 2.96 | 4.14 | 5.24E-13 |
| CKS_0625 | hypothetical protein | R | 2.95 | 4.09 | 4.00E-10 |
| CKS_4774 | IucC family aerobactin siderophore biosynthesis protein | R | 3.11 | 4.09 | 1.23E-07 |
| CKS_2189 | cytidine/deoxycytidine deaminase | R | 2.96 | 4.08 | 2.05E-15 |
| CKS_2277 | O-antigen lipopolysaccharide chain length regulator | R | 2.91 | 4.07 | 5.09E-19 |
| CKS_5258 | 30S ribosomal subunit protein S2 | R | 2.90 | 4.07 | 1.83E-20 |
| CKS_3134 | hypothetical protein | R | 2.90 | 4.07 | 1.13E-16 |
| CKS_1453 | alcohol dehydrogenase | R | 2.96 | 4.06 | 2.92E-08 |
| CKS_4721 | glutamate/aspartate:proton symporter | R | 2.89 | 4.04 | 2.40E-19 |
| CKS_2811 | enoyl-[acyl-carrier-protein] reductase NADH-dependent | R | 2.87 | 4.03 | 2.58E-22 |
| CKS_3164 | hypothetical protein | R | 2.89 | 4.02 | 8.07E-18 |
| CKS_0733 | FKBP-type peptidyl-prolyl cis-trans isomerase (rotamase) | R | 2.86 | 4.02 | 2.03E-22 |
| CKS_3390 | 50S ribosomal subunit protein L35 | R | 2.86 | 4.02 | 6.40E-22 |
| CKS_4139 | putative DNA damage-inducible protein | R | 3.00 | 4.02 | 1.10E-05 |
| ***In planta* vs. *In vitro* Plate Culture** | | | | | |
| CKS_5267 | outer membrane protein | R | 3.88 | 4.95 | 8.37E-19 |
| CKS_0667 | inner membrane protein | R | 3.84 | 4.86 | 9.03E-21 |
| CKS_2811 | enoyl-[acyl-carrier-protein] reductase NADH-dependent | R | 3.78 | 4.80 | 8.65E-20 |
| CKS_3056 | ThiG family thiazole biosynthesis protein | R | 3.78 | 4.76 | 4.02E-22 |
| CKS_0714 | uncharacterized DUF615 family protein | R | 3.73 | 4.75 | 3.24E-18 |
| CKS_3134 | hypothetical protein | R | 3.74 | 4.75 | 2.59E-16 |
| CKS_2419 | hypothetical protein | R | 3.73 | 4.74 | 5.20E-19 |
| CKS_4984 | hypothetical protein | R | 3.73 | 4.73 | 2.50E-14 |
| CKS_2158 | putative membrane protein | R | 3.74 | 4.73 | 7.99E-12 |
| CKS_2884 | putative ATP/GTP-binding component of an ABC superfamily transporter | R | 3.87 | 4.73 | 1.34E-12 |
| CKS_2276 | gluconate-6-phosphate dehydrogenase decarboxylating | R | 3.74 | 4.72 | 3.14E-14 |
| CKS_0017 | 50S ribosomal subunit protein L10 | R | 3.63 | 4.61 | 2.21E-13 |
| CKS_2217 | hydoxyethylthiazole kinase | R | 3.61 | 4.59 | 1.18E-15 |
| CKS_3089 | 23S rRNA pseudouridine synthase | R | 3.61 | 4.58 | 1.49E-13 |
| CKS_1838 | predicted metal-binding enzyme | R | 3.58 | 4.56 | 2.16E-22 |
| CKS_5259 | protein chain elongation factor EF-Ts | R | 3.59 | 4.56 | 2.47E-15 |
| CKS_5399 | hypothetical protein | R | 3.58 | 4.54 | 2.99E-13 |
| CKS_0841 | hypothetical protein | R | 3.57 | 4.53 | 3.04E-13 |
| CKS_0132 | hypothetical protein | R | 3.63 | 4.52 | 1.05E-10 |
| CKS_1466 | uncharacterized DUF2002 family protein | R | 3.66 | 4.50 | 6.80E-10 |
| CKS_4138 | hypothetical protein | R | 3.51 | 4.45 | 2.21E-14 |
| CKS_2428 | hypothetical protein | R | 3.53 | 4.43 | 2.84E-18 |
| CKS_0800 | global DNA-binding transcriptional dual regulator | R | 3.45 | 4.39 | 8.76E-12 |
| CKS_2826 | lipid hydroperoxide peroxidase | R | 3.46 | 4.38 | 1.27E-21 |
| CKS_4269 | NADP-specific isocitrate dehydrogenase | R | 3.44 | 4.37 | 6.48E-15 |
| CKS_1087 | putative cytoplasmic protein | R | 3.43 | 4.36 | 8.99E-14 |
| CKS_0740 | hypothetical protein | R | 3.44 | 4.31 | 1.10E-09 |
| CKS_1267 | membrane-bound lytic murein transglycosylase B | R | 3.37 | 4.30 | 7.90E-21 |
| CKS_1145 | 50S ribosomal subunit protein L33 | R | 3.34 | 4.23 | 1.32E-09 |
| CKS_0220 | peptidyl-prolyl cis/trans isomerase (trigger factor) | R | 3.33 | 4.23 | 2.47E-15 |
| CKS_0181 | DNA-binding protein | R | 3.31 | 4.22 | 1.04E-18 |
| CKS_2726 | predicted oxidoreductase | R | 3.29 | 4.19 | 6.27E-16 |
| CKS_1952 | hypothetical protein | R | 3.29 | 4.19 | 1.35E-15 |
| CKS_1096 | hypothetical protein | R | 3.29 | 4.16 | 2.24E-13 |
| CKS_5255 | 2345-tetrahydropyridine-2-carboxylate N-succinyltransferase | R | 3.25 | 4.15 | 5.55E-18 |
| CKS_4099 | putative phage primase | R | 3.28 | 4.14 | 4.66E-12 |
| CKS_1904 | hypothetical protein | R | 3.36 | 4.10 | 1.88E-05 |
| CKS_2187 | uncharacterized DUF218 family protein | R | 3.20 | 4.09 | 2.85E-18 |
| CKS_5195 | DNA-binding transcriptional dual regulator | R | 3.18 | 4.06 | 2.36E-16 |
| CKS_0666 | conserved inner membrane protein | R | 3.15 | 4.01 | 9.59E-17 |
| CKS_5274 | acetyl-CoA carboxylase carboxytransferase alpha subunit | R | 3.13 | 4.00 | 8.79E-14 |

^a^R = repressed in the *in planta* culture compared to either the pre-inoculum *in vitro* liquid culture or the *in vitro* plate culture (higher in the liquid or plate cultures)
