# Supplementary material for: Analysis of the in planta transcriptome expressed by the corn pathogen Pantoea stewartii subsp. stewartii via RNA-Seq
Source: PeerJ. 2017 Apr 27;5:e3237. doi: 10.7717/peerj.3237 (PMC5410145; doi:10.7717/peerj.3237)
Supplement: Table S7 — Genes upregulated (activated) in planta, with the exception of those designated * which were downregulated (repressed) in planta. [file peerj-05-3237-s007.docx]

**Table S7.** Results for qRT-PCR validation for the *in planta* culture and the pre-inoculum *in vitro* liquid culture comparison^a^.

| **Locus Tag** | **Gene** | **Fold Regulation (*recF* reference)** | **Fold Regulation (*atpD* reference)** | **Fold Regulation (*gyrB* reference)** | **RNA-Seq RPM Fold Regulation** |
| --- | --- | --- | --- | --- | --- |
| CKS_3263 |  | 139.65 | 38.51 | 244.12 | 52.64 |
| CKS_3793 |  | 15.47 | 4.27 | 27.04 | 36.48 |
| CKS_4032 | *rmf* | 34.02 | 9.38 | 59.47 | 28.23 |
| CKS_1591 | *bfr* | 31.85 | 8.78 | 55.68 | 27.19 |
| CKS_3570 |  | 39.12 | 10.79 | 68.39 | 19.33 |
| CKS_4657 | *aceB* | 45.69 | 12.60 | 79.88 | 15.20 |
| CKS_2714 | *yeaG* | 44.55 | 12.28 | 77.88 | 8.22 |
| CKS_2505 |  | 7.92 | 2.18 | 13.85 | 4.20 |
| CKS_0004* | *hupA* | 2.22 | 8.33 | 1.27 | 4.58 |
| CKS_4537* |  | 3.70 | 14.29 | 2.13 | 18.27 |

^a^Genes upregulated (activated) *in planta*, with the exception of those designated * which were downregulated (repressed) *in planta.*
