# Supplementary material for: Analysis of the in planta transcriptome expressed by the corn pathogen Pantoea stewartii subsp. stewartii via RNA-Seq
Source: PeerJ. 2017 Apr 27;5:e3237. doi: 10.7717/peerj.3237 (PMC5410145; doi:10.7717/peerj.3237)
Supplement: Table S8 — Genes upregulated (activated) in planta, with the exception of those designated * which were downregulated (repressed) in planta. [file peerj-05-3237-s008.docx]

**Table S8.** Results for qRT-PCR validation for the *in planta* culture and the *in vitro* plate culture comparison^a^.

| **Locus Tag** | **Gene** | **Fold Regulation (*recF* reference)** | **Fold Regulation (*atpD* reference)** | **Fold Regulation (*gyrB* reference)** | **RNA-Seq RPM Fold Regulation** |
| --- | --- | --- | --- | --- | --- |
| CKS_3263 |  | 188.99 | 159.30 | 200.06 | 58.52 |
| CKS_3793 |  | 47.56 | 40.09 | 50.34 | 31.45 |
| CKS_4032 | *rmf* | 4.38 | 3.69 | 4.63 | 3.70 |
| CKS_1591 | *bfr* | 26.75 | 22.54 | 28.31 | 14.97 |
| CKS_3570 |  | 24.22 | 20.42 | 25.64 | 45.70 |
| CKS_4657 | *aceB* | 35.46 | 29.89 | 37.54 | 15.65 |
| CKS_2714 | *yeaG* | 6.97 | 5.88 | 7.38 | 3.16 |
| CKS_2505 |  | 5.46 | 4.60 | 5.78 | 2.13 |
| CKS_0004* | *hupA* | 3.85 | 4.55 | 3.57 | 4.71 |
| CKS_4537* |  | 3.45 | 4.17 | 3.33 | 36.62 |

^a^Genes upregulated (activated) *in planta*, with the exception of those designated * which were downregulated (repressed) *in planta.*
