# Supplementary material for: Analysis of the in planta transcriptome expressed by the corn pathogen Pantoea stewartii subsp. stewartii via RNA-Seq
Source: PeerJ. 2017 Apr 27;5:e3237. doi: 10.7717/peerj.3237 (PMC5410145; doi:10.7717/peerj.3237)
Supplement: Table S9 [file peerj-05-3237-s009.docx]

**Table S9.** GO gene groups from four-fold regulated genes in the *in planta* culture compared to the pre-inoculum *in vitro* liquid culture.

| **GO.ID** | **Term** | **Annotated** | **Significant** | **Expected** | **weight01Fisher** |
| --- | --- | --- | --- | --- | --- |
| **Upregulated *in planta*** | | | | | |
| GO:0055114 | oxidation-reduction process | 164 | 31 | 12.6 | 1.10E-06 |
| GO:0009102 | biotin biosynthetic process | 7 | 6 | 0.54 | 1.30E-06 |
| GO:0009098 | leucine biosynthetic process | 4 | 4 | 0.31 | 3.40E-05 |
| GO:0006810 | transport | 510 | 61 | 39.2 | 0.00035 |
| GO:0009405 | pathogenesis | 17 | 5 | 1.31 | 0.0073 |
| GO:0009306 | protein secretion | 41 | 8 | 3.15 | 0.011 |
| **Downregulated *in planta*** | | | | | |
| GO:0006323 | DNA packaging | 8 | 2 | 0.09 | 0.0030 |
| GO:0006412 | translation | 108 | 6 | 1.17 | 0.0037 |
| GO:0006571 | tyrosine biosynthetic process | 1 | 1 | 0.01 | 0.011 |
| GO:0006228 | UTP biosynthetic process | 1 | 1 | 0.01 | 0.011 |
| GO:0006241 | CTP biosynthetic process | 1 | 1 | 0.01 | 0.011 |
| GO:0015758 | glucose transport | 1 | 1 | 0.01 | 0.011 |
| GO:0006183 | GTP biosynthetic process | 1 | 1 | 0.01 | 0.011 |
| GO:0009405 | pathogenesis | 17 | 2 | 0.18 | 0.014 |
