# Supplementary material for: Analysis of the in planta transcriptome expressed by the corn pathogen Pantoea stewartii subsp. stewartii via RNA-Seq
Source: PeerJ. 2017 Apr 27;5:e3237. doi: 10.7717/peerj.3237 (PMC5410145; doi:10.7717/peerj.3237)
Supplement: Table S10 [file peerj-05-3237-s010.docx]

**Table S10.** GO gene groups from four-fold regulated genes in the *in planta* culture compared to the *in vitro* plate culture.

| **GO.ID** | **Term** | **Annotated** | **Significant** | **Expected** | **weight01Fisher** |
| --- | --- | --- | --- | --- | --- |
| **Upregulated *in planta*** | | | | | |
| GO:0009401 | phosphoenolpyruvate-dependent sugar phosphotransferase system | 34 | 12 | 3.45 | 6.50E-05 |
| GO:0055114 | oxidation-reduction process | 164 | 33 | 16.62 | 9.00E-05 |
| GO:0009098 | leucine biosynthetic process | 4 | 4 | 0.41 | 0.00010 |
| GO:0006810 | Transport | 510 | 88 | 51.7 | 0.00021 |
| GO:0009405 | Pathogenesis | 17 | 7 | 1.72 | 0.00081 |
| GO:0006777 | Mo-molybdopterin cofactor biosynthetic process | 9 | 5 | 0.91 | 0.00092 |
| GO:0006012 | galactose metabolic process | 4 | 3 | 0.41 | 0.0038 |
| GO:0015689 | molybdate ion transport | 4 | 3 | 0.41 | 0.0038 |
| GO:0006064 | glucuronate catabolic process | 2 | 2 | 0.2 | 0.010 |
| **Downregulated *in planta*** | | | | | |
| GO:0009435 | NAD biosynthetic process | 6 | 2 | 0.09 | 0.0031 |
| GO:0006323 | DNA packaging | 8 | 2 | 0.12 | 0.0058 |
| GO:0009228 | thiamine biosynthetic process | 8 | 2 | 0.12 | 0.0058 |
